# An Atypical Kinase under Balancing Selection Confers Broad-Spectrum Disease Resistance in Arabidopsis

**DOI:** 10.1371/journal.pgen.1003766

**Published:** 2013-09-12

**Authors:** Carine Huard-Chauveau, Laure Perchepied, Marilyne Debieu, Susana Rivas, Thomas Kroj, Ilona Kars, Joy Bergelson, Fabrice Roux, Dominique Roby

**Affiliations:** 1 INRA, Laboratoire des Interactions Plantes-Microorganismes (LIPM), UMR441, Castanet-Tolosan, France; 2 CNRS, Laboratoire des Interactions Plantes-Microorganismes (LIPM), UMR2594, Castanet-Tolosan, France; 3 Laboratoire de Génétique et Evolution des Populations Végétales, UMR CNRS 8198, Université des Sciences et Technologies de Lille, Lille, Villeneuve d'Ascq, France; 4 Department of Ecology and Evolution, University of Chicago, Chicago, Illinois, United States of America; Virginia Tech, United States of America

## Abstract

The failure of gene-for-gene resistance traits to provide durable and broad-spectrum resistance in an agricultural context has led to the search for genes underlying quantitative resistance in plants. Such genes have been identified in only a few cases, all for fungal or nematode resistance, and encode diverse molecular functions. However, an understanding of the molecular mechanisms of quantitative resistance variation to other enemies and the associated evolutionary forces shaping this variation remain largely unknown. We report the identification, map-based cloning and functional validation of *QRX3* (*RKS1*, *Resistance related KinaSe 1*), conferring broad-spectrum resistance to *Xanthomonas campestris (Xc)*, a devastating worldwide bacterial vascular pathogen of crucifers. *RKS1* encodes an atypical kinase that mediates a quantitative resistance mechanism in plants by restricting bacterial spread from the infection site. Nested Genome-Wide Association mapping revealed a major locus corresponding to an allelic series at *RKS1* at the species level. An association between variation in resistance and *RKS1* transcription was found using various transgenic lines as well as in natural accessions, suggesting that regulation of *RKS1* expression is a major component of quantitative resistance to *Xc*. The co-existence of long lived *RKS1* haplotypes in *A. thaliana* is shared with a variety of genes involved in pathogen recognition, suggesting common selective pressures. The identification of *RKS1* constitutes a starting point for deciphering the mechanisms underlying broad spectrum quantitative disease resistance that is effective against a devastating and vascular crop pathogen. Because putative *RKS1* orthologous have been found in other *Brassica* species, *RKS1* provides an exciting opportunity for plant breeders to improve resistance to black rot in crops.

## Author Summary

During the evolution of plant-pathogen interactions, plants have evolved the capability to defend themselves from pathogen infection by different overlapping mechanisms. Disease resistance is constituted by an elaborate, multilayered system of defense. Among these responses, quantitative resistance is a prevalent form of resistance in crops and natural plant populations, for which the genetic and molecular bases remain largely unknown. Thus, identification of the genes underlying quantitative resistance constitutes a major challenge in plant breeding and evolutionary biology, and might have enormous practical implications for human health by increasing crop yield and quality. Our work contributes to understanding the molecular bases of quantitative resistance to the vascular pathogen *Xanthomonas campestris* (*Xc*), which is responsible for black rot, an important disease of crucifers worldwide. By multiple approaches, we demonstrate that *RKS1* is a quantitative resistance gene in *Arabidopsis thaliana* conferring broad-spectrum resistance to *Xc* and that this resistance mechanism in plants is associated with regulation of *RKS1* expression. We also provide evidence that *RKS1* allelic variation is a major component of quantitative resistance to *Xc* at the species level. Finally, the long-lived polymorphism associated with *RKS1* suggests that evolutionary stable broad-spectrum resistance to *Xc* may be achieved in natural populations of *A. thaliana*.

## Introduction

Pathogens are a threat for crops and natural plant populations. A major challenge in plant breeding and evolutionary biology is to identify the genetic and molecular bases for natural resistance variation in plant species. The identification of genes underlying natural resistance variation might have enormous practical implications by increasing crop yield and quality and gives fundamental insights in the prediction of evolutionary trajectories of natural populations. Disease resistance is constituted by an elaborate, multilayered system of defense [1], and substantial progress has been made in the understanding of plant specific disease resistance conferred by single *R* genes. However there is still very limited information about the genes underlying quantitative resistance, despite the fact that this form of resistance is much more prevalent in crops and natural plant populations than *R* gene specific resistance [2]. Such genes conferring partial resistance to pathogens have been identified only in few cases, all for fungal and nematode resistance, and encode diverse molecular functions underlying durable and broad-spectrum resistance [3]–[7].

How host plants achieve quantitative resistance against other major enemies remains largely unknown [8]–[9]. Biotrophic bacterial pathogens generally trigger extreme defense responses (cell death) that are typically mediated by *R* genes. Identification and understanding of alternative molecular mechanisms (i.e. quantitative resistance) to such enemies, is of high interest both on a basic and applied point of view. It is furthermore unknown whether quantitative resistance genes show hallmarks of selection similar to those for gene-for-gene resistance. *R*-genes often reveal a signature of balancing selection in natural plant species, i.e. long-lived polymorphism of *R* gene alleles [10]–[11]. Identifying the molecular mechanisms of quantitative resistance variation and understanding the evolutionary forces shaping variation in quantitative resistance should inform strategies for long-term broad-spectrum control of bacterial diseases.

*Xanthomonas campestris* pv. *campestris* (*Xcc*) is a biotrophic bacterium infecting the vascular system of plants. *Xcc* causes black rot disease, possibly the most important disease of crucifers [12]. It is also one of the most prevalent bacterial pathogens in natural populations of *Arabidopsis thaliana* [13]. Previous work on the Arabidopsis-*Xcc* interaction revealed that different sources of resistance and tolerance to *Xcc* exist in Arabidopsis [14]–[17], but until now no information is available about the molecular mechanisms underlying this resistance or tolerance.

By multiple approaches, we demonstrate that *RKS1* is a quantitative resistance gene conferring broad-spectrum resistance to several *Xanthomonas campestris* races and pathovars. We show that *RKS1* restricts bacterial spread from the infection site to the vascular system. This novel resistance mechanism in plants is associated with regulation of *RKS1* expression. Moreover, we provide evidence that *RKS1* allelic variation is a major component of quantitative resistance to *Xc* at the species level. Finally, a signature of balancing selection acting on *RKS1* indicates that evolutionary stable broad-spectrum resistance to *Xc* may be achieved in natural populations of *A. thaliana*.

## Results

### A major QTL *QRX3* confers resistance to the strain *Xcc568*

**Figure 1:**
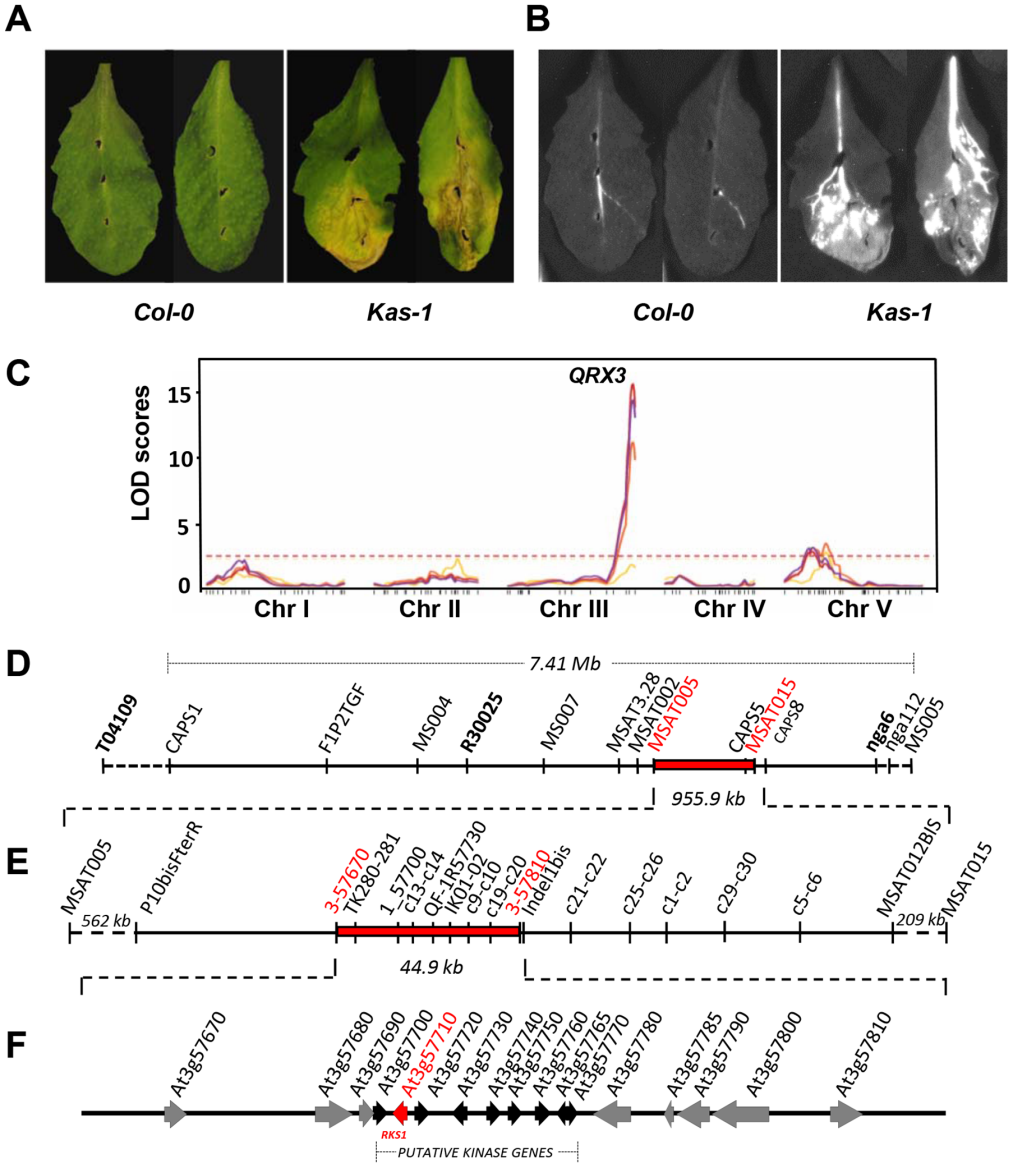
Identification and mapping of the major QTL, *QRX3*, for resistance to *Xcc*. (A and B) Phenotype of susceptible (Kas-1) and resistant (Col-0) accessions (Col-5 and Col-0 (used here) show similar phenotypes) : (A) symptoms 7 days post-inoculation (dpi) and (B) bacterial invasion of leaf tissue using an *Xcc568* reporter strain that carries the *Photorhabdus luminescens* lux operon. (C) QTL maps of resistance to *Xcc* in the Col-5 x Kas-1 recombinant inbred line population at four inoculation times: yellow, 3 dpi; orange, 5 dpi; red, 7 dpi and purple, 10 dpi. The horizontal dotted line represents the significance threshold for the LOD score (average = 2.50). (D to F) Map-based isolation of the *QRX3* locus. (D) Genetic map of chromosome III is shown between markers *T04109* and *MS005* with the defined target interval for *QRX3* (in red). (E) A number of additional markers and recombinant lines were used to reduce the *QRX3* locus to a 44.9 kb region between the markers *3-57670* and *3-57810*. (F) The corresponding physical interval contains 17 open reading frames (ORFs). Genes are represented by arrows. The black arrows correspond to a cluster of putative kinase genes, the red arrow corresponds to *RKS1*.

We subjected natural accessions of *A. thaliana* to infection by the *Xanthomonas campestris* pv. *campestris* (*Xcc*) strain 568. Substantial genetic variation for resistance to this strain was revealed by inoculation of a core collection of natural accessions of *A. thaliana* (Figure S1). To characterize the genetic basis of this variation, we carried out QTL (Quantitative Trait Locus) analysis using an F_6_ Recombinant Inbred Line (RIL) population of 115 lines derived from the accessions Columbia *glabrous1* (Col-5) and Kashmir-1 (Kas-1) which exhibit contrasting phenotypes (Figures 1A and B) [18]. Three different experiments were conducted: a disease index was scored for both growth chamber and greenhouse grown 28-day old plants and *in planta* bacterial growth was measured using growth chamber grown plants. In each of those three independent experiments, the QTL analysis revealed one major QTL at the bottom of chromosome 3, *QRX3* (*Quantitative Resistance to Xcc568*) that explained up to 53.7% of the phenotypic variance (Figure 1C, Table S1). Four minor QTLs were also detected in some experiments, on chromosomes 1 (*QRX1.1* and *QRX1.2*), 2 (*QRX2*) and 5 (*QRX5*) (Table S1). The allelic additive effects of these QTLs were in the same direction, the Col-5 allele increasing resistance compared to the Kas-1 allele.

The effect of *QRX3* was validated in heterogeneous inbred families (HIFs); all homozygous HIF lines that carried the Kas-1 allele were susceptible to *Xcc568* and all homozygous lines that carried the Col-5 allele were resistant, reflecting perfectly the parental phenotypes (Figure S2). These results confirm the quantitative contribution of this major locus to *Xcc568* resistance. We thus selected *QRX3* as a target for map-based cloning.

### The *At3g57710 (RKS1)* gene identified by map-based cloning underlies *QRX3*

**Figure 2:**
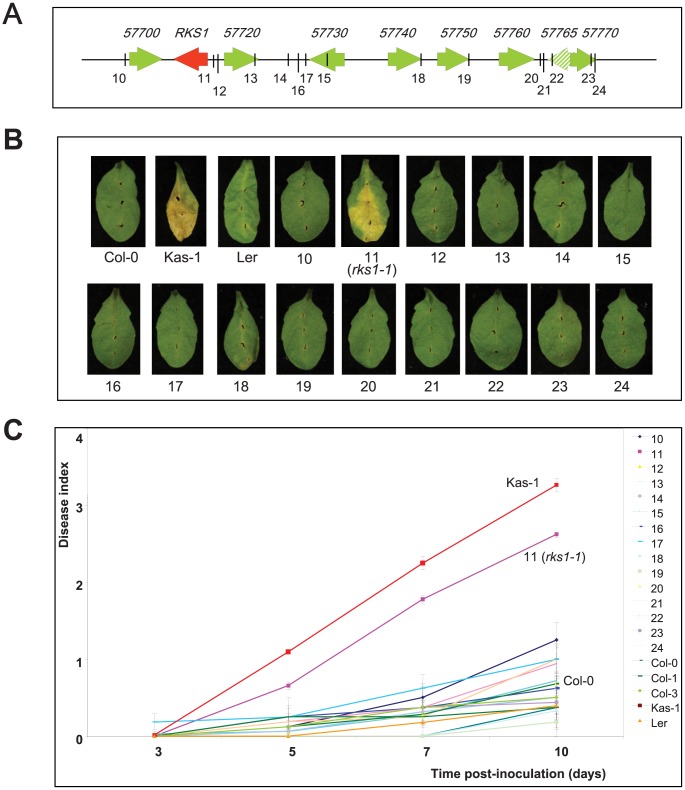
Phenotypic analysis of insertional mutants corresponding to genes of the *QRX3* locus. (A) Structure of the kinase cluster contained within the *QRX3* locus and positions of the insertional mutations are indicated with vertical lines. (B) Disease symptoms were observed on leaves of mutant and wild-type plants, 10 days post-inoculation with a bacterial suspension adjusted to 2×10^8^ cfu/mL. (C) Time course evaluation of disease index after inoculation with *Xcc568* under the same conditions. Means and standard errors were calculated from 3–8 plants.

**Figure 3:**
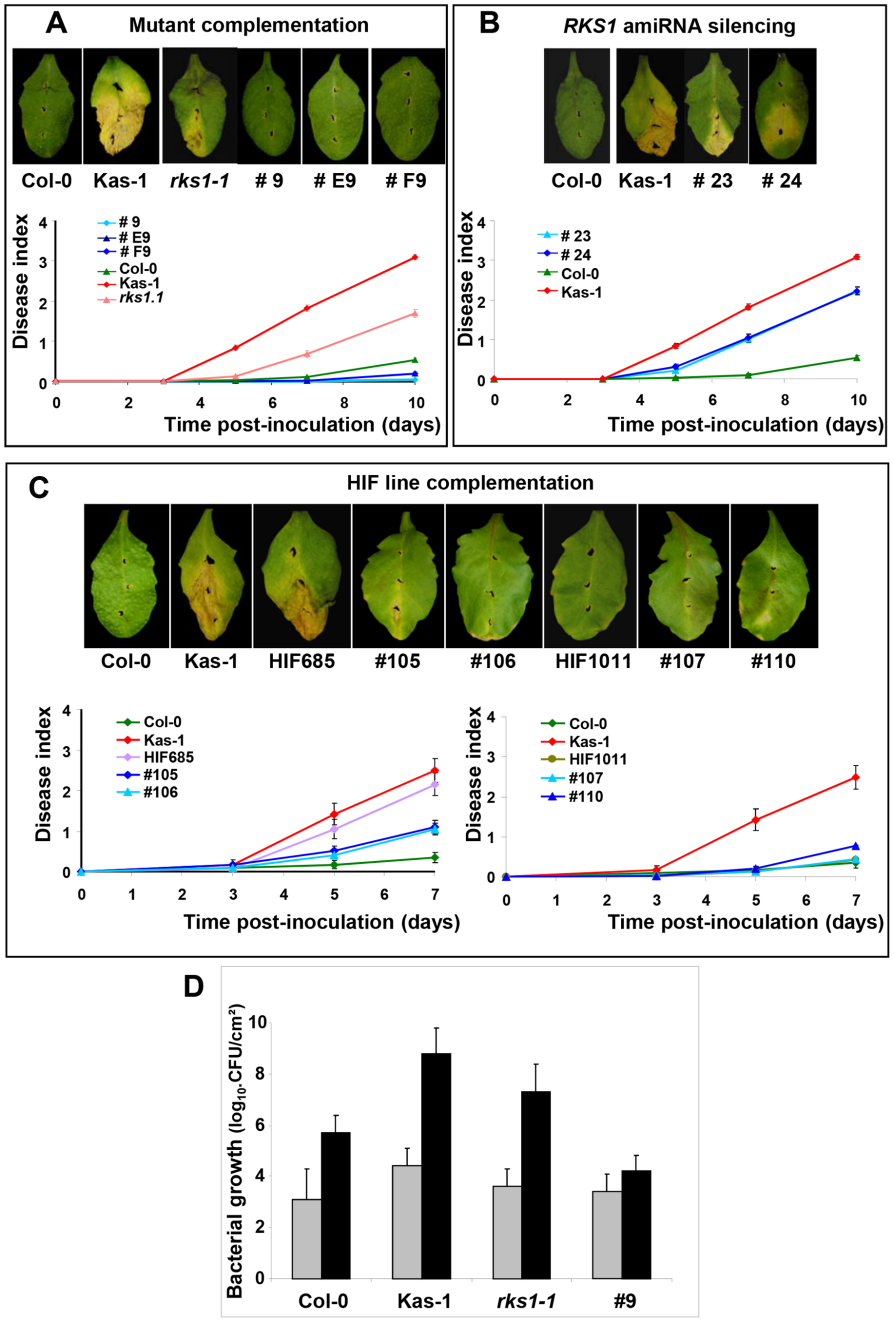
Genetic evidence that *RKS1* is causal for *QRX3* QTL. Disease symptoms were observed on leaves of wild-type plants, mutants, HIF lines or lines complemented with the *RKS1* gene, at 7 (C) or 10 (A and B) days post-inoculation. Time course evaluation of our disease index was performed after inoculation with *Xcc568* under the same conditions. (A) Mutant complementation (lines #9, #E9, #F9). (B) amiRNA silencing (lines #23 and #24). (C) HIF line complementation (lines #105 and #106 for the susceptible HIF (HIF685), lines #107 and #110 for the resistant HIF (HIF1011)). Means and standard errors were calculated for 16–60 plants (4–9 independent experiments). (D) Bacterial growth measurement (colony forming unit (CFU)/cm^2^ expressed in a log10 scale) in leaves of lines differing only by the presence of *RKS1* gene (wild type (Col-0), *rks1-1* mutant, and the complemented mutant line (#9)). The susceptible accession Kas-1 has been included as a positive control. Bacterial growth has been measured 0 (grey bars) and 7 (black bars) days after inoculation with *Xcc* strain 568. Data were collected from two independent experiments, each timepoint corresponds to 6 independent measurements, each on 3–5 individual plants (four leaves/plant).

On the basis of three high-resolution populations, we reduced the target interval to a 44.9 kb region (Figure 1D and E, Tables S2 and S3), containing 17 predicted Open Reading Frames (ORFs) (Figure 1F). To identify the gene(s) implicated in resistance to *Xcc568*, one to six insertional mutants (T-DNA or transposon mutant lines) were selected for each gene within the narrowed region (Table S4). Of the 36 mutants characterized, only one (mutant 11/*rks1-1*) showed a susceptible phenotype, albeit less pronounced than the susceptible Kas-1 control line (Figures 2 and 3A). In *rks1-1*, the T-DNA insertion was located in the intergenic region between *At3g57710* (herein termed *RKS1*, for *Resistance-related KinaSe1*) and *At3g57720*. These two genes encode putative protein kinases that belong to a cluster of putative kinases (spanning *At3g57700* to *At3g57770*; Figure 1F). Complementation of *rks1-1* with a 2.4 kb genomic fragment containing *RKS1* from the resistant accession Col-0 (Figure S3) led to complete restoration of resistance (Figure 3A). These results were confirmed by evaluation of bacterial growth *in planta*, using a procedure adapted to the biology of the bacterial pathogen *Xcc* (Figure 3D), showing that RKS1 is involved in the resistance to bacterial colonization, a finding particularly meaningful as *Xcc* is a vascular pathogen.

A gene silencing approach, *via* artificial microRNA (amiRNA), confirmed that *RKS1* rather than *At3g57720*, is responsible for quantitative resistance to *Xcc568*. In particular, amiRNA lines for *RKS1* in the resistant background Col-0 were susceptible to *Xcc568*, whereas silencing of *At3g57720* had no effect on the resistant phenotype (Figure 3B, Figure S4). As expected, transformation of the original HIF (HIF685) containing the Kashmir-1 susceptible allele with the resistant (Col-0) allele of *RKS1* restored a high level of resistance in the plants (Figure 3C left, Figure S3), whereas introduction of the same construct into the original resistant HIF containing the Columbia allele (HIF1011) did not lead to a change in the level of resistance (Figure 3C right, Figure S3). We also transformed the resistant accession Col-0 and resistant HIF1011 with the susceptible (Kas-1) allele of *RKS1*, and found a minor but significant increase in susceptibility (Figure S5). Taken together these data indicate that *QRX3* can be explained by *RKS1* allelic variation.

### The *RKS1* gene (*At3g57710*) encodes an atypical kinase

**Figure 4:**
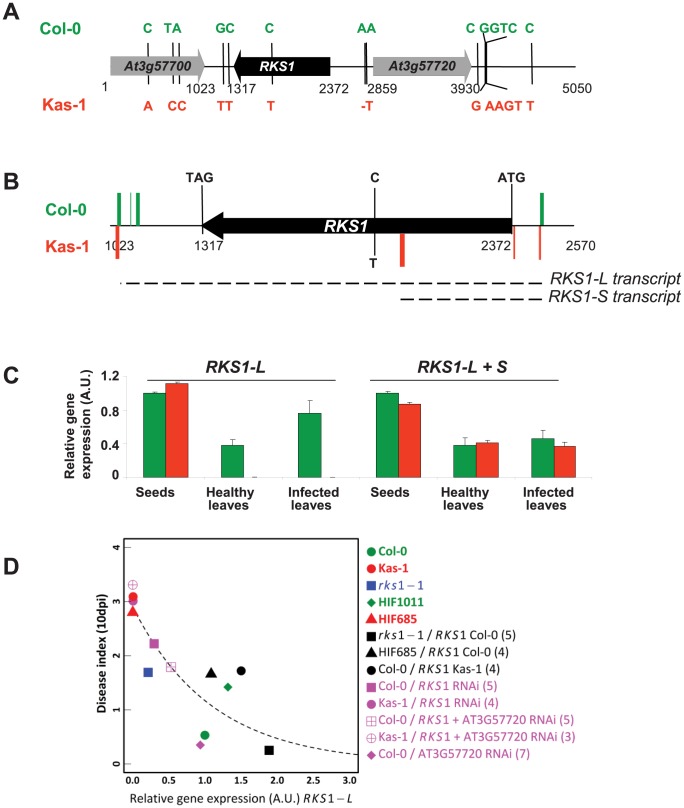
*RKS1* allelic forms and expression in susceptible and resistant accessions of *Arabidopsis thaliana*. (A) Schematic representation of resistant (Col-0) and susceptible (Kas-1) *RKS1* allele polymorphisms. Sequence changes in both alleles are indicated. (B) Schematic representation of the most frequent 5′ and 3′ ends of *RKS1* transcripts found by 5′ and 3′ RACE experiments in resistant (Col-0, green) and susceptible (Kas-1, red) accessions. (C) *RKS1* gene expression evaluated by Q-RT-PCR in germinating seeds and in leaves, healthy or inoculated with *Xcc568*, from the resistant accession (Col-0, green) and the susceptible accession (Kas-1, red). Different primers are used to evaluate long (L) and long+short (L+S) transcripts (Table S2) A.U.: arbitrary units. (D) Correlation between *RKS1* gene expression after infection with *Xcc568* and resistance phenotype. The dashed line indicates an exponentially decreasing function fitted on the median values of the 13 types of genetic line. Numbers in brackets indicate the number of representatives of each type of transgenic line.

*RKS1* encodes a predicted protein of 351 amino acids with an estimated molecular mass of 39.9 kDa (Figure 4A). BlastP analysis of the *RKS1* sequence from Col-0 identified several hits corresponding to protein kinase-like or putative protein kinases. However, closer analysis of the RKS1 protein sequence suggests that RKS1 is an atypical kinase, since it lacks some critical domains in the kinase catalytic core that are essential for catalysis (Figure S6A). Only the His-Arg-Asp (HRD) motif, with the catalytic Asp residue that functions as a base acceptor to achieve proton transfer, is present in RKS1. In contrast, the glycine loop (GxGxxG), which binds and positions ATP, is not present in RKS1. Moreover, the presence of an Asp residue in the glycine loop has been reported to provide an acidic environment that inhibits ATP binding [19]. Second, the Val-Ala-Val-Lys (VAVK) motif, in which the Lys residue interacts with, anchors and orients the α and β phosphates of ATP, is not well conserved in RKS1. Third, the two tripeptide motifs in the activation segment [Asp-Phe-Gly (DFG) and Ala-Pro-Glu (APE)] are highly modified. In the DFG domain, only the Asp residue, responsible for chelating the Mg^2+^ ion that positions the phosphates for phosphotransfer [20] is present. Consistent with the absence of necessary domains for kinase activity, we were unable to detect any kinase activity displayed by RKS1 despite the wide range of experimental conditions tested: protein production and purification from *E. coli* or plant tissues; autophosphorylation assays or phosphorylation of a variety of substrates including MBP (myelin basic protein), casein and histone, in the presence of different ions (Ca^2+^, Mg^2+^, Mn^2+^…) and using buffers with different pH (from 5.5 to 9.5) (Figures S6B and C). Interestingly, atypical kinases (or pseudokinases) have been described as important regulators of signalling networks. Their mode of action is thought to involve acting as molecular scaffolds for assembly of multiprotein complexes or modulation of the activity of a catalytically active enzyme [21]–[22]. Sequencing of a 5kb region centered on *RKS1* for Col-5 and Kas-1 revealed a single-SNP difference in the coding region resulting in an amino acid change in the activation segment relative to the catalytic kinase loop (Figure S6). Other polymorphisms were found in the 5′ and 3′ regulatory regions of *RKS1* (Figure 4A). This raised the question of the level at which the Kas-1/Col-0 sequence change(s) affect(s) the observed phenotypic variation.

### *RKS1* expression in resistant and susceptible accessions

We next characterized the expression of *RKS1* alleles in Kas-1 and Col-0 during development and in response to infection. Two different transcripts could be identified from 3′ and 5′ RACE experiments and cDNA sequencing. A long transcript (*RKS1-L*) is mainly found in the resistant accession Col-0, while the other short transcript (370 bp, *RKS1-S*) is associated with the susceptible accession (Kas-1) (Figures 4B and S7). While *RKS1-L* was expressed at high levels in germinating seeds and at lower levels in adult leaves (2.6 fold higher in seeds as compared to healthy leaves), it is differentially expressed in leaves. High levels of *RKS1-L* are observed in Col-0 whereas extremely low levels are detected in Kas-1 (Figure 4C)(*RKS1-L* expression is 235.1 fold higher in Col-0 as compared to Kas-1). Leaf infection by *Xcc* did not lead to a significant change in *RKS1-L* expression. These results suggest that *RKS1* leaf expression levels might underlie the *QRX3* QTL. While just a correlation, this hypothesis is reinforced by a significant nonlinear relationship between resistance and *RKS1-L* expression among the natural accessions Col-0 and Kas-1, HIFs, complemented and silenced lines (Figure 4D, Tables S5 and S6).

### *RKS1* confers broad-spectrum resistance

**Figure 5:**
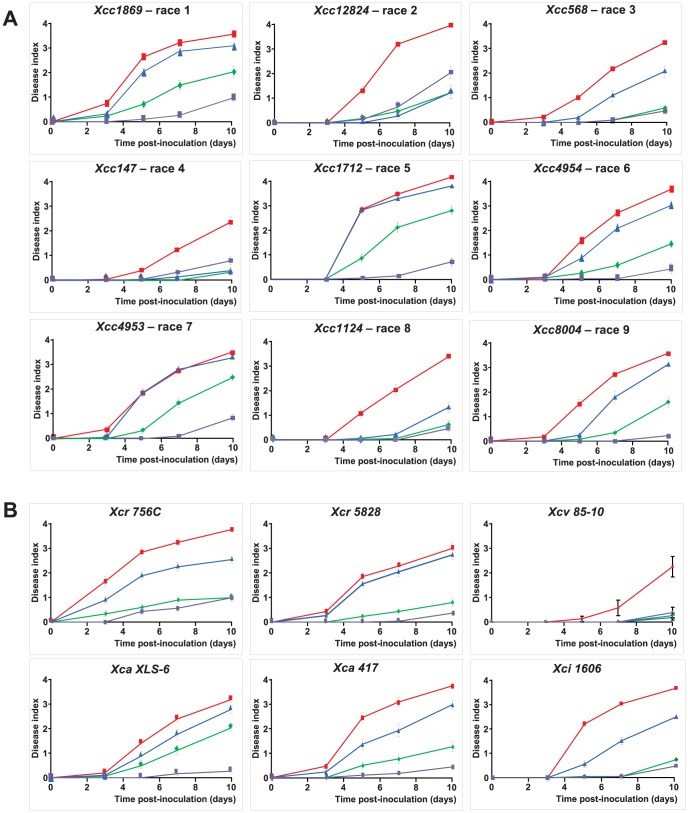
*RKS1* confers resistance to multiple strains and races and pathovars of *Xcc*. Time course evaluation of disease index in lines differing only by the presence of *RKS1* gene (*rks1-1* mutant (blue), complemented *rks1-1* mutant (purple) and the parental lines Col-0 (green) and Kas-1 (red) after inoculation with different strains of (A) *Xcc* belonging to races as defined by Vicente *et al.* [49] and Fargier *et al.* [50], and of (B) *Xc* pathovars *raphani* (*Xcr*), *armoriaceae* (*Xca*), *vesicatoria* (*Xcv*) and *incanae* (*Xci*).

The species *Xanthomonas campestris* includes economically important bacteria with a broad range of hosts within Brassicaceae and Solanaceae, and include three main pathovars (*campestris, raphani, incanae*) [23]. For *Xcc*, 9 races (each including different strains) have been proposed by Fargier and Manceau [24] based on the reaction of different *brassica* species. *RKS1*-conferred resistance is effective not only against the strain *Xcc568*, belonging to race 3, but also against strains in each of four additional races (races 1, 5, 7 and 9; Figure 5A). In addition, by testing different strains from race 6, *RKS1* was effective against one of 3 strains tested (*Xcc4954*). Interestingly, *RKS1* was also found to confer resistance to additional pathovars of *X. campestris*: *raphani*, *armoriaceae* and *incanae* (Figure 5B). These results demonstrate that *RKS1* confers broad spectrum resistance to *Xanthomonas campestris* including multiple strains belonging to most of the known races as well as other pathovars.

### Nested GWA mapping suggests an allelic series at *RKS1*

**Figure 6:**
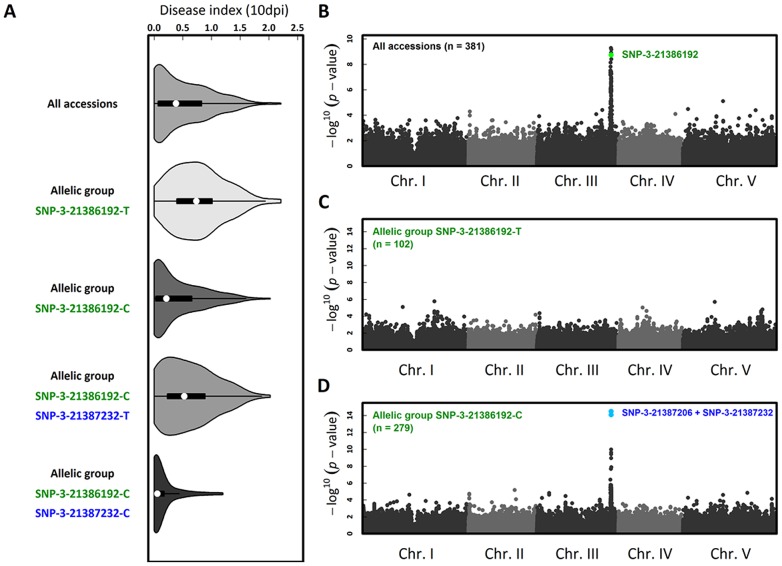
The genetics of *Xcc568* quantitative resistance at the species level identified by nested GWA mapping. (A) Violin plots (i.e. box-and-whisker plot overlaid with a kernel density plot) of phenotypic variation of our disease index. Whole-genome scan of 214,051 SNPs for association with disease index at 10 dpi across (B) 381 accessions, (C) within the allelic group SNP-3-21386192-T and (D) within the allelic group SNP-3-21386192-C.

To investigate whether natural variation for quantitative resistance to *Xcc568* is caused by *RKS1* at the species level, we phenotyped 381 natural accessions that constitute a GWA (Genome Wide Association) mapping population in *A. thaliana*. In order to study natural variation for quantitative resistance to *Xcc568* at different geographical scales, this set of accessions includes both worldwide accessions and French accessions corresponding to the French RegMap that contains two highly genetically polymorphic natural populations (MIB and TOU). We observed extensive phenotypic variation for a disease index, with a prevalence of resistant accessions (Figure 6A, Table S7) from a worldwide to a local scale (Table S7). GWA mapping revealed a unique large peak of association on chromosome 3 that overlaps with *RKS1* (Figure 6B, Figures S8, S9 and S10), with the fourth most associated SNP (Single Nucleotide Polymorphism) corresponding to a single SNP in the 3′ region of *RKS1* (i.e. SNP-3-21386192). However, even after splitting the accessions by the C/T polymorphism at this SNP, substantial phenotypic variation for resistance persists within both allelic groups (Figure 6A). While no obvious association peak was found within the more susceptible (S) allelic group SNP-3-21386192-T (Figure 6C), a unique peak of association was identified within the relatively resistant (R) allelic group SNP-3-21386192-C (Figure 6D, Figures S9, S10 and S11). The two most associated SNPs (i.e. SNP-3-21387206 and SNP-3-21387232) underlying this peak are located at the beginning of *RKS1*. Nested GWA mapping thus suggests a second susceptible allele SNP-3-21387232-T segregating within the R allelic group SNP-3-21386192-C that is also within or near *RKS1*. The three allelic groups explained 43.8% and 47.1% of natural variation in the local populations TOU (n = 71) and MIB (n = 50), respectively.

### The co-existence of long lived *RKS1* haplotypes supports the ecological and evolutionary importance of *RKS1*

**Figure 7:**
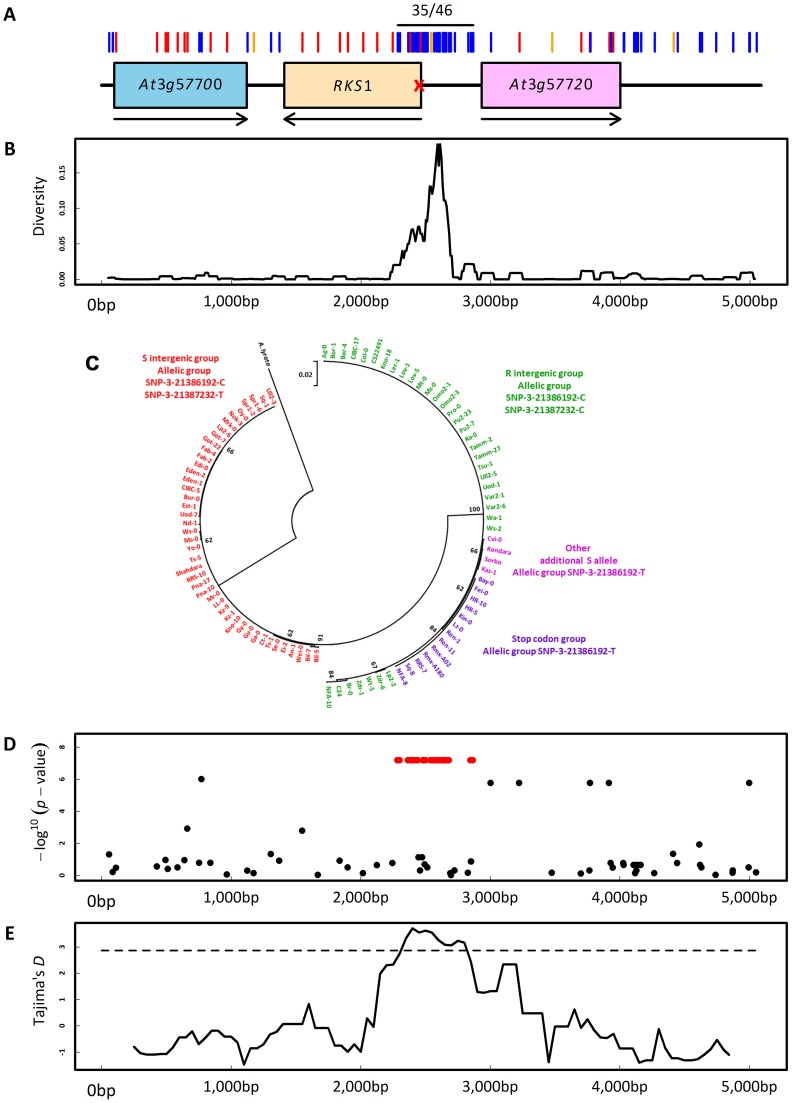
Molecular evolutionary genetics. (A) Sequence diversity in the genomic region centered on *RKS1*. Red, blue and orange bars indicate non-synonymous mutations, silent mutations and indels, respectively. ‘35/46’ indicates the location of the 35 SNPs in complete linkage disequilibrium. The red cross indicates the position of the stop codon at the fourth amino-acid. Arrows indicate the orientation of the genes along the genome. (B) Observed nucleotide diversity between the two intergenic haplotypes. (C) Maximum Likelihood circular tree based on nucleotide variation of *RKS1* and the intergenic region between *RKS1* and *At3g57720*. (D) Scan for association with the relative gene expression of *RKS1-L* in the sequenced region centered on *RKS1*. Red points indicate the 35 SNPs in complete LD. The *y*-axis indicates the –log^10^ *p*-values using the EMMAX method. (E) Tajima's *D* values along the 5 kb genomic region centered on *RKS1*. The dashed line denotes the 1% significance level of an empirical distribution based on 876 short fragments obtained for the same set of accessions [63].

As for Col-0 and Kas-1 (Figure 4A), we sequenced a 5 kb region centered on *RKS1* for 95 accessions (Table S8). Ninety-nine polymorphisms were revealed along the 5 kb region (Figure 7A). Interestingly, the region encompassing the first 186 bases of *RKS1* and the intergenic region between *RKS1* and *At3g57720* defined two highly divergent haplotypes distinguished by 35 polymorphisms in complete linkage disequilibrium (LD, two non-synonymous and seven synonymous mutations in the first 186 bases of *RKS1*, 21 mutations and five indels (1 bp to 17 bp) in the intergenic region between *RKS1* and *At3g57720*; Figures 7A and 7B). The most and less divergent haplotypes from *A. lyrata* and *Brassica rapa* (n = 51; Figure 7C and Figure S12) correspond to the resistant allele SNP-3-21387232-C and the second susceptible allele SNP-3-21387232-T detected by our approach of nested GWA mapping, respectively. Sequence analysis further revealed that our initial highly susceptible allelic group SNP-3-21386192-T (Figure 6A) is composed of two independent susceptible alleles within the R haplotype (n = 51; Figure 7C and Table S8). The first one corresponds to a stop codon at the fourth amino acid of *RKS1* whereas the second one including Kas-1, contains several polymorphisms in complete LD that are scattered across the 5 kb region (Figure 4A, Table S8).

Consistent with the expression analysis above, our disease index was significantly negatively correlated with expression of *RKS1*-*L* among natural accessions (Figure S13, Tables S9 and S10). It was not associated with expression of *At3g57720*, used as a control (Figure S14). The R haplotype was strongly associated with a higher level of *RKS1* expression as measured using either total mRNA or *RKS1-L* (Figure 7D, Figure S15). These results again suggest that regulation of *RKS1* expression is a major component of quantitative resistance to *Xcc568*.

A signature of balancing selection acting on the two highly divergent haplotypes was suggested by significant positive values of Tajima's *D* (Figure 7E) and the presence of polymorphic populations across Europe (Figure S16A). In a survey of 476 accessions of the R haplotype, the second highly susceptible allele including Kas-1 is rare (n = 9) whereas the first highly susceptible allele associated with a stop codon is common throughout Western Europe (n = 162; Figure S16B).

## Discussion

The understanding of the molecular mechanisms of quantitative resistance variation in general, and of quantitative resistance to biotrophic bacterial pathogens, remains largely unknown. Our work contributes to elucidating the molecular bases of quantitative resistance to the vascular pathogen *Xanthomonas campestris*, which is responsible for black rot, possibly the most important disease of crucifers worldwide. By analysing natural variation of resistance in *Arabidopsis thaliana*, we identified and cloned the gene *RKS1* underlying a major QTL and conferring broad-spectrum resistance to *Xc*.

### *RKS1* confers quantitative resistance to *Xcc*

Resistance conferred by the *RKS1* resistant allele is quantitative, as shown by different lines of evidence. First, *RKS1* confers partial resistance to *Xcc*, and disease is only reduced (not absent) in presence of *RKS1* alone. Second, levels of resistance of diverse accessions fit along a continuum measured by disease index (Figure S1). Third, *RKS1* alone confers substantial resistance in the susceptible background, but stronger resistance is evident in combination with other *QRX* loci (other minor QTLs have been identified, each explaining 10 to 15% of the variability, Table S1). Fourth, introduction of an extra copy of the allele for susceptibility reduces resistance, as expected if the mechanism of action is quantitative (Figure S5). This latter observation is in good agreement with the intermediary level of resistance observed in heterozygous HIF lines (Figure S2), and can be explained by co-dominance of the two alleles. Molecularly, a possible explanation for this phenotype should consider, beyond the level of expression, the functionality of the two proteins, since a mutation is present in the Kas-1 coding region. The susceptible allele might act in competition with the resistant allele for interacting (directly or indirectly) with the same factors for triggering the resistance pathways, and might neutralize a part of the effect of the resistant allele. Another important feature of RKS1-conferred resistance is the absence of hypersensitive response (HR), as expected for a quantitative gene (for which the resistance response is less extreme than in the case of a gene-for-gene interaction). *RKS1* is rather involved in the restriction of bacterial spread from the infection site (Figure 3), without visible cell death phenotype. This resistance mechanism may be particularly adapted to counteract the infection strategy of the vascular bacterial pathogen *Xanthomonas*.

As reported by Poland *et al.* and others [2], [25]–[26], quantitative resistance is not as well understood as qualitative resistance. It should also be mentioned that host resistance may not always be described as either qualitative or quantitative, and a gray zone may exist between these two categories [2]. For instance, it has been hypothesized that some forms of quantitative resistance may involve weak *R* genes [2]. Our results clearly indicate that this is not the case for *RKS1*. In favour of this assumption, the first cloned genes underlying QTLs are all structurally different from *R*-genes and do not encode LRR or similar protein motifs. They all encode diverse functions: *Pi21* encodes a metal transport/detoxification protein involved in plant defense [5], *LR34* encodes a putative ABC transporter [3], and *Yr36*, a kinase containing a putative START lipid binding domain [4]. The two recently cloned QTLs conferring resistance to nematodes, correspond for one of them, to a serine hydroxymethyltransferase [7], and for the other, to an aminoacid transporter, a α-SNAP protein and a WI12 (wound inducible domain) protein [6]. This is in agreement with the varied biological bases hypothesized for quantitative resistance [2]. *RKS1*, encoding an atypical kinase, represents a new category of function, putatively involved in signal transduction.

In summary, RKS1 appears to be a new quantitative gene conferring resistance to a vascular bacterial pathogen, *X. campestris*, for which the molecular bases were unknown, and that differs significantly in its phenotypic behaviour from *R*-gene mediated resistance.

### *RKS1* encodes an atypical kinase

Plants and animals sense invasion of potential pathogens using recognition receptors and launch cascades of immune responses that are critical for fitness and survival [27]. Protein kinases are involved in orchestrating these responses by integrating cell signaling networks. *RKS1* encodes an atypical kinase, since (i) it lacks some critical domains in the kinase catalytic core that are essential for catalysis (Figure S6), and (ii) no kinase activity displayed by RKS1 could be detected despite the wide range of experimental conditions tested. This raises the question of the nature of RKS1 function: inactive pseudokinase or simply unusual active kinase? Even if three conserved kinase motifs are modified in RSK1, this does not demonstrate missing catalytic activity. Several kinases previously categorized as pseudo-kinases solely based on primary amino acid sequence analysis, have been later found to be active kinases [28]–[30]. A detailed functional study of RKS1 through different mutational analyses, including the effect of mutations in the conserved catalytic amino acids (HRD) on resistance, would be of high interest. If RKS1 reveals to be a pseudokinase, known to form complexes with active kinases [21], [22], it would be of special interest to identify members of the RKS1 complex. Interestingly, a type III effector (AvrAC) from *Xcc* has been recently shown to inhibit plant immunity by specifically targeting Arabidopsis *BIK1* and *RIPK*, two receptor-like cytoplasmic kinases known to mediate immune signalling [31]. Implication of *RKS1* in, or downstream of, this signalling pathway, possibly interacting with *BIK1* and *RIPK*, constitutes an attractive hypothesis.

### *RKS1* confers broad-spectrum resistance to *Xc*

In the literature, broad-spectrum resistance refers in general to “resistance to multiple isolates/races of pathogens” [26], and quantitative resistance is generally presumed to be non-race specific, while *R*-mediated resistance is usually race-specific [25]. Although several lines of evidence challenge this dogma (e.g. some quantitative loci show race-specific resistance, while single resistance genes such as *Rpg1* or *mlo* are associated with resistance to multiple isolates/races of pathogens [2]), a number of quantitative disease resistance genes/loci have been shown to confer broad-spectrum resistance. *RFO1* confers resistance to three crucifer-specific fsp of *Fusarium oxysporum* [32], *RB* mediates resistance to all known races of *Phythophthora infestans*, the late blight pathogen [33]; the *RCT1* gene from *Medicago truncatula* confers broad-spectrum resistance to races 1, 2 and 4 of *Colletotrichum trifolii* [34]; Wheat *Yr36* provides high temperature-dependent QR to 8 stripe rust races (6 fully, 2 intermediary phenotype) [4]; Lr34 provides resistance to 2 rust diseases of wheat *Puccinia striiformis* and *Puccinia triticina* [3]. In this context, *RKS1* belongs to this category of genes/loci, conferring resistance to different races and all pathovars of *Xanthomonas campestris*. However, similarly to other broad-spectrum resistance genes/QTLs, RKS1 does not confer resistance to other groups of pathogens, such as *Ralstonia solanacearum* (strain GMI1000), *Sclerotinia sclerotiorum* (S55 strain), *Hyaloperonospora parasitica* (strain Cala2), *Erwinia carotovora* (strain SCC1) or *Alternaria brassicicola* (data not shown). *RKS1* cannot therefore be compared to genes like *NDR1*, *EDS1* or *WRKY33* that condition multiple disease resistance, and are involved in effector-triggered immunity (ETI) or PAMP triggered immunity (PTI) pathways. Although the resistance pathways involved in *RKS1* mediated resistance are not known yet, we can hypothesize that this gene may be a common, downstream component of the signalling pathway triggered by numerous *R* genes involved in Xanthomonas resistance, controlling in part the resistance response mediated by these *R* genes. Alternatively, *RKS1* may function in a resistance pathway depending on the recognition of a general signal/effector from *Xc*.

### *RKS1* expression level as a major component of resistance?

The phenotypic effect of a QTL might be due to one or more causal SNPs, which can be located either in the coding region or in the regulatory regions, affecting in this case the levels, timing and/or tissue-specificity of gene expression. In the case of the resistance QTLs recently cloned, the polymorphisms distinguishing the resistant allele from the susceptible one, are located in the coding region for most of them, with the exception of *Yr36* which is absent in susceptible accessions, and *Rhg1*, for which resistance is conferred by copy number variation [4], [6].

In our study, we found a significant negative relationship between disease index and the expression of *RKS1* long transcript using various transgenic lines as well as in natural accessions, suggesting an essential role of the regulation of *RKS1* expression in mediating quantitative resistance to *Xc*. Interestingly, our nested GWA mapping approach suggests that the causal mutation(s) underlying the regulation of the expression of *RKS1* long transcript might differ among alleles associated with susceptibility. First, while *RKS1* total mRNA expression (assessed by *RKS1-L+S* quantitative RT PCR and in agreement with our characterization by RACE experiments) is similar between the resistant accession Col-0 and the susceptible accession Kas-1, *RKS1* long and short transcripts are specifically expressed in leaves of the Col-0 and Kas-1, respectively (Figure 4), suggesting a post-transcriptional regulation of *RKS1* in Kas-1. Different polymorphisms were found in the regulatory regions between these two accessions (Figure 4A), while only one was found in the coding region of *RKS1*. Second, the expression of both *RKS1* total mRNA and *RKS1* long transcript significantly differs among the two highly divergent haplotypes distinguished by 35 various polymorphisms (from point mutations to a 17 bp indel), in complete LD and located in the region encompassing the first 186 bases of *RKS1* and the intergenic region between *RKS1* and *At3g57720* (Figure S13). However, either in the case of a potential post-transcriptional regulation of *RKS1* in Kas-1 or in the case of *RKS1* total mRNA and long transcript differential expression between two highly divergent haplotypes, the causal role of these polymorphisms is unknown. We cannot also exclude the possibility that the phenotype results from the additive effect or interaction of several polymorphisms. To get more insight into the determinism of *RKS1* mediated resistance, the question of the role of the regulatory region will be addressed through complementation tests of the *rks1-1* mutant with different constructs for instance. In addition, the mechanisms governing the transcriptional and post-transcriptional regulation of *RKS1* through a complete and detailed functional analysis would also be the logical extension of our findings.

### Alternative resistance mechanisms share common selective pressures

Like for a number of *R* genes [10]–[11], the ecological and evolutionary importance of *RKS1* is further supported by a signature of balancing selection acting on two highly divergent haplotypes, and the presence of polymorphic populations across the native range of *A. thaliana*. While fitness cost of resistance is often considered as the main explanation for the maintenance of long-lived resistance polymorphisms in natural populations [35], two other non-exclusive hypotheses can be advanced to explain the coexistence of long-lived haplotypes. First, when evolutionary interactions are considered in a spatially realistic context (for example, metapopulations comprising multiple interacting populations and distance-dependent dispersal), theoretical work has shown that genetic polymorphisms in either host resistance or pathogen virulence genes can persist without the necessity of assuming differential fitness effects [36]–[38]. Second, alternative alleles may be maintained because each is beneficial in face of different enemies. For RPP13, a locus previously shown to have strong signatures of balancing selection in *A. thaliana*, different alleles encode different specificities to isolates of *Hyaloperonospora parasitica* [39]. Similarly, RPP8 contains alleles encoding resistance to alternative types of enemies [40]. In this context, no cost is expected when comparing fitness of alternative alleles in the absence of enemies.

Although identifying the opposing forces acting on *RKS1* certainly deserves further investigation, the long-lived polymorphism associated with *RKS1* indicates that evolutionary stable broad-spectrum resistance to *Xc* may be achieved in natural populations of *A. thaliana*. Whether such non-race-specific resistance is widespread across bacterial pathogens of *A. thaliana* remains an open question. Either way, because putative *RKS1* orthologous have been found in other *Brassica* species (Figure S12), *RKS1* provides an exciting opportunity for plant breeders to improve resistance to black rot in crops.

## Materials and Methods

### Plant materials

Plants were grown on Jiffy pots under controlled conditions [41]. To investigate natural variation of resistance to *Xcc*, we used 23 natural accessions of *Arabidopsis thaliana* including a core collection of 16 accessions (http://dbsgap.versailles.inra.fr/vnat/Fichier_collection/Rech_core_coll16.php) [42]. Then we used a set of RILs derived from the cross Kashmir (Kas-1) and Columbia *gl1* (Col-5) [18], [43]. HIFs were obtained as previously described [44]–[45]. To perform Genome-Wide Association (GWA) mapping, a set of 384 natural accessions was used, including 179 worldwide accessions (WA), 188 French accessions (FA) as part of the French RegMap [46] and 17 accessions that are both WA and FA (Table S7). One hundred and twenty-one accessions from the French RegMap correspond to two natural populations from Burgundy. All the 384 natural accessions have been genotyped for 214,051 SNPs evenly spaced across the genome [46].

### Bacterial material

The inoculation tests were done with the sequenced strain LMG568/ATCC33913 (*Xcc568*) [47] carrying the *LUX* operon of *Photorhabdus luminescens* [48] or different strains of *Xcc* belonging to races as defined by Vicente *et al.* [49] and Fargier *et al.* [50]. Broad spectrum resistance was estimated by inoculation tests with different pathovars of *Xc*, pathovars *raphani (Xcr)*, *armoriaceae* (*Xca*), *vesicatoria (Xcv)* and *incanae (Xci) [CIRM-CFBP collection, INRA, France]*. All *Xcc, Xcr, Xca, Xcv and Xci* strains were grown on Kado medium [51]. Cultures of *Xcc568* (*LUX*) were supplemented with 50 mg/mL rifampicin and 25 mg/mL kanamycin.

### Phenotyping

The Col-5 x Kas-1 RIL population was evaluated by one experiment under greenhouse conditions, and two others in a growth chamber [41]. In these experiments, two blocks were designed and for each RIL two plants per block were evaluated for quantitative resistance to *Xcc*. For each experiment, each RIL was evaluated in a completely randomized block design.

Virulence of *Xcc* strains and other pathovars was tested on 28-day old plants after inoculation by piercing and scoring of the symptoms as described [52]. Each strain was tested in at least three separate experiments where Col-0 or Col-5 and Kas-1 were inoculated as controls.

*In planta* bacterial growth analysis (colony forming unit (CFU)/cm^2^ expressed in a log10 scale) was performed as described by Froidure *et al.* [53]. Due to the specificity of *Xcc*, a vascular bacteria, bacterial growth has been measured 0 and 7 days after inoculation by piercing with *Xcc* strain 568, at distance from the inoculation zone (at the tip of the inoculated leaves). Data were collected from two independent experiments, each time point corresponds to 6 independent measurements, each on 3–5 individual plants (four leaves/plant). At the inoculation site (basis of the inoculated leaves), bacterial growth was measured and found similar in the different lines (4.9±0.5 to 5.1±0.4 CFU/cm^2^ at T0; 8.9±0.5 to 9.2±0.6 CFU/cm^2^ at 7 dpi).

For data reported in Figure 3D, and according to parametric and non parametric tests, respectively ANOVA (df = 3; mean squares = 47.202 ; *F* = 53.887 ; *P*<0.001) and Kruskal-Wallis test (df = 3 ; Kruskal-Wallis test statistic = 34.136 ; *P*<0.001), the genetic background of the lines (Col-0, Kas-1, *rks1-1* and complemented line) explained significantly (81%) the phenotypic variation observed at 7 dpi. The genetic background effect of lines analyzed by pairwise comparisons (Dwass-Steel-Chritchlow- Fligner test) was also significant with a *p*-value below 0.001 for all pairs, except for the couple mutant *rks1-1* and Kas-1 with a marginal *p*-value of 0.051.

### Genome-wide association mapping

An experiment with 1,760 plants was set up according to a completely randomized design involving four experimental blocks, each block being an independent randomization of one replicate per accession. Infected plants were placed in plastic mini-greenhouses, including two control accessions, Col-5 (resistant) and Kas-1 (susceptible), in the same positions within each mini-greenhouse. Mini greenhouses were placed in phytotrons (22°C, 9 h photoperiod, 100% humidity).

### QTL fine mapping

QTL fine mapping was performed by developing HIFs of the RIL900, and by screening the individuals with the *T04109*, *R30025* and *nga6* markers that flank the *QRX3* QTL. One individual, called HIF900-1 and heterozygous for these markers, was selfed. Three hundreds and seventy-six lines were screened with 12 additional markers located within the *QRX3* interval of 7.41 Mb (Figure 1D). The region was then reduced to 4.6 Mb localized between *MS004* and *nga6* markers. To further refine the position of *QRX3*, 38 lines showing recombination events between these markers were phenotyped and screened for all markers available in a 4.6 Mb interval (Table S2). *QRX3* was narrowed down to a 955.9 kb interval delimited by markers *MSAT005* and *MSAT015* (Figure 1E). Among these 38 recombinant lines, the HIF 900-1-368 was selected and its progeny was tested (1408 lines and 2 markers). *QRX3* was mapped to a 44.9 kb region delimited by markers *3_57670* and *Indel1bis* (Figure 1E and Table S3). This region contains 17 predicted Open Reading Frames (ORFs) (Figure 1F).

### Constructs and plant transformation

The plasmids used in this study were constructed with the Gateway technology (GW; Invitrogen) [53]. For complementation assays, genomic fragments containing an 811 bp non-coding region upstream of *RKS1*, *RKS1* coding sequence (1056 bp) and 491 bp downstream of *RKS1* were amplified by PCR using Col-0 genomic DNA with *attB1*-QFbis_57700 and *attB2*-QRter_57720 primers. The corresponding entry vector was recombined with the pAM-PAT-GW destination vector [54] to generate the complementation binary plasmid. amiRNAs for *At3g57710* and *At3g57720*, alone and in combination (Table S2), were designed in the Col-0 background using the WMD online tool (http://wmd.weigelworld.org/) and the pRS300 vector as first PCR template.

### Kinase activity assays

For construction, expression and purification of His-, GST- and MBP-tagged proteins in *E. coli*, *RKS1* genes from Col-0 or Kas-1 were amplified from Arabidopsis genomic DNA using the *attB1F*-710 and *attB2R*-710 primers (Table S2). For production of RKS1 proteins in *E. coli*, three different tags were used to rule out the possibility that a given tag (and/or the protocol used for protein purification) may interfere with enzymatic activity. 3xHA-, GST-6xHis- and 6xHis-MBP-tagged proteins were generated by recombination of the corresponding pENTR constructs with pTH19-GW- 6His-3HA vector, pGEX-GST-GW-6His or pDEST-6-His-MBP-GW destination vectors, respectively. These constructs were introduced into the *Rosetta* strain of *E. coli*. Protein expression was induced and RKS1 proteins purified following standard protocols. For production of RKS1 proteins in *N. benthamiana*, corresponding pENTR constructs were recombined with pBin19 -35S-GW-3xFlag-TEV-3xHA and introduced into the C58C1 strain of *Agrobacterium tumefaciens*. Transient expression in *N. benthamiana* leaves was performed as described previously [53]. Soluble proteins were extracted according to Oh *et al.* [55] and RKS1 proteins affinity purified using anti-HA affinity matrix (Roche). Immunoprecipitated proteins were washed four times with extraction buffer and treated with TEV protease (Invitrogen) (0.1 U/µl) for 2 h at 16°C. For enzymatic assays, RKS1 autophosphorylation activity was tested as described [56]. In these assays, the kinase domain of the Arabidopsis BRI1 protein was used as a positive control and displayed kinase activity [55]. In the same conditions, the phosphorylation of histone (Sigma), casein (Sigma) or bovine Myelin Basic Protein (MBP, Upstate biotechnology, USA) as substrate was tested. Finally, in-gel kinase assays using casein, histone and MBP as substrate were performed following previously described protocols [19]–[20].

### RNA isolation and Q-RT-PCR

Leaves from RNA extraction and Q-RT-PCR analysis were performed as described [53] using leaves from healthy plants or inoculated with the *Xcc*568 strain (28 hpi) [52]. A gene (At2g28390, SAND family) whose expression has been shown to be extremely stable under different physiological conditions [57] was used as a control. Average ΔCp was calculated from 4 independent experiments with 3 individual plants (3 leaves/plant). Data are expressed as fold induction of each point as compared to the wild type. RNA isolation from Arabidopsis seeds imbibed for 16 h was performed using a protocol adapted from http://cotton genome center.ucdavis.edu/protocols/RNA.

In order to study the relationship between disease index and expression level of *RKS1* (or *At3g57720*) in different transgenic lines and natural accessions, linear and non-linear regressions were fitted using the ‘lm’ and ‘nls’ functions implemented in the *R* environment, respectively [58]. Model selection was based on a difference of three points in Akaike's information criterion (AIC), Bayesian information criterion (BIC) and AICc.

### RACE assays

The 5′ and the 3′ ends of *RKS1* mRNA accumulating in leaves were determined using the GeneRacer™ kit (Invitrogen, France) according to manufacturers' instructions. RNA from Col-0 and Kas-1 healthy and infected leaves (28 hpi) was prepared [53]. Products of three consecutive PCRs using primers shown in Table S2 were cloned in pGEM-T Easy vector (Promega Corporation) and sequenced.

### Statistical analysis

For QTL mapping, data were analyzed for each block and each experiment. Adjusted means of disease scores (LSmeans) of RILs in blocks were estimated from variance analysis (ANOVA). Broad sense heritabilities (*H* ^2^) were estimated from the mean square (MS) of ANOVA using the formula adapted from Gallais [59]. Variance analysis of *in planta* bacterial growth data was performed using PROC GLM of SAS with random effects. QTL analysis was done using the R-qtl package [60].

For GWA mapping, the following general linear model was used to analyze disease index (GLM procedure in SAS9.1, SAS Institute Inc., Cary, North Carolina, USA):
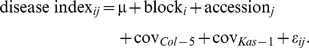
Where ‘μ’ is the overall mean; ‘block’ accounts for differences among the four experimental blocks; ‘accession’ corresponds to the 384 natural accessions; cov*_Col-5_* and cov*_Kas-1_* are covariates accounting for mini-greenhouse effects; and ‘ε’ is the residual term. Normality of the residuals was not improved by transformation of the data. Least-square mean (LSmean) was obtained for each natural accession and was subsequently used for GWA mapping analyses. All the 384 accessions have been genotyped for 214,051 SNPs. In order to fine-map genomic regions associated with natural disease index variation, we ran a Wilcoxon rank-sum test [61] and a mixed-model approach implemented in the software EMMAX (Efficient Mixed-Model Association eXpedited [62]. The latter model includes a genetic kinship matrix as a covariate to control for population structure. The percentage of quantitative disease resistance explained by the three allelic groups detected by nested GWA mapping was estimated in two polymorphic natural populations MIB and TOU (Table S7).

### Sequencing of natural accessions and molecular evolutionary genetics analysis

Ninety-five natural accessions already sequenced for 876 short fragments of ∼500 bp [63] were sequenced for a 5,111 bp region centered on *At3g57710* (i.e. *RKS1*), using five pairs of primers described in Table S2. Genbank accession numbers for the 5,111 bp sequences produced in this study are KF363545–KF363829.

The average number of nucleotide substitutions per site between the two intergenic haplotypes and the Tajima test were computed across the 5,085 bp region (window size = 100 bp, step size = 10 bp; window size = 500 bp, step = 50 bp, respectively) [64]. Note that nucleotide diversity and Tajima's *D* were estimated using nucleotide and insertion/deletion (indel) polymorphism data, the latter coded as single characters.

## Supporting Information

Figure S1 Natural variation of quantitative resistance to *Xanthomonas campestris* pv. *campestris* strain 568 among 23 *Arabidopsis thaliana* accessions. Disease symptoms were assessed 7 days post-inoculation with a bacterial suspension adjusted to 2×10^8^ cfu/mL. Means and standard errors were calculated from 4–26 plants.
(PDF)

Figure S2 Confirmation of *QRX3* in heterogeneous inbred family lines. (A) HIF900 and HIF903 still segregate for the *R30025* and *nga6* interval, including the *QRX3* interval. (B) Comparison of disease scores of plants of Col-5 HIF900 and HIF903 alleles *versus* the Kas-1 alleles of the same HIFs, 7 days post- inoculation.
(PDF)

Figure S3 Molecular characterization of the T-DNA knockout *rks1-1* Arabidopsis mutant, HIF lines and of corresponding plants complemented with a genomic *RKS1* transgene. (A) Structure of the *rks1-1* mutant and the construct used for complementation. *RKS1* is constituted by one exon (grey box) where the A in the start codon, ATG, is the first numbered nucleotide. The T-DNA insertion occurs at position -135 in *rks1-1*, as demonstrated by sequencing of the T-DNA borders and of their flanking region. (B) Expression analysis of *RKS1* and *At3g57720* genes in healthy (grey box) and infected (black box) leaves of wild-type (Col-0), mutant (*rks1-1*) and complemented lines (#9, #E9, #F9). (C) Expression analysis of *RKS1* and *At3g57720* genes in healthy (grey box) and infected (black box) leaves of wild-type (Col-0, Kas-1), HIF lines (HIF685, susceptible, HIF 1011, resistant) and lines transformed with the *RKS1* transgene (for HIF685, #105, #106; for HIF1011, #107, #110).
(PDF)

Figure S4 Phenotypic and molecular analysis of amiRNA lines for *At3g57720* and *RKS1+*At3g57720 genes in the Col-0 background. (A and B) Disease symptoms were observed on leaves of wild-type plants and of amiRNA lines 10 days post-inoculation. Time course evaluation of disease index after inoculation with *Xcc568* under the same conditions. (C) *RKS1* and *At3g57720* gene expression analysis in infected leaves of *RKS1* (lines #23 and #24), *At3g57720* (lines #A6 and #E10) and *RKS1+At3g57720* (lines #7 and #39) amiRNA lines.
(PDF)

Figure S5 Phenotypic and molecular analysis of resistant lines transformed with the susceptible allele of *RKS1*. Disease symptoms were observed on leaves of wild-type plants and transgenic lines, 10 days post-inoculation. Time course evaluation of disease index after inoculation with *Xcc568* under the same conditions. (A) Transformation of Col-0 with the susceptible allele of *RKS1*. (B) Transformation of the resistant HIF 1011 with the susceptible allele of *RKS1*.
(PDF)

Figure S6 RKS1 presents all features of an atypical kinase. (A) Alignment of the kinase domains of RKS1 from Col-0 and those of well characterized protein kinases with demonstrated catalytic activity. The major domains in the kinase catalytic core of kinase proteins, which are essential for catalysis, are boxed in red. The polymorphic residue between RKS1 from Col-0 and RKS1 from Kas-1 is indicated by an asterisk. Numbering corresponds to the amino acid sequence. Every tenth residue in RKS1 is marked by !. Percentage of sequence identity is represented by colour intensity of blue boxes. RKS1 (AEE79688), BRI1 (AEE87069), CTR1 (AED90648), IRAK4 (NP_001107654), LYK3 (AAQ73159), MPK3 (AEE78054), OsWAK1 (AAG61114), Pto (AAZ15325), WRKS1 (ACF33187), Xa21 (AAC49123). (B and C) Negative results obtained in autophosphorylation (B) and MBP phosphorylation (C) assays with 3xFlag-TEV-3xHA-tagged RKS1 from the Col-0 and the Kas-1 ecotypes transiently expressed in *N. benthamiana* are shown as an example (see section Materials and Methods). In these assays, the kinase domain of Arabidopsis BRI1 (BRI1-KD) [56] was used as a positive control. Molecular mass markers in kilodaltons are indicated on the right.
(PDF)

Figure S7 Schematic illustration of the 5′-end and 3′-end products of *RKS1* transcripts identified by respectively 5′ and 3′RACE. (A) Nucleotide sequence of *RKS1* from Col-0. The ATG and TAG codons are underlined and bold. The SNP present in the coding region of *RKS1* between the Col-0 and Kas-1 alleles is boxed. The primer specific of *RKS1* (reverse and forward) used to amplify 5′ and 3′ ends is underlined. In blue, the location of *RKS1* 5′ ends of Col-0 leaves, in pink the location of *RKS1* 5′ ends of Kas-1 leaves. In red, the location of *RKS1* 3′ ends of Kas-1 leaves; in green of Col-0 leaves. (B) Location and frequency (number of clones) of *RKS1* 5′ ends and *RKS1* 3′ ends products.
(PDF)

Figure S8 Whole-genome scan of 214,051 SNPs for association with disease index at 5 dpi, 7 dpi and 10 dpi across 381 accessions, using either the Wilcoxon model or the EMMAX method. The y-axis indicates the –log^10^ *p*-values using the Wilcoxon model or the EMMAX method. DI stands for disease index. MARF = 0.05.
(PDF)

Figure S9 Quantile-Quantile plot of p-values (raw and negative logarithm) in genome-wide scans for disease index at 10 dpi. (A) All accessions (n = 381). (B) Accessions of the R allelic group SNP-3-21386192-C (n = 279). (C) Accessions of the S allelic group SNP-3-21386192-T (n = 102). The different curves correspond to different analyses of GWA mapping. Dashed black line: expected; dashed red line: Wilcoxon; dashed blue line: EMMAX.
(PDF)

Figure S10 Zoom on the association peaks around *RKS1*. Moccasin colored area indicates the eight putative kinases (including *RKS1*) region.
(PDF)

Figure S11 Whole-genome scan of 214,051 SNPs for association with disease index at 10 dpi across (A) the accessions of the S allelic group SNP-3-21386192-T (n = 102) and (B) the accessions of the R allelic group SNP-3-21386192-C (n = 279), using either the Wilcoxon model or the EMMAX method. The *y*-axis indicates the –log^10^ *p*-values using the Wilcoxon model or the EMMAX method. MARF = 0.05.
(PDF)

Figure S12 Alignment of the 12 different RKS1 protein sequences from *A. thaliana* and putative RKS1 orthologous from *Arabidopsis lyrata* and *Brassica rapa* subsp. *pekinensis*. The major domains in the kinase catalytic core of kinase proteins described in Figure S6 are underlined in red. Numbering corresponds to the amino acid sequence. Every tenth residue in RKS1 is marked by *. *A. lyrata* NCBI Reference Sequence: XM_002878122.1. *Brassica rapa* subsp. *pekinensis* Sequence ID: gb|AC232509.1|.
(PDF)

Figure S13 Relationship between disease index and expression levels of *RKS1*. (A) *RKS1* total mRNA expression (*RKS1-L+RKS1-S* transcripts) considering all the accessions. (B) *RKS1-L* expression considering all the accessions. (C) *RKS1-L* expression considering all the accessions minus the accessions with a stop codon. (D) *RKS1-L* expression considering all the accessions minus the accessions with one of the two independent susceptible alleles within the R intergenic haplotype. Solid, dashed and dotted lines indicate negatively linear, exponentially decreasing and logarithmically decreasing functions, respectively. Parameters for each linear and non-linear model are given in Table S9.
(PDF)

Figure S14 Relationship between disease index and expression levels of *At3g57720*. No linear or non-linear relationship between disease index and relative gene expression of *At3g57720* was detected. 1) linear model (disease∼intercept+a*expression): intercept = 0.336 (*P* = 0.0525), a = 0.661 (*P* = 0.1398). 2) exponential function (disease∼Ae^-k*expression^): A = 0.388 (*P* = 0.0017), k = −1.069 (*P* = 0.1529). 3) logarithmic function (disease∼b*log(expression)+c): b = −0.242 (*P* = 0.0990), c = −0.836 (*P* = 1.95×10^−6^).
(PDF)

Figure S15 Whole-genome scan of 214,051 SNPs for association with (A) the relative gene expression of *RKS1-L+S* (i.e. total mRNA) or (B) the relative gene expression of *RKS1-L* (n = 88). The *y*-axis indicates the –log^10^ *p*-values using the Wilcoxon model or the EMMAX method. MARF = 0.05.
(PDF)

Figure S16 Geographic distribution of *RKS1* polymorphisms. (A) Geographic distribution of the two highly divergent haplotypes located in the intergenic region between *RKS1* and *At3g57720* and at the beginning of *RKS1*, using one of the 35 SNPs in complete LD, i.e. a SNP at position 21,387,232 on chromosome 3. (B) Geographic distribution of two S susceptible alleles embedded in the R intergenic haplogroup (n = 476). The geographic distribution of the accessions with a stop codon at the beginning of *RKS1* was mapped using one of the 214,051 SNPs that is in complete LD with the stop codon; i.e. a SNP at position 21,388,948 on chromosome 3 (position 4129 in Table S8). The geographic distribution of the accessions with the additional S susceptible allele was mapped using accessions with both a ‘G’ base at position 21,388,849 on chromosome 3 (i.e. position 4030 in Table S8) and a ‘T’ base at position 21,389,085 on chromosome 3 (i.e. position 4266 in Table S8). All maps have been based on 948 natural accessions with accurate GPS coordinates and genotyped for 214,051 SNPs [65] and generated with the *R* packages ‘maptools’ and ‘plotrix’. The size of the pies depends of the number of accessions genotyped for 214,051 SNPs in the sites of collection.
(PDF)

Table S1 Trait heritability and QTL detection.
(PDF)

Table S2 List of PCR-based marker and oligonucleotide sequences.
(PDF)

Table S3 Genotypes and phenotypes of 10 lines with the closest recombination events flanking QRX3. Line phenotypes in response to inoculation with *Xcc568* were evaluated 6 and 9 days post-inoculation (R = resistant; S = susceptible; I = intermediate phenotype). Line genotypes: B: alleles of the susceptible parent Kas-1; A: alleles of the resistant parent Col-5; H: heterozygous. Primers are listed in Table S2.
(PDF)

Table S4 Mutants identified in the QRX3 locus and their phenotype in response to inoculation to *Xcc568*.
(PDF)

Table S5 Fitting of one linear model and two non-linear models on the relationship between disease index and expression levels of *RKS1* using various transgenic lines.
(PDF)

Table S6 Model selection among one linear model and two non-linear models on the relationship between disease index and expression levels of *RKS1* using various transgenic lines.
(PDF)

Table S7 Disease index for 384 accessions used in this study.
(PDF)

Table S8 Sequence polymorphisms of the 5,111 bp region centered on *RKS1*.
(PDF)

Table S9 Fitting of one linear model and two non-linear models on the relationship between disease index and expression levels of *RKS1* in natural accessions.
(PDF)

Table S10 Model selection among one linear model and two non-linear models on the relationship between disease index and expression levels of *RKS1* in natural accessions.
(PDF)
