# Supplementary material for: An Atypical Kinase under Balancing Selection Confers Broad-Spectrum Disease Resistance in Arabidopsis
Source: PLoS Genet. 2013 Sep 12;9(9):e1003766. doi: 10.1371/journal.pgen.1003766 (PMC3772041; doi:10.1371/journal.pgen.1003766)
Supplement: Figure S1 — Natural variation of quantitative resistance to Xanthomonas campestris pv. campestris strain 568 among 23 Arabidopsis thaliana accessions. Disease symptoms were assessed 7 days post-inoculation with a bacterial suspension adjusted to 2×10^8^ cfu/mL. Means and standard errors were calculated from 4–26 plants. (PDF) [file pgen.1003766.s001.pdf]

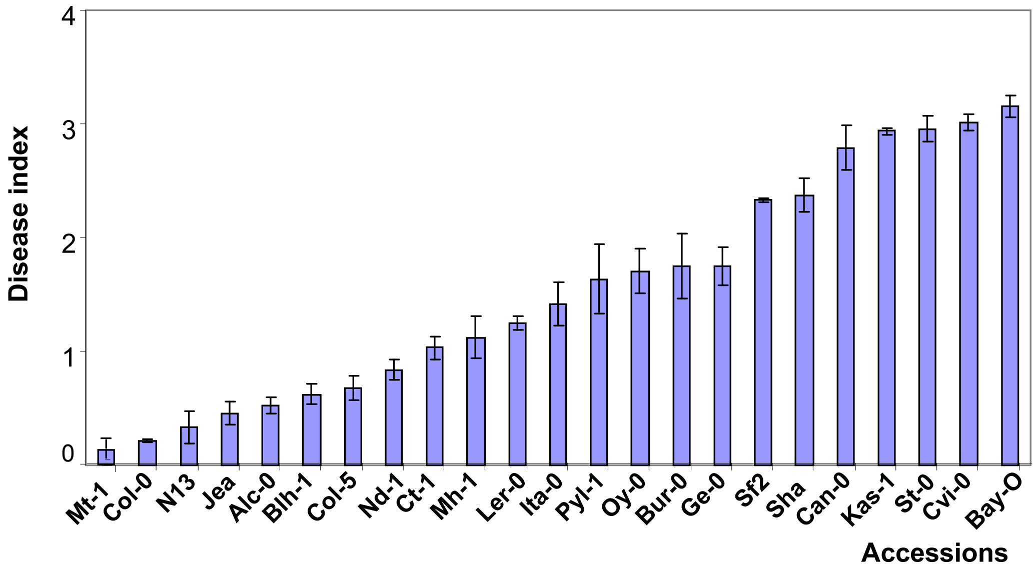

Figure S1. Natural variation of quantitative resistance to *Xanthomonas campestris* pv. *campestris* strain 568 among 23 *Arabidopsis thaliana* accessions. Disease symptoms were assessed 7 days post-inoculation with a bacterial suspension adjusted to $2x10^8$ cfu/mL. Means and standard errors were calculated from 4-26 plants.
