# Supplementary material for: An Atypical Kinase under Balancing Selection Confers Broad-Spectrum Disease Resistance in Arabidopsis
Source: PLoS Genet. 2013 Sep 12;9(9):e1003766. doi: 10.1371/journal.pgen.1003766 (PMC3772041; doi:10.1371/journal.pgen.1003766)
Supplement: Figure S2 — Confirmation of QRX3 in heterogeneous inbred family lines. (A) HIF900 and HIF903 still segregate for the R30025 and nga6 interval, including the QRX3 interval. (B) Comparison of disease scores of plants of Col-5 HIF900 and HIF903 alleles versus the Kas-1 alleles of the same HIFs, 7 days post- inoculation. (PDF) [file pgen.1003766.s002.pdf]

A

HIF / marker	T04109	r30025	nga6
900	B	H	H
903	A	H	H

B

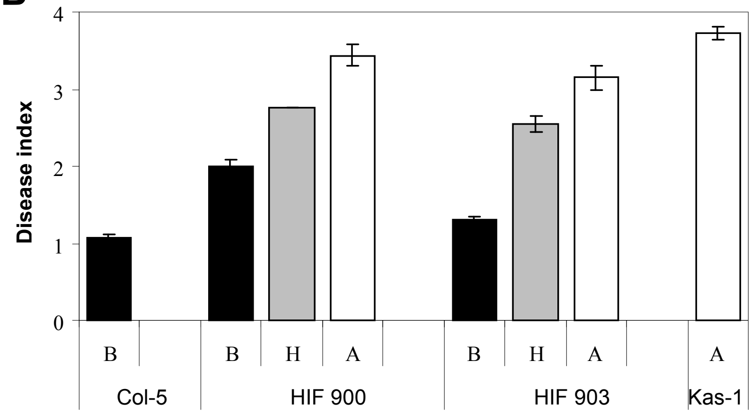

Figure S2. **Confirmation of *QRX3* in heterogeneous inbred family lines.** (A) HIF900 and HIF903 still segregate for the R30025 and nga6 interval, including the *QRX3* interval. (B) Comparison of disease scores of plants of Col-5 HIF900 and HIF903 alleles *versus* the Kas-1 alleles of the same HIFs, 7 days post- inoculation.
