# Supplementary material for: An Atypical Kinase under Balancing Selection Confers Broad-Spectrum Disease Resistance in Arabidopsis
Source: PLoS Genet. 2013 Sep 12;9(9):e1003766. doi: 10.1371/journal.pgen.1003766 (PMC3772041; doi:10.1371/journal.pgen.1003766)
Supplement: Figure S3 — Molecular characterization of the T-DNA knockout rks1-1 Arabidopsis mutant, HIF lines and of corresponding plants complemented with a genomic RKS1 transgene. (A) Structure of the rks1-1 mutant and the construct used for complementation. RKS1 is constituted by one exon (grey box) where the A in the start codon, ATG, is the first numbered nucleotide. The T-DNA insertion occurs at position -135 in rks1-1, as demonstrated by sequencing of the T-DNA borders and of their flanking region. (B) Expression analysis of RKS1 and At3g57720 genes in healthy (grey box) and infected (black box) leaves of wild-type (Col-0), mutant (rks1-1) and complemented lines (#9, #E9, #F9). (C) Expression analysis of RKS1 and At3g57720 genes in healthy (grey box) and infected (black box) leaves of wild-type (Col-0, Kas-1), HIF lines (HIF685, susceptible, HIF 1011, resistant) and lines transformed with the RKS1 transgene (for HIF685, #105, #106; for HIF1011, #107, #110). (PDF) [file pgen.1003766.s003.pdf]

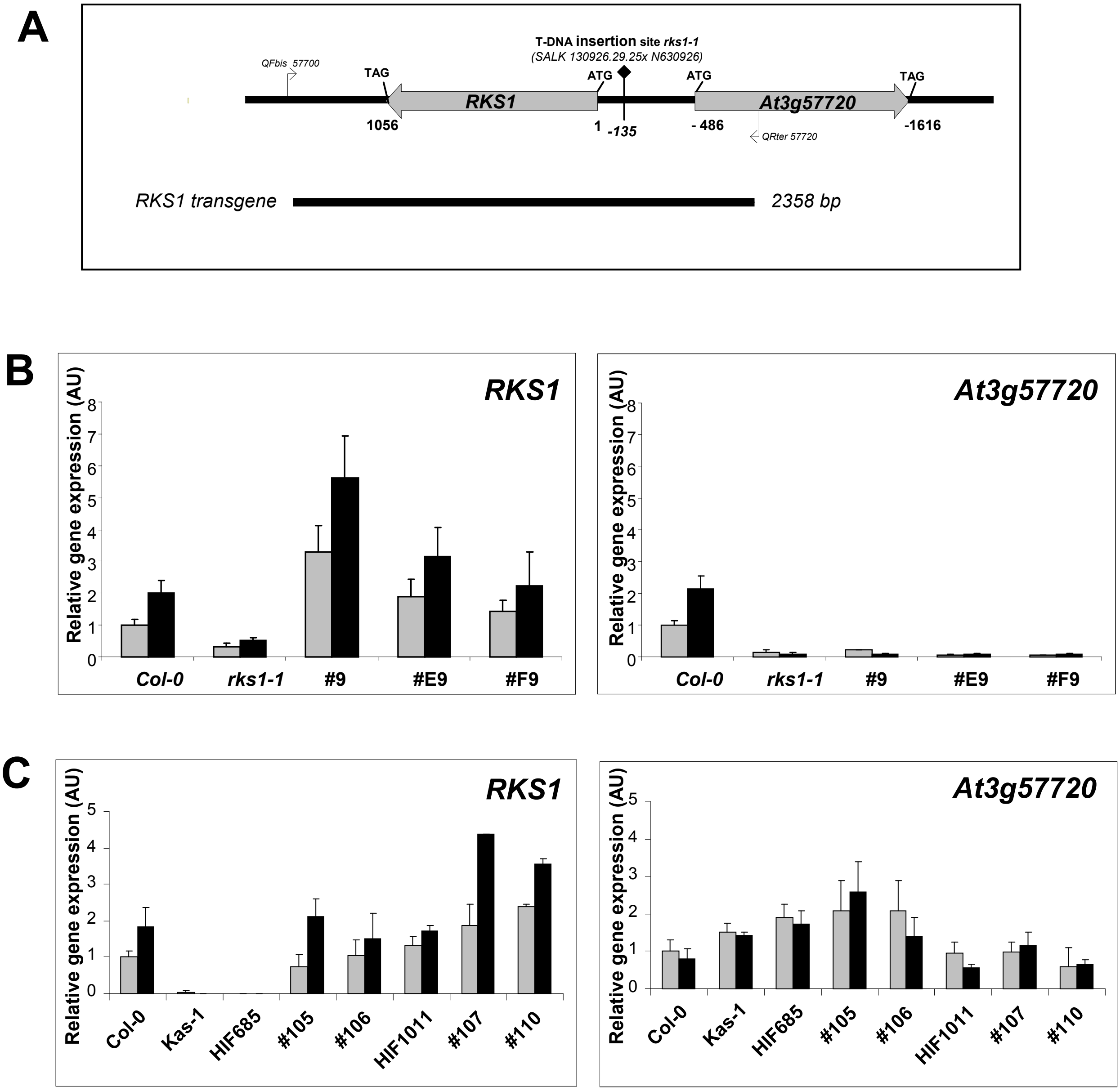

Figure S3. Molecular characterization of the T-DNA knockout *rks1-1* Arabidopsis mutant, HIF lines and of corresponding plants complemented with a genomic *RKS1* transgene. (A) Structure of the *rks1-1* mutant and the construct used for complementation. *RKS1* is constituted by one exon (grey box) where the A in the start codon, ATG, is the first numbered nucleotide. The T-DNA insertion occurs at position -135 in *rks1-1*, as demonstrated by sequencing of the T-DNA borders and of their flanking region. (B) Expression analysis of *RKS1* and *At3g57720* genes in healthy (grey box) and infected (black box) leaves of wild-type (Col-0), mutant (*rks1-1*) and complemented lines (#9, #E9, #F9). (C) Expression analysis of *RKS1* and *At3g57720* genes in healthy (grey box) and infected (black box) leaves of wild-type (Col-0, Kas-1), HIF lines (HIF685, susceptible, HIF 1011, resistant) and lines transformed with the *RKS1* transgene (for HIF685, #105, #106; for HIF1011, #107, #110).
