# Supplementary material for: An Atypical Kinase under Balancing Selection Confers Broad-Spectrum Disease Resistance in Arabidopsis
Source: PLoS Genet. 2013 Sep 12;9(9):e1003766. doi: 10.1371/journal.pgen.1003766 (PMC3772041; doi:10.1371/journal.pgen.1003766)
Supplement: Figure S4 — Phenotypic and molecular analysis of amiRNA lines for At3g57720 and RKS1+At3g57720 genes in the Col-0 background. (A and B) Disease symptoms were observed on leaves of wild-type plants and of amiRNA lines 10 days post-inoculation. Time course evaluation of disease index after inoculation with Xcc568 under the same conditions. (C) RKS1 and At3g57720 gene expression analysis in infected leaves of RKS1 (lines #23 and #24), At3g57720 (lines #A6 and #E10) and RKS1+At3g57720 (lines #7 and #39) amiRNA lines. (PDF) [file pgen.1003766.s004.pdf]

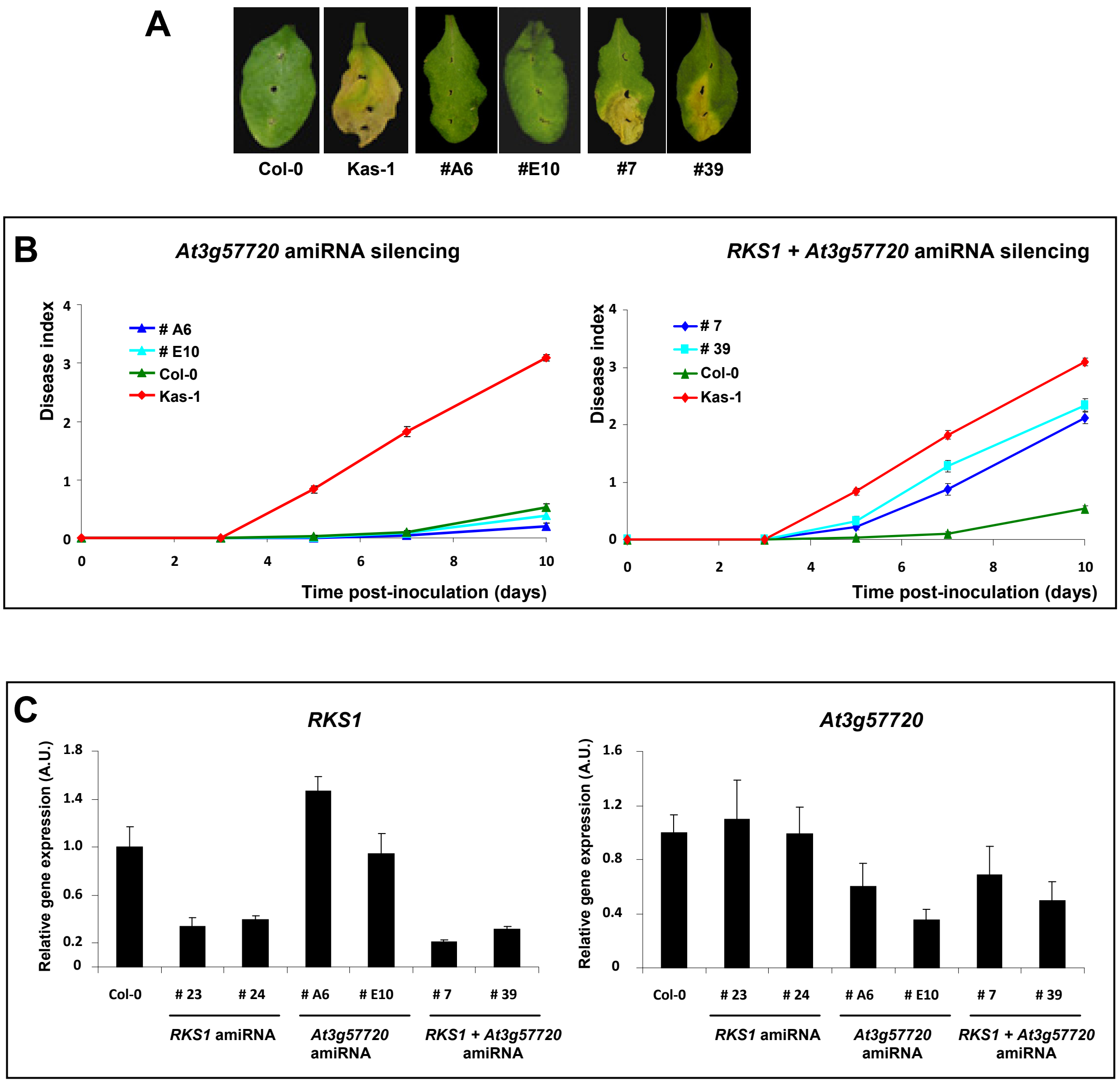


**Figure S4 . Phenotypic and molecular analysis of amiRNA lines for *At3g57720* and *RKS1*+ At3g57720 genes in the Col-0 background.** (A and B) Disease symptoms were observed on leaves of wild-type plants and of amiRNA lines 10 days post-inoculation. Time course evaluation of disease index after inoculation with *Xcc568* under the same conditions. (C ) *RKS1* and *At3g57720* gene expression analysis in infected leaves of *RKS1* (lines #23 and #24), *At3g57720* (lines #A6 and #E10) and *RKS1+At3g57720* (lines #7 and #39) amiRNA lines.
