# Supplementary material for: An Atypical Kinase under Balancing Selection Confers Broad-Spectrum Disease Resistance in Arabidopsis
Source: PLoS Genet. 2013 Sep 12;9(9):e1003766. doi: 10.1371/journal.pgen.1003766 (PMC3772041; doi:10.1371/journal.pgen.1003766)
Supplement: Figure S5 — Phenotypic and molecular analysis of resistant lines transformed with the susceptible allele of RKS1. Disease symptoms were observed on leaves of wild-type plants and transgenic lines, 10 days post-inoculation. Time course evaluation of disease index after inoculation with Xcc568 under the same conditions. (A) Transformation of Col-0 with the susceptible allele of RKS1. (B) Transformation of the resistant HIF 1011 with the susceptible allele of RKS1. (PDF) [file pgen.1003766.s005.pdf]

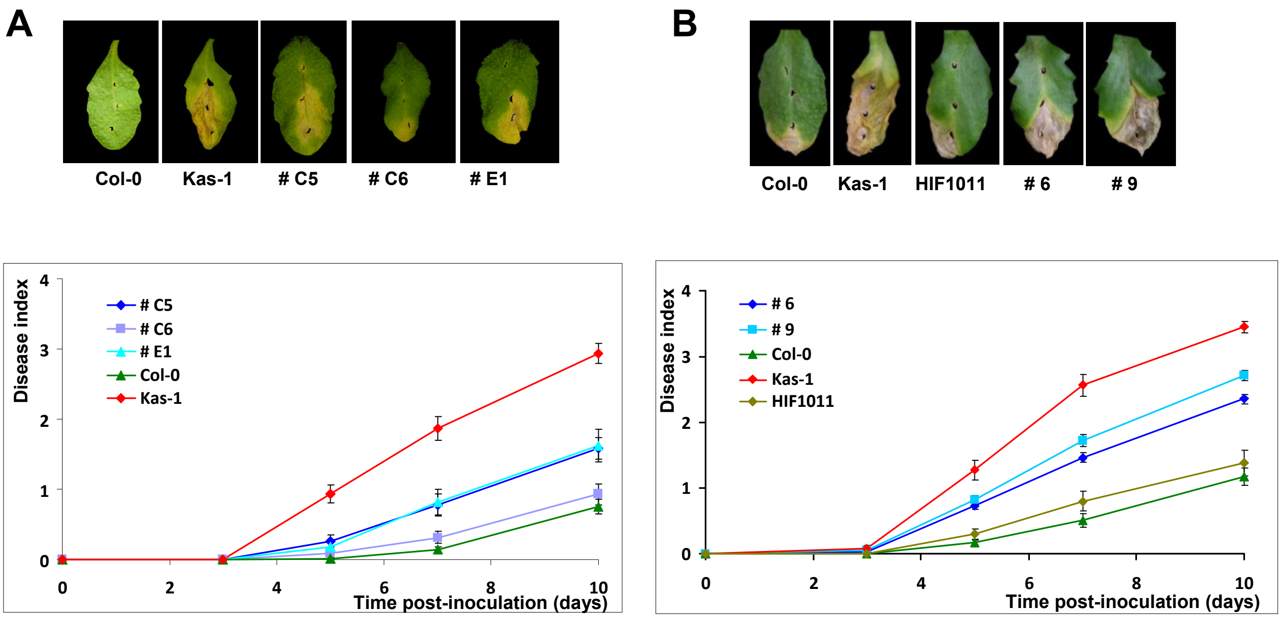

Figure S5 . Phenotypic and molecular analysis of resistant lines transformed with the susceptible allele of *RKS1*. Disease symptoms were observed on leaves of wild-type plants and transgenic lines, 10 days post-inoculation. Time course evaluation of disease index after inoculation with *Xcc568* under the same conditions. (A) Transformation of Col-0 with the susceptible allele of *RKS1*. (B) Transformation of the resistant HIF 1011 with the susceptible allele of *RKS1*.
