# Supplementary material for: An Atypical Kinase under Balancing Selection Confers Broad-Spectrum Disease Resistance in Arabidopsis
Source: PLoS Genet. 2013 Sep 12;9(9):e1003766. doi: 10.1371/journal.pgen.1003766 (PMC3772041; doi:10.1371/journal.pgen.1003766)
Supplement: Figure S6 — RKS1 presents all features of an atypical kinase. (A) Alignment of the kinase domains of RKS1 from Col-0 and those of well characterized protein kinases with demonstrated catalytic activity. The major domains in the kinase catalytic core of kinase proteins, which are essential for catalysis, are boxed in red. The polymorphic residue between RKS1 from Col-0 and RKS1 from Kas-1 is indicated by an asterisk. Numbering corresponds to the amino acid sequence. Every tenth residue in RKS1 is marked by !. Percentage of sequence identity is represented by colour intensity of blue boxes. RKS1 (AEE79688), BRI1 (AEE87069), CTR1 (AED90648), IRAK4 (NP_001107654), LYK3 (AAQ73159), MPK3 (AEE78054), OsWAK1 (AAG61114), Pto (AAZ15325), WRKS1 (ACF33187), Xa21 (AAC49123). (B and C) Negative results obtained in autophosphorylation (B) and MBP phosphorylation (C) assays with 3xFlag-TEV-3xHA-tagged RKS1 from the Col-0 and the Kas-1 ecotypes transiently expressed in N. benthamiana are shown as an example (see section Materials and Methods). In these assays, the kinase domain of Arabidopsis BRI1 (BRI1-KD) [56] was used as a positive control. Molecular mass markers in kilodaltons are indicated on the right. (PDF) [file pgen.1003766.s006.pdf]

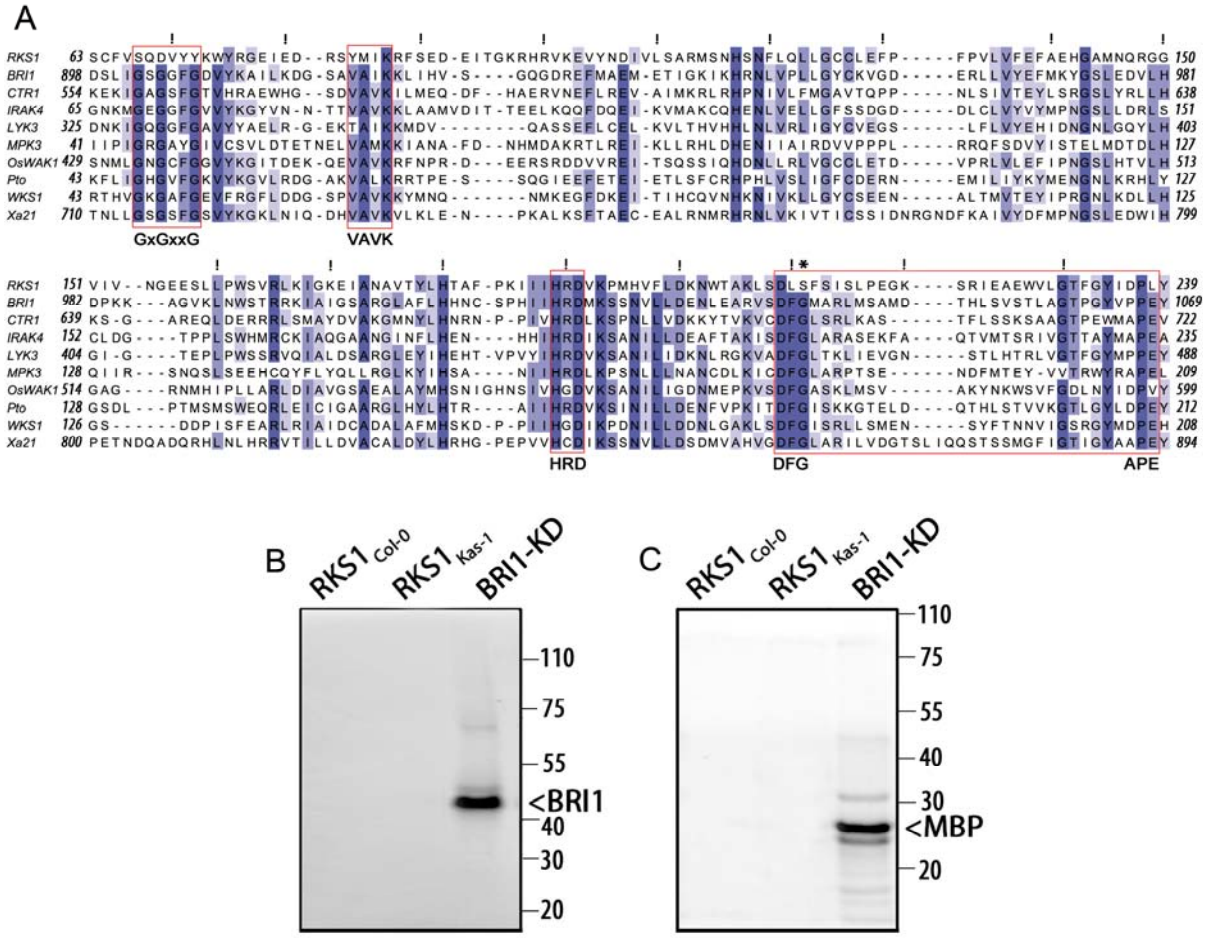


**Figure S6. RKS1 presents all features of an atypical kinase.**

(A) Alignment of the kinase domains of RKS1 from Col-0 and those of well characterized protein kinases with demonstrated catalytic activity. The major domains in the kinase catalytic core of kinase proteins, which are essential for catalysis, are boxed in red. The polymorphic residue between RKS1 from Col-0 and RKS1 from Kas-1 is indicated by an asterisk. Numbering corresponds to the amino acid sequence. Every tenth residue in RKS1 is marked by !. Percentage of sequence identity is represented by colour intensity of blue boxes. RKS1 (AEE79688), BRI1 (AEE87069), CTR1 (AED90648), IRAK4 (NP_001107654), LYK3 (AAQ73159), MPK3 (AEE78054), OsWAK1 (AAG61114), Pto (AAZ15325), WRKS1 (ACF33187), Xa21 (AAC49123). (B and C) Negative results obtained in autophosphorylation (B) and MBP phosphorylation (C) assays with 3xFlag-TEV-3xHA-tagged RKS1 from the Col-0 and the Kas-1 ecotypes transiently expressed in *N. benthamiana* are shown as an example (see section Materials and Methods). In these assays, the kinase domain of Arabidopsis BRI1 (BRI1-KD) [56] was used as a positive control. Molecular mass markers in kilodaltons are indicated on the right.
