# Supplementary material for: An Atypical Kinase under Balancing Selection Confers Broad-Spectrum Disease Resistance in Arabidopsis
Source: PLoS Genet. 2013 Sep 12;9(9):e1003766. doi: 10.1371/journal.pgen.1003766 (PMC3772041; doi:10.1371/journal.pgen.1003766)
Supplement: Figure S7 — Schematic illustration of the 5′-end and 3′-end products of RKS1 transcripts identified by respectively 5′ and 3′RACE. (A) Nucleotide sequence of RKS1 from Col-0. The ATG and TAG codons are underlined and bold. The SNP present in the coding region of RKS1 between the Col-0 and Kas-1 alleles is boxed. The primer specific of RKS1 (reverse and forward) used to amplify 5′ and 3′ ends is underlined. In blue, the location of RKS1 5′ ends of Col-0 leaves, in pink the location of RKS1 5′ ends of Kas-1 leaves. In red, the location of RKS1 3′ ends of Kas-1 leaves; in green of Col-0 leaves. (B) Location and frequency (number of clones) of RKS1 5′ ends and RKS1 3′ ends products. (PDF) [file pgen.1003766.s007.pdf]

A

-171 ACCCAAGACT TTTTATTGCT TCTTCCTTTC TTATGACACA CCGTTTTCAA CCGAGAGTCG

-111 TTTGGGATCA GAAAAGAAAT GGCTTTTGAC ACTTCAGAAG CTTTTAGGTA AGAAAGGTGT

-51 CCTCTTTATT CATCTCGCCA ACAAAAGGTT GATACTTTGG TAAAGGAACT A**ATG**AAGAAG

RKS1 Start codon

+10 CAGTATCTGA AATCTGGATC TGGGACAAGG AAGGAAAAGG ACAAGGCGAA GAGGTGGTTC

+70 TTGGATAACG GAAGCATCTT CTTAAGAGAA CTTGTAGCTG ATTGTAACGG TAAATCCATT

+130 CCTATTCGCA GTTTTTCTCC TGAGCAGATA TTGAAAGCCA CCAACAATTT CGATTCTAGC

+190 TGTTTTGTCT CGCAAGATGT ATACTATAAA TGGTATAGAG GTGAAATTGA AGATAGATCT

+250 TACATGATCA AGAGATTCTC AGAGGATGAG ATTACAGGAA AACGACATAG AGTTAAAGAG

273F/298R

+310 GTTTACAACG ACATTGTCTT GTCTGCTCGG ATGAGTAATC ACAGTAACTT TCTTCAACTT

+370 TTAGGATGTT GTCTCGAGTT TCCTTTTCCG GTTCTTGTGT TTGAATTTGC AGAACATGGA

+430 GCTATGAATC AGCGAGGAGG TGTTATAGTT AATGGTGAAG AATCTTTATT GCCGTGGAGT

+490 GTACGGTTGA AGATTGGGAA AGAGATTGCT AATGCGGTGA CTTATCTTCA CACGGCATTC

+550 CCTAAGATCA TCATACATAG AGATGTTAAA CCGATGCATG TTTTCTTGGA CAAGAATTGG

+610 ACCGCAAAGT TGTCTGATTT ATCCTTCTCA ATATCTCTAC CTGAGGGAAA ATCGAGAATA

+670 GAAGCTGAAT GGGTTCTAGG AACATTTGGG TACATCGATC CATTATATCA TAAGACGTGT

+730 TTTGTGACTG AATATACAGA TGTCTACAGC TTTGGAATCT GTTTGCTGGT TATTATTACT

+790 GGTAAACCGG CTATCATGAC TATTTCTGAT GGAGATCTCC AAGGTATTCT TAGCTTAGTG

+850 AGAGAGTTAT GTGAGAATGG GAAGCTTGAT GAAGTGATAG ATCCGAGGTT GATGAAAGAT

+910 ATCACAAGTG GTCAAAGGTT GCAAGTGGAA GCATGTGTGG TGCTGGCTTT GAGATGCTGT

+970 AAGGAGAGAG ATGAAGACAG ACCAAAGATG ATCCAAGTAG CTAAAGAACT CAAGCAGATT

+1030 GAAGCATCAT TGAAAAATTC TAGC**TAG**AGA TCCGCAAGGA ACTTTTTGGA TATTGAGATA

RKS1 Stop codon

+1090 TCTTCGCGGA GTAGGCATCA CAAGAACATG ACTGAAGATA GGAGCATCTT TGGTGCCCAA

+1150 AAGAAGAGGT ATCAGTTGCT TGGACTATAT AAATCTAAGT AAACAATTCT TGCATTGTGT

+1210 AGCAAGGAAA CTCAGATTCT TACATACAAC ATATTTAATA AAAGCTCGAT CAAAAACAAG

+1270 ATACAGTGAT TGCAATTTTC ACTACTAAGC CTGTATAATA GATAGACCTG TTTCAAGTCG

+1330 CATTAGAAAA GGTTGCATGG **TCA**TATATGT TTCTCTATCT TCTTAAGCTC TTTTGCAACG

Stop codon At3g57700

B

RACE 5'										
**Accession**	**Number of colonies**	**Base position of 5'end of *RKS1* transcripts relative to the position of the "A" (+1) in the start codon ATG**								
		**-129**	**-113**	**- 110**	**- 108**	**- 105**	**-102**	**-99**	**-10**	**-1**
Col-0	11	1	1	4	1			2		2
Kas-1	10				1	1	1		3	

RACE 3'							
**Accession**	**Number of colonies**	**Base position of 3'end of *RKS1* transcripts relative to the position of the "A" (+1) in the start codon ATG**					
		**+366**	**+370**	**+1268**	**+1293**	**+1330**	**+1334**
Col-0	10			3	2	5	
Kas-1	12	5	4	1			2

Figure S7. Schematic illustration of the 5'-end and 3'-end products of *RKS1* transcripts identified by respectively 5' and 3'RACE. (A) Nucleotide sequence of *RKS1* from Col-0. The ATG and TAG codons are underlined and bold. The SNP present in the coding region of *RKS1* between the Col-0 and Kas-1 alleles is boxed. The primer specific of *RKS1* (reverse and forward) used to amplify 5' and 3' ends is underlined. In blue, the location of *RKS1* 5' ends of Col-0 leaves, in pink the location of *RKS1* 5' ends of Kas-1 leaves. In red, the location of *RKS1* 3' ends of Kas-1 leaves; in green of Col-0 leaves. (B) Location and frequency (number of clones) of *RKS1* 5' ends and *RKS1* 3' ends products.
