# Supplementary material for: An Atypical Kinase under Balancing Selection Confers Broad-Spectrum Disease Resistance in Arabidopsis
Source: PLoS Genet. 2013 Sep 12;9(9):e1003766. doi: 10.1371/journal.pgen.1003766 (PMC3772041; doi:10.1371/journal.pgen.1003766)
Supplement: Figure S8 — Whole-genome scan of 214,051 SNPs for association with disease index at 5 dpi, 7 dpi and 10 dpi across 381 accessions, using either the Wilcoxon model or the EMMAX method. The y-axis indicates the –log10 p-values using the Wilcoxon model or the EMMAX method. DI stands for disease index. MARF = 0.05. (PDF) [file pgen.1003766.s008.pdf]

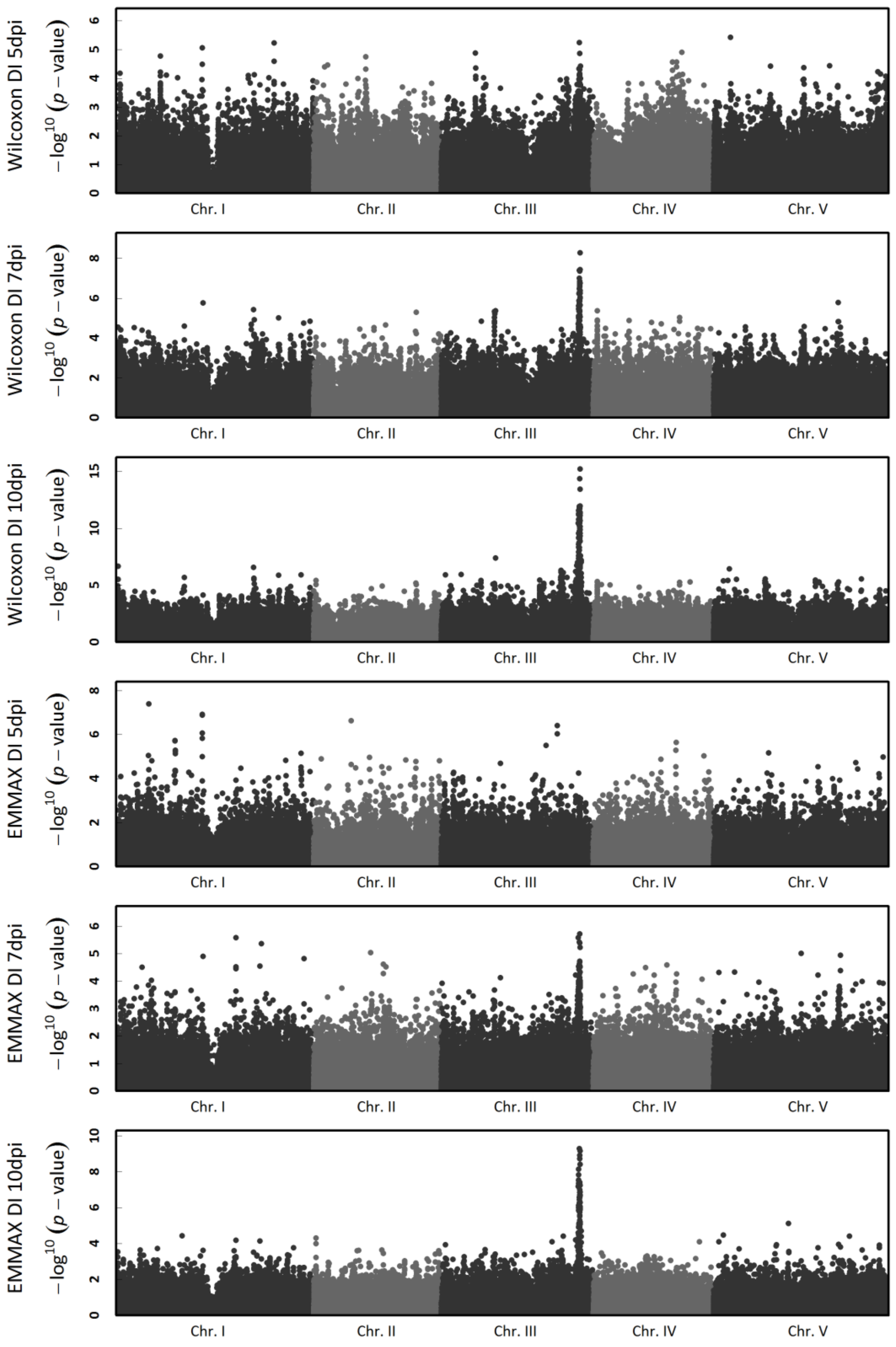

Figure S8. Whole-genome scan of 214,051 SNPs for association with disease index at 5dpi, 7 dpi and 10dpi across 381 accessions, using either the Wilcoxon model or the EMMAX method. The *y*-axis indicates the $-\log^{10}$ *p*-values using the Wilcoxon model or the EMMAX method. DI stands for disease index. MARF = 0.05.
