# Supplementary material for: An Atypical Kinase under Balancing Selection Confers Broad-Spectrum Disease Resistance in Arabidopsis
Source: PLoS Genet. 2013 Sep 12;9(9):e1003766. doi: 10.1371/journal.pgen.1003766 (PMC3772041; doi:10.1371/journal.pgen.1003766)
Supplement: Figure S9 — Quantile-Quantile plot of p-values (raw and negative logarithm) in genome-wide scans for disease index at 10 dpi. (A) All accessions (n = 381). (B) Accessions of the R allelic group SNP-3-21386192-C (n = 279). (C) Accessions of the S allelic group SNP-3-21386192-T (n = 102). The different curves correspond to different analyses of GWA mapping. Dashed black line: expected; dashed red line: Wilcoxon; dashed blue line: EMMAX. (PDF) [file pgen.1003766.s009.pdf]

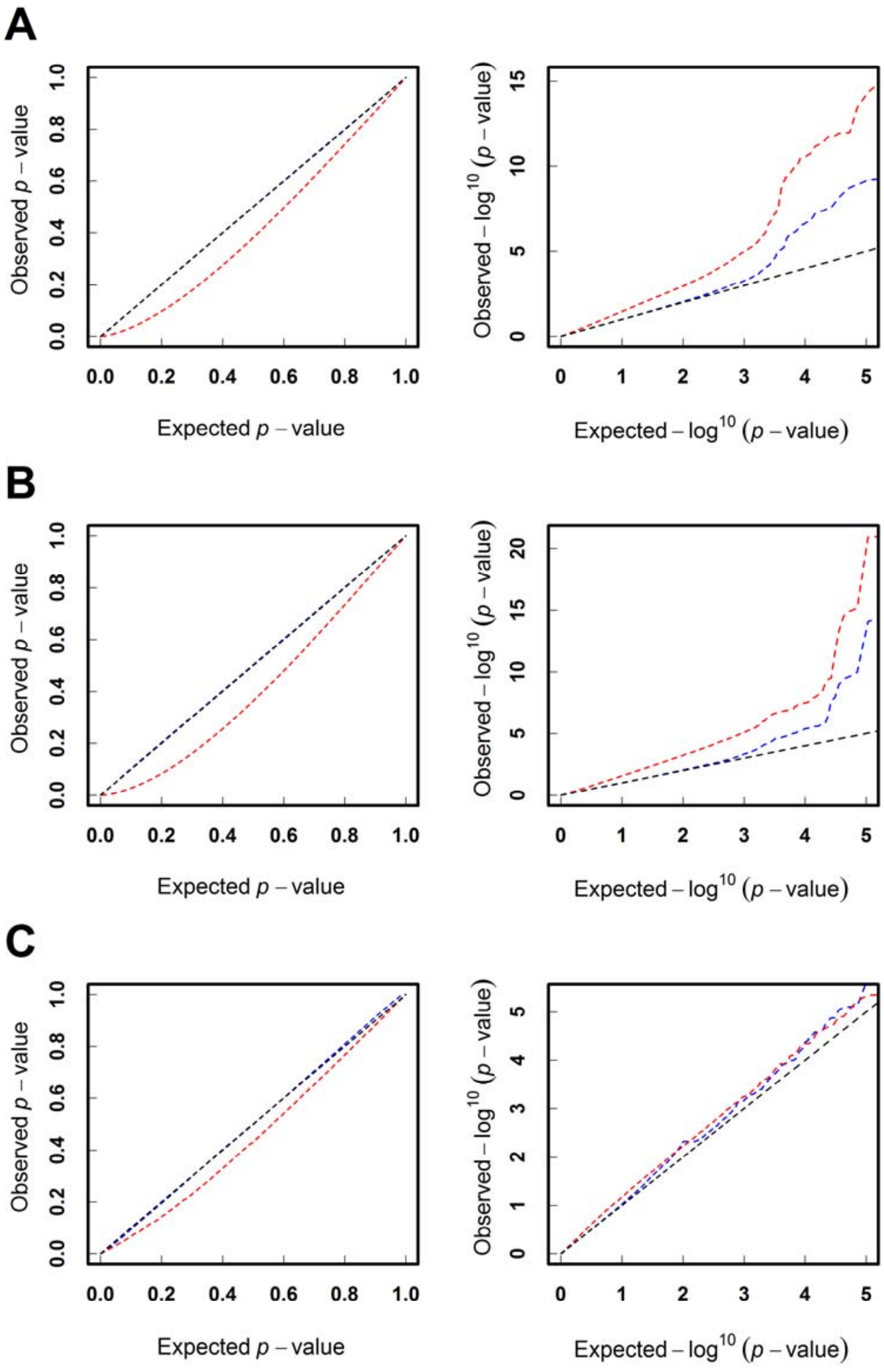

Figure S9. Quantile-Quantile plot of *p*-values (raw and negative logarithm) in genome-wide scans for disease index at 10dpi. (A) All accessions (n = 381). (B) Accessions of the R allelic group SNP-3-21386192-C (n = 279). (C) Accessions of the S allelic group SNP-3-21386192-T (n = 102). The different curves correspond to different analyses of GWA mapping. Dashed black line: expected; dashed red line: Wilcoxon; dashed blue line: EMMAX.
