# Supplementary material for: An Atypical Kinase under Balancing Selection Confers Broad-Spectrum Disease Resistance in Arabidopsis
Source: PLoS Genet. 2013 Sep 12;9(9):e1003766. doi: 10.1371/journal.pgen.1003766 (PMC3772041; doi:10.1371/journal.pgen.1003766)
Supplement: Figure S10 — Zoom on the association peaks around RKS1. Moccasin colored area indicates the eight putative kinases (including RKS1) region. (PDF) [file pgen.1003766.s010.pdf]

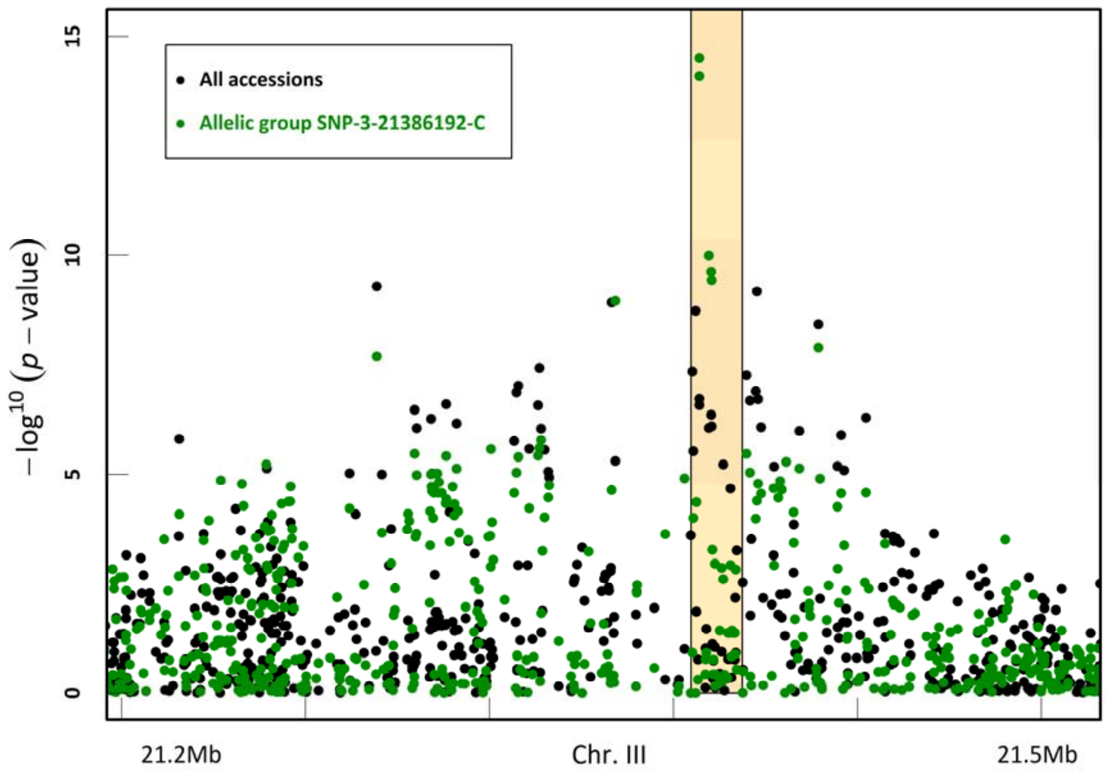

Figure S10. Zoom on the association peaks around *RKS1*. Moccasin colored area indicates the eight putative kinases (including *RKS1*) region.
