# Supplementary material for: An Atypical Kinase under Balancing Selection Confers Broad-Spectrum Disease Resistance in Arabidopsis
Source: PLoS Genet. 2013 Sep 12;9(9):e1003766. doi: 10.1371/journal.pgen.1003766 (PMC3772041; doi:10.1371/journal.pgen.1003766)
Supplement: Figure S11 — Whole-genome scan of 214,051 SNPs for association with disease index at 10 dpi across (A) the accessions of the S allelic group SNP-3-21386192-T (n = 102) and (B) the accessions of the R allelic group SNP-3-21386192-C (n = 279), using either the Wilcoxon model or the EMMAX method. The y-axis indicates the –log10 p-values using the Wilcoxon model or the EMMAX method. MARF = 0.05. (PDF) [file pgen.1003766.s011.pdf]

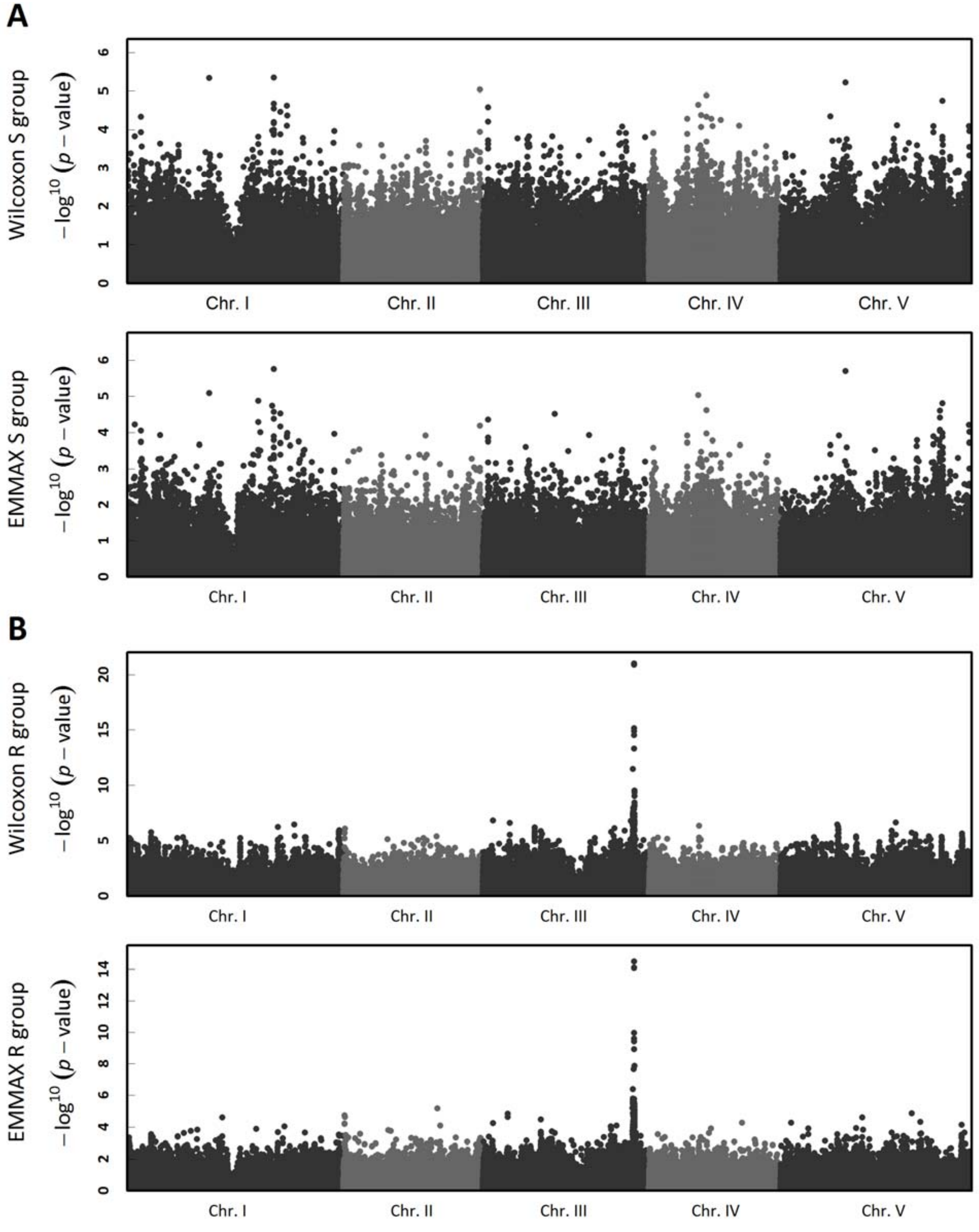

Figure S11. Whole-genome scan of 214,051 SNPs for association with disease index at 10 dpi across (A) the accessions of the S allelic group SNP-3-21386192-T (n = 102) and (B) the accessions of the R allelic group SNP-3-21386192-C (n = 279), using either the Wilcoxon model or the EMMAX method. The *y*-axis indicates the $-\log^{10}$ *p*-values using the Wilcoxon model or the EMMAX method. MARF = 0.05.
