# Supplementary material for: An Atypical Kinase under Balancing Selection Confers Broad-Spectrum Disease Resistance in Arabidopsis
Source: PLoS Genet. 2013 Sep 12;9(9):e1003766. doi: 10.1371/journal.pgen.1003766 (PMC3772041; doi:10.1371/journal.pgen.1003766)
Supplement: Figure S12 — Alignment of the 12 different RKS1 protein sequences from A. thaliana and putative RKS1 orthologous from Arabidopsis lyrata and Brassica rapa subsp. pekinensis. The major domains in the kinase catalytic core of kinase proteins described in Figure S6 are underlined in red. Numbering corresponds to the amino acid sequence. Every tenth residue in RKS1 is marked by *. A. lyrata NCBI Reference Sequence: XM_002878122.1. Brassica rapa subsp. pekinensis Sequence ID: gb|AC232509.1|. (PDF) [file pgen.1003766.s012.pdf]

```
                          *        20         *        40         *        60
Col-0    : MKKQYLKSGSGT--RKEKDKAKRWFLDNGSIFLRELVADCNGKSIPIRSFSPEQILKATN :  58
Bay-0    : MKK*YLKSGSGT--RKEKDKAKRWFLDNGSIFLRELVADCNGKSIPIRSFSPEQILKATN :  57
Bil-5    : MKKQYLKYGSGT--RKEKDKAKRWFLDNGNIFLSELVADCNGKSIPIRSFSPEQILKATN :  58
Br-0     : MKKQYLKSGSGT--RKEKDKAKRWFLDNGSIFLRELVADCNGKSIPIRSFSPEQILKATN :  58
Ct-1     : MKKQYLKSGSGT--RKEKDKAKRWFLDNGNIFLSELVADCNGKSIPIRSFSPEQILKATN :  58
Cvi-0    : MKKQYLKSGSGT--RKEKDKAKRWFLDNGSIFLRELVADCNGKSIPIRSFSPEQILKATN :  58
Ei-2     : MKKQYLKSGSGT--RKEKDKAKRWFLDNGNIFLSELVADCNGKSIPIRSFSPEQILKATN :  58
Est-1    : MKKQYLKSGSGT--RKEKDKAKRWFLDNGNIFLSELVADCNGKSIPIRSFSPEQILKATN :  58
Kas-1    : MKKQYLKSGSGT--RKEKDKAKRWFLDNGSIFLRELVADCNGKSIPIRSFSPEQILKATN :  58
Lp2-2    : MKKQYLKSGSGT--RKEKDKAKRWFLDNGSIFLRELVADCNGKSIPIRSFSPEQILKATN :  58
Nd-1     : MKKQYLKSGSGT--RKEKDKAKRWFLDNGNIFLSELVADCNGKSIPIRSFSPEQILKATN :  58
Zdr-1    : MKKQYLKSGSGT--RKEKDKAKRWFLDNGSIFLRELVADCNGKSIPIRSFSPEQILKATN :  58
Alyrata  : MKKQYLKSGSGSRTRKEKDKTKRWFLDNGSIFLTELVADCNGKSIPIRSFSPEQILKATN :  60
Brapa    : MKTQYLKSWSGR--KMEKERKKRWFLENGSLLLEELISDSNGKSIPIRSFPSDQILKATN :  58

                          *        80         *       100         *       120
Col-0    : NFDSSCFVSQDVYYKWYRGEIEDRSYMIKRFSEDEITGKRHRVKEVYNDIVLSARMSNHS : 118
Bay-0    : NFDSSCFVSQDVYYKWYRGEIEDRSYMIKRFSEDEITGKRHRVKEVYNDIVLSARMSNHS : 117
Bil-5    : NFDSSCFVSQDVYYKWYRGEIEDRSYMIKRFSEDEITGKRHRVKEVYNDIVLSARMSNHS : 118
Br-0     : NFDSSCFVSQDVYYKWYRGEIEDRSYMIKRFSEDEITGKRHRVKEVYNDIVLSARMSNHS : 118
Ct-1     : NFDSSCFVSQDVYYKWYRGEIEDRSYMIKRFSEDEITGKRHRVKEVYNDIVLSARMSNHS : 118
Cvi-0    : NFDSSCFVSQDVYYKWFRGEIEDRSYMIKRFSEDEITGKRHRVKEVYNDIVLSARMSNHS : 118
Ei-2     : NFDSSCFVSQDVYYKWYRGEIEDRSYMIKRFSEDEITGKRHRVKEVYNDIVLSARMSNHS : 118
Est-1    : NFDSSCFVSQDVYYKWYRGEIEDRSYMIKRFSEDEITGKRHRVKEVYNDIVLSARMSNHS : 118
Kas-1    : NFDSSCFVSQDVYYKWYRGEIEDRSYMIKRFSEDEITGKRHRVKEVYNDIVLSARMSNHS : 118
Lp2-2    : NFDSSCFVSQDVYYKWYRGEIEDRSYMIKRFSEDEITGKRHRVKEVYNDIVLSARMINHS : 118
Nd-1     : NFDSSCFVSQDVYYKWYRGEIEDRSYMIKRFSEDEITGKRHRVKEVYNDIVLSARMSNHS : 118
Zdr-1    : NFDSSCFVSQDVYYKWYRGEIEDRSYMIKRFSEDEITGKRHRVKEVYNDIVLSARMSNHS : 118
Alyrata  : NFDSSCFVSQDVYYKWYRGQIEDRSYMIKRFSEDEITGKRHRVKEVYNDIVLSARMSNHS : 120
Brapa    : NFDSSCFISEDAYYKWYRGNIEDRSYMIKKFSEDKVTG--HRVEEVYNDIVLSARMSNHN : 116
                   GxGxxG             VAVK

                          *       140         *       160         *       180
Col-0    : NFLQLLGCCLEFPFPVLVFEFAEHGAMNQRGGVIVNGEESLLPWSVRLKIGKEIANAVTY : 178
Bay-0    : NFLQLLGCCLEFPFPVLVFEFAEHGAMNQRGGVIVNGEESLLPWSVRLKIGKEIANAVTY : 177
Bil-5    : NFLQLLGCCLEFPFPVLVFEFAEHGAMNQRGGVIVNGEESLLPWSVRLKIGKEIANAVTY : 178
Br-0     : NFLQLLGCCLEFPFPVLVFEFAEHGAMNQRGGIIVNGEESLLPWSVRLKIGKEIANAVTY : 178
Ct-1     : NFLQLLGCCLEFPFPVLVFEFAEHGAMNQRGGVIVNGEESLLPWSVRLKIGKEIANAVTY : 178
Cvi-0    : NFLQLLGCCLEFPFPVLVFEFAEHGAMNQRGGVIVNGEESLLPWSVRLKIGKEIANAVTY : 178
Ei-2     : NFLQLLGCCLEFPFPVLVFEFAEHGAMNQRGGVIVNGEESLLPWSVRLKIGKEIANAVTY : 178
Est-1    : NFLQLLGCCLEFPFPVLVFEFAEHGAMNQRGGVIVNGEESLLPWSVRLKIGKEIANAVTY : 178
Kas-1    : NFLQLLGCCLEFPFPVLVFEFAEHGAMNQRGGVIVNGEESLLPWSVRLKIGKEIANAVTY : 178
Lp2-2    : NFLQLLGCCLEFPFPVLVFEFAEHGAMNQRGGVIVNGEESLLPWSVRLKIGKEIANAVTY : 178
Nd-1     : NFLQLLGCCLEFPFPVLVFEFAEHGAMNQRGGVIVNGEESLLPWSVRLKIGKEIANAVTY : 178
Zdr-1    : NFLQLLGCCLEFPFPVLVFEFAEHGAMNQRGGVIVNGEESLLPWSVRLKIGKEIANAVTY : 178
Alyrata  : NFLQLLGCCLEFPFPVLVFEFAEYGALNQRGGIMVNGEEYLLPWSVRLKIGKEIANAVSY : 180
Brapa    : NFLKLLGCSLEFPFPVLVFEFAGNGVLNERGGISVNGEDSLLPWGLRLKIGKEIANAVTY : 176

                          *       200         *       220         *       240
Col-0    : LHTAFPKIIIHRDVKPMHVFLDKNWTAKLSDLSFSISLPEGKSRIEAEWVLGTFGYIDPL : 238
Bay-0    : LHTAFPKIIIHRDVKPMHVFLDKNWTAKLSDLFFSISLPEGKSRIEAEWVLGTFGYIDPL : 237
Bil-5    : LHTAFPKIIIHRDVKPMHVFLDKNWTAKLSDLFFSISLPEGKSRIEAEWVLGTFGYIDPL : 238
Br-0     : LHTAFPKIIIHRDVKPMHVFLDKNWTAKLSDLSFSISLPEGKSRIEAEWVLGTFGYIDPL : 238
Ct-1     : LHTAFPKIIIHRDVKPMHVFLDKNWTAKLSDLSFSISLPEGKSRIEAEWVLGTFGYIDPL : 238
Cvi-0    : LHTAFPKIIIHRDVKPMHVFLDKNWTAKLSDLFFSISLPEGKSRIEAEWVLGTFGYIDPL : 238
Ei-2     : LHTAFPKIIIHRDVKPMHVFLDKNWTAKLSDLFFSISLPEGKSRIEAEWVLGTFGYIDPL : 238
Est-1    : LHTAFPKIIIHRDVKPMHVFLDKNWTAKLSDLSFSISLPEGKSRIEAEWVLGTFGYIDPL : 238
Kas-1    : LHTAFPKIIIHRDVKPMHVFLDKNWTAKLSDLFFSISLPEGKSRIEAEWVLGTFGYIDPL : 238
Lp2-2    : LHTAFPKIIIHRDVKPMHVFLDKNWTAKLSDLSFSISLPEGKSRIEAEWVLGTFGYIDPL : 238
Nd-1     : LHTAFPKIIIHKDVKPMHVFLDKNWTAKLSDLSFSISLPEGKSRIEAEWVLGTFGYIDPL : 238
Zdr-1    : LHTAFPKIIIHRDVKPMHVFLDKNWTAKLSDLSFSISLPEGKSRIEAEWVLGTFGYIDPL : 238
Alyrata  : LHMAFPKIIIHRDVKPMHVFLDKNWTAKLSDLSFSISLPEGKSRIEAEWVLGTFGYLDPL : 240
Brapa    : LHMAFPKIIIHRDVKPMHVFLDSNWTVKLSDLSFSISLPERKTRIEAEGIIGTFGYLDPL : 236
                     HRD                DFG                        APE
```

```
                       *        260         *        280         *        300
Col-0    : YHKTCFVTEYTDVYSFGICLLVIITGKPAIMTISDGDLQGILSLVRELCENGKLDEVIDP : 298
Bay-0    : YHKTCFVTEYTDVYSFGICLLVIITGKPAIMTISDGDLQGILSLVRELCENGKLDEVIDP : 297
Bil-5    : YHKTCFVTEYTDVYSFGICLLVIITGKPAIMTISDGDLQGILSLVRELCENGKLDEVIDP : 298
Br-0     : YHKTCFVTEYTDVYSFGICLLVIITGKPAIMTISDGDLQGILSLVRELCENGKLDEVIDP : 298
Ct-1     : YHKTCFVTEYTDVYSFGICLLVIITGKPAIMTISDGDLQGILSLVRELCENGKLDEVIDP : 298
Cvi-0    : YHKTCFVTEYTDVYSFGICLLVIITGKPAIMTISDGDLQGILSLVRELCENGKLDEVIDP : 298
Ei-2     : YHKTCFVTEYTDVYSFGICLLVIITGKPAIMTISDGDLQGILSLVRELCENGKLDEVIDP : 298
Est-1    : YHKTCFVTEYTDVYSFGICLLVIITGKPAIMTISDGDLQGILSLVRELCENGKLDEVIDP : 298
Kas-1    : YHKTCFVTEYTDVYSFGICLLVIITGKPAIMTISDGDLQGILSLVRELCENGKLDEVIDP : 298
Lp2-2    : YHKTCFVTEYTDVYSFGICLLVIITGKPAIMTISDGDLQGILSLVRELCENGKLDEVIDP : 298
Nd-1     : YHKTCFVTEYTDVYSFGICLLVIITGKPAIMTISDGDLQGILSLVRELCENGKLDEVIDP : 298
Zdr-1    : YHKTCFVTEYTDVYSFGICLLVIITGKPSIMTISDGDLQGILSLVRELCENGKLDEVIDP : 298
Alyrata  : YHKTSFVTEYTDVYSFGICFLVILTGKPAIITISDGDLQGILSYVRGLCENGKLDEVIDP : 300
Brapa    : YHATSFVTEYTDVYSFGICLLVLVTGKSVVITGSDGDPQGILSYVVGLWENGKVNEVIDP : 296
```

```
                       *        320         *        340         *
Col-0    : RLMKDITSGQRLQVEACVVLALRCCKERDEDRPKMIQVAKELKQIEASLKNSS : 351
Bay-0    : RLMKDITSGQRLQVEACVVLALRCCKERDEDRPKMIQVAKELKQIEASLKNSS : 350
Bil-5    : RLMKDITSGQRLQVEACVVLALRCCKERDEDRPKMIQVAKELKQIEASLKNSS : 351
Br-0     : RLMKDITSGQRLQVEACVVLALRCCKERDEDRPKMIQVAKELKQIEASLKNSS : 351
Ct-1     : RLMKDITSGQRLQVEACVVLALRCCKERDEDRPKMIQVAKELKQIEASLKNSS : 351
Cvi-0    : RLMKDITSGQRLQVEACVVLALRCCKERDEDRPKMIQVAKELKQIEASLKNSS : 351
Ei-2     : RLMKDITSGQRLQVEACVVLALRCCKERDEDRPKMIQVAKELKQIEASLKNSS : 351
Est-1    : RLMKDITSDQRLQVEACVVLALRCCKERDEDRPKMIQVAKELKQIEASLKNSS : 351
Kas-1    : RLMKDITSGQRLQVEACVVLALRCCKERDEDRPKMIQVAKELKQIEASLKNSS : 351
Lp2-2    : RLMKDITSGQRLQVEACVVLALRCCKERDEDRPKMIQVAKELKQIEASLKNSS : 351
Nd-1     : RLMKDITSGQRLQVEACVVLALRCCKERDEDRPKMIQVAKELKQIEASLKNSS : 351
Zdr-1    : RLMKDITSGQRLQVEACVVLALRCCKERDEDRPKMIQVAKELKQIEASLKNSS : 351
Alyrata  : MLMKDITSGQRLQVEACVVLALRCCEERDEYRPKVIQVAKELKRIEASLRNSS : 353
Brapa    : MIAKDITSGQKSQVEMCVGLALRCCEARDEYRPKMIQVAKELKLIEALLRDTS : 349
```

Figure S12. Alignment of the 12 different *RKS1* protein sequences from *A. thaliana* and putative *RKS1* orthologous from *Arabidopsis lyrata* and *Brassica rapa* subsp. *pekinensis*. The major domains in the kinase catalytic core of kinase proteins described in Figure S6 are underlined in red. Numbering corresponds to the amino acid sequence. Every tenth residue in RKS1 is marked by *. *A. lyrata* NCBI Reference Sequence: XM_002878122.1. *Brassica rapa* subsp. *pekinensis* Sequence ID: gb|AC232509.1|
