# Supplementary material for: An Atypical Kinase under Balancing Selection Confers Broad-Spectrum Disease Resistance in Arabidopsis
Source: PLoS Genet. 2013 Sep 12;9(9):e1003766. doi: 10.1371/journal.pgen.1003766 (PMC3772041; doi:10.1371/journal.pgen.1003766)
Supplement: Figure S13 — Relationship between disease index and expression levels of RKS1. (A) RKS1 total mRNA expression (RKS1-L+RKS1-S transcripts) considering all the accessions. (B) RKS1-L expression considering all the accessions. (C) RKS1-L expression considering all the accessions minus the accessions with a stop codon. (D) RKS1-L expression considering all the accessions minus the accessions with one of the two independent susceptible alleles within the R intergenic haplotype. Solid, dashed and dotted lines indicate negatively linear, exponentially decreasing and logarithmically decreasing functions, respectively. Parameters for each linear and non-linear model are given in Table S9. (PDF) [file pgen.1003766.s013.pdf]

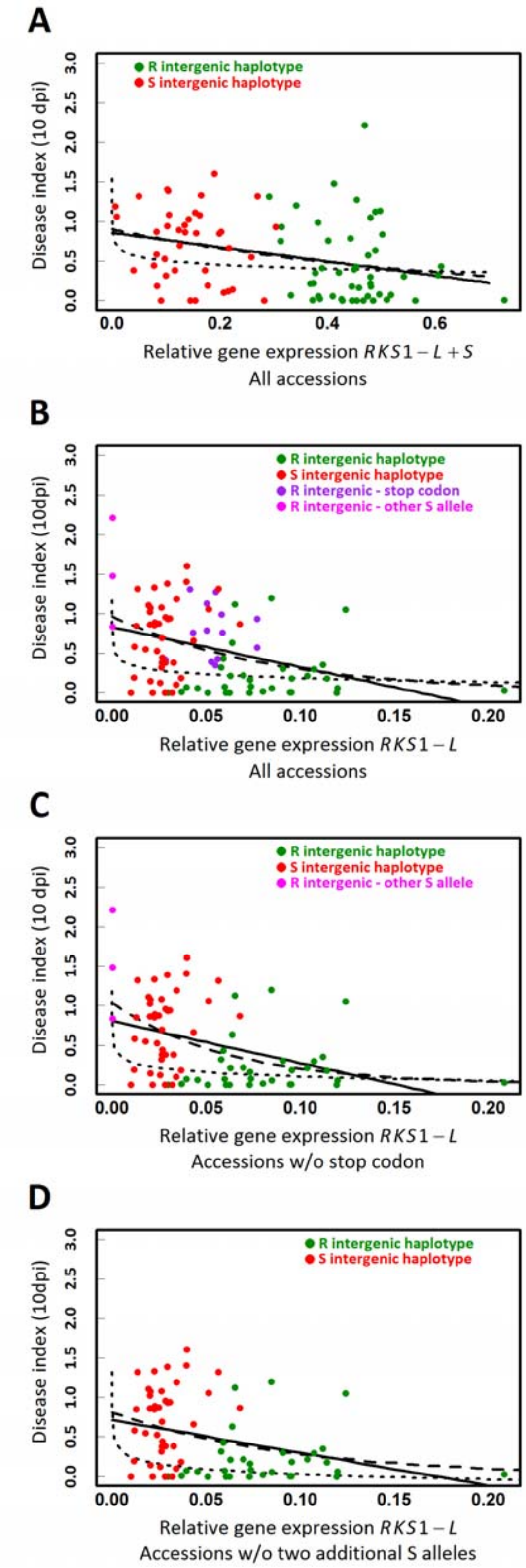

Figure S13. Relationship between disease index and expression levels of *RKS1*. (A) *RKS1* total mRNA expression (*RKS1-L*+ *RKS1-S* transcripts) considering all the accessions. (B) *RKS1-L* expression considering all the accessions. (C) *RKS1-L* expression considering all the accessions minus the accessions with a stop codon. (D) *RKS1-L* expression considering all the accessions minus the accessions with one of the two independent susceptible alleles within the R intergenic haplotype. Solid, dashed and dotted lines indicate

negatively linear, exponentially decreasing and logarithmically decreasing functions, respectively. Parameters for each linear and non-linear model are given in Table S9.
