# Supplementary material for: An Atypical Kinase under Balancing Selection Confers Broad-Spectrum Disease Resistance in Arabidopsis
Source: PLoS Genet. 2013 Sep 12;9(9):e1003766. doi: 10.1371/journal.pgen.1003766 (PMC3772041; doi:10.1371/journal.pgen.1003766)
Supplement: Figure S14 — Relationship between disease index and expression levels of At3g57720. No linear or non-linear relationship between disease index and relative gene expression of At3g57720 was detected. 1) linear model (disease∼intercept+a*expression): intercept = 0.336 (P = 0.0525), a = 0.661 (P = 0.1398). 2) exponential function (disease∼Ae-k*expression): A = 0.388 (P = 0.0017), k = −1.069 (P = 0.1529). 3) logarithmic function (disease∼b*log(expression)+c): b = −0.242 (P = 0.0990), c = −0.836 (P = 1.95×10^−6^). (PDF) [file pgen.1003766.s014.pdf]

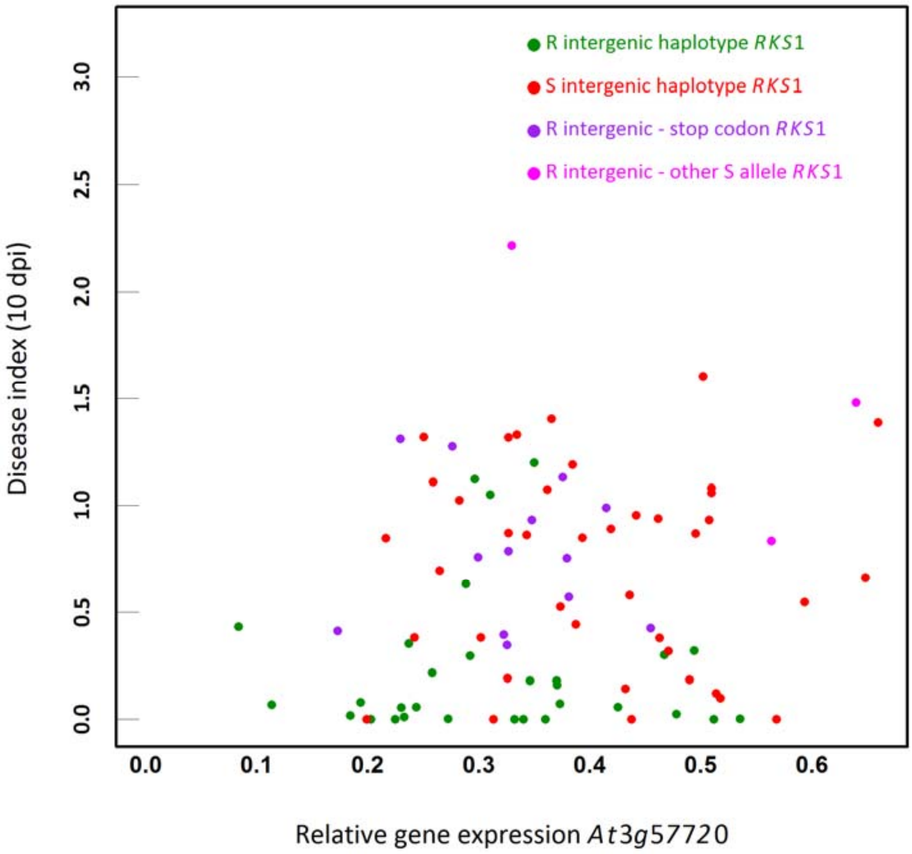

Figure S14. Relationship between disease index and expression levels of *At3g57720*. No linear or non-linear relationship between disease index and relative gene expression of *At3g57720* was detected. 1) linear model (disease ~ intercept + a*expression): intercept = 0.336 (P = 0.0525), a = 0.661 (P = 0.1398). 2) exponential function (disease ~ $Ae^{-k*expression}$): A = 0.388 (P = 0.0017), k = -1.069 (P = 0.1529). 3) logarithmic function (disease ~ b*log(expression) + c): b = -0.242 (P = 0.0990), c = -0.836 (P = 1.95 x 10^{-6}).
