# Supplementary material for: An Atypical Kinase under Balancing Selection Confers Broad-Spectrum Disease Resistance in Arabidopsis
Source: PLoS Genet. 2013 Sep 12;9(9):e1003766. doi: 10.1371/journal.pgen.1003766 (PMC3772041; doi:10.1371/journal.pgen.1003766)
Supplement: Figure S15 — Whole-genome scan of 214,051 SNPs for association with (A) the relative gene expression of RKS1-L+S (i.e. total mRNA) or (B) the relative gene expression of RKS1-L (n = 88). The y-axis indicates the –log10 p-values using the Wilcoxon model or the EMMAX method. MARF = 0.05. (PDF) [file pgen.1003766.s015.pdf]

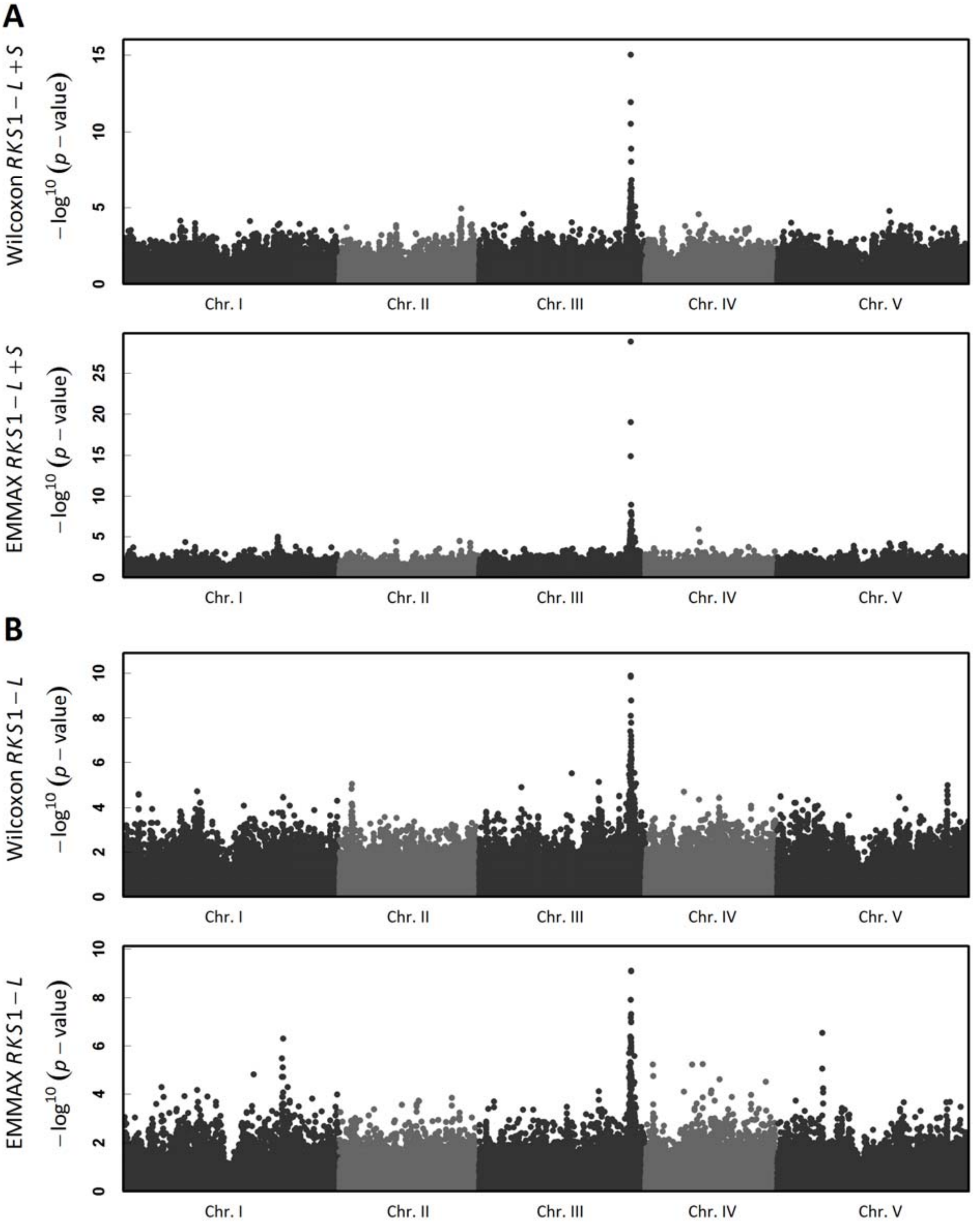

Figure S15. Whole-genome scan of 214,051 SNPs for association with (A) the relative gene expression of *RKS1-L+S* (i.e. total mRNA) or (B) the relative gene expression of *RKS1-L* (n = 88). The *y*-axis indicates the $-\log^{10}$ *p*-values using the Wilcoxon model or the EMMAX method. MARF = 0.05.
