# Supplementary material for: An Atypical Kinase under Balancing Selection Confers Broad-Spectrum Disease Resistance in Arabidopsis
Source: PLoS Genet. 2013 Sep 12;9(9):e1003766. doi: 10.1371/journal.pgen.1003766 (PMC3772041; doi:10.1371/journal.pgen.1003766)
Supplement: Figure S16 — Geographic distribution of RKS1 polymorphisms. (A) Geographic distribution of the two highly divergent haplotypes located in the intergenic region between RKS1 and At3g57720 and at the beginning of RKS1, using one of the 35 SNPs in complete LD, i.e. a SNP at position 21,387,232 on chromosome 3. (B) Geographic distribution of two S susceptible alleles embedded in the R intergenic haplogroup (n = 476). The geographic distribution of the accessions with a stop codon at the beginning of RKS1 was mapped using one of the 214,051 SNPs that is in complete LD with the stop codon; i.e. a SNP at position 21,388,948 on chromosome 3 (position 4129 in Table S8). The geographic distribution of the accessions with the additional S susceptible allele was mapped using accessions with both a ‘G’ base at position 21,388,849 on chromosome 3 (i.e. position 4030 in Table S8) and a ‘T’ base at position 21,389,085 on chromosome 3 (i.e. position 4266 in Table S8). All maps have been based on 948 natural accessions with accurate GPS coordinates and genotyped for 214,051 SNPs [65] and generated with the R packages ‘maptools’ and ‘plotrix’. The size of the pies depends of the number of accessions genotyped for 214,051 SNPs in the sites of collection. (PDF) [file pgen.1003766.s016.pdf]

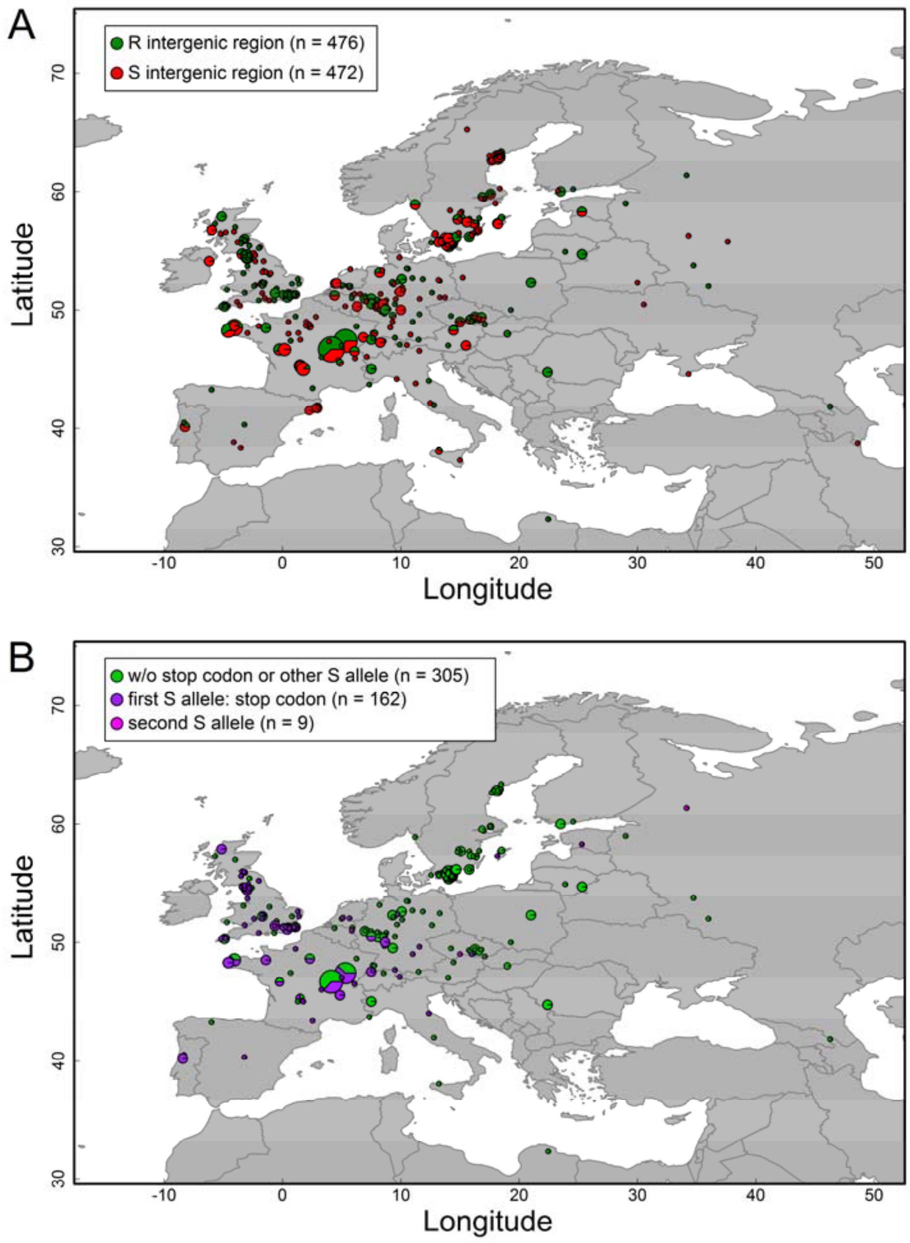

Figure S16. Geographic distribution of *RKS1* polymorphisms. (A) Geographic distribution of the two highly divergent haplotypes located in the intergenic region between *RKS1* and *At3g57720* and at the beginning of *RKS1*, using one of the 35 SNPs in complete LD, i.e. a SNP at position 21,387,232 on chromosome 3. (B) Geographic distribution of two S susceptible alleles embedded in the R intergenic haplogroup (n = 476). The geographic distribution of the accessions with a stop codon at the beginning of *RKS1* was mapped using one of the 214,051 SNPs that is in complete LD with the stop codon; i.e. a SNP at position 21,388,948 on chromosome 3 (position 4129 in Table S8). The geographic distribution of the accessions with the additional S susceptible allele was mapped using accessions with both a 'G' base at position 21,388,849 on chromosome 3 (i.e. position 4030 in Table S8) and a 'T' base at position 21,389,085 on chromosome 3 (i.e. position 4266 in Table S8). All maps have been based on 948 natural accessions with accurate GPS coordinates and genotyped for 214,051 SNPs [64] and generated with the *R* packages 'maptools' and 'plotrix'. The size of the pies depends of the number of accessions genotyped for 214,051 SNPs in the sites of collection.
