# Supplementary material for: An Atypical Kinase under Balancing Selection Confers Broad-Spectrum Disease Resistance in Arabidopsis
Source: PLoS Genet. 2013 Sep 12;9(9):e1003766. doi: 10.1371/journal.pgen.1003766 (PMC3772041; doi:10.1371/journal.pgen.1003766)
Supplement: Table S1 — Trait heritability and QTL detection. (PDF) [file pgen.1003766.s017.pdf]

Table S1. Trait heritability and QTL detection

Trait			QTL Analysis											
**Experiment[a]**	**RIL effect[b]**	**H^{2}[c]**	**Locus**	**DPI[d] 3***	**5**	**7**	**10**	**Chr**	**Position (cM)**	**max LOD[e]**	**Interval (map position)[f]**	**Var[g]**	**Effect[h]**	**Epistasis[i]**
Growth Chamber	$P < 0.0001$<br>$df = 112$<br>$F = 2.19$	0.75	*QRX1.1*	*ns*<br>*epi*<br>*epi*	*ns*	*ns*	***	I	31.1	2.3	[7 - 47]<br>1243559 - 13395602	11.1	-0.35	47(1):32(3)<br>2.8<br>24(1):21(4)<br>2
			QRX2	***	*ns*	*ns*	*ns*	II	69.1	2.40	[50 - 77]<br>9813845 - 18603183	12	-0.08	-
			QRX3	*ns*	***<br>*epi*	***<br><br>*epi*	***<br><br>*epi*<br>*epi*	III	104	17.8	[100.6 - 105.6]<br>18948842 - 22787634	53.7	-0.62	105(3):13(4)<br>2.8<br>104(3):27(1)<br>2.1 & 2.5<br>104(3):12(4)<br>3
			QRX5.1	*ns*	*ns*	***	***	IV	24	3.4	[14.29 - 37]<br>1586683 - 9448354	15.9	-0.41	-
			QRX5.2	***	***	*ns*	*ns*	IV	34.4	3.77	[17 - 41]<br>1586683 - 10551891	17.2	-0.22	-
Greenhouse	$P < 0.0001$<br>$df = 114$<br>$F = 8.16$	0.50	*QRX1.2*	***	***	***	*ns*	I	106	3.37	[94 - 114.1]<br>22388396 - 28841663	15.2	-0.25	-
			QRX3	*ns*	*ns*	***<br>*epi*	***<br><br>*epi*	III	104	5.22	[99 - 105.6]<br>18780383 - 23042168	22.1	-0.47	104(3):47(3)<br>2.3<br>*86(1):29(5)*<br>*2.4*
Bacterial Growth	$P = 0.0002$<br>$df = 94$<br>$F = 1.84$	0.17	*QRX3*		***<br>*epi*			III	102	2.63	[69 - 105]<br>13596040 - 23042168	15.9	-0.58	34(1):47(1)<br>2.1

[a] Three different experiments were conducted: the disease index was measured for both growth chamber and greenhouse grown 28 day-old plants and an IGC (Internal Growth Curve) experiment was conducted using growth chamber grown plants (see materials and methods).
[b] The RIL effect at 10 dpi was estimated using GLM procedure (Type III SS) with SAS software version 9.1 (SAS Institute, Cary, NC, USA).
[c] H^2: broad-sense heritability at 10 dpi
[d] QTL detection at different days post-inoculation (DPI) using the *scanone* function in Rqtl (www.r-project.org). * The disease index scoring was performed at 3, 5, 7, 10 and 4, 5, 7, 10 days post-inoculation for the growth chamber and greenhouse experiments, respectively. *ns*: no QTL was detected; *** QTL with Log of odds ratio ≥ 2.5 underscored: maximum LOD score; *epi*: significant digenic epistatic interaction detected (see Epistasis[i]).
[e] Maximum Log of odd ratio observed.
[f] Support intervals are given in Centimorgans and TAIR 9 coordinates were estimated by dropping 1.5 LOD units.
[g h] The proportion of phenotypic variance explained by each QTL and the estimated phenotypic effects corresponding to the allelic substitution from Kas-1 allele to Col-5 allele, were calculated in the context of multiple-QTL models using the function *fitqtl* of Rqtl. Values correspond to the results when the LOD is maximum. When a single QTL was considered, the heritability due to the QTL was estimated by $(1 - 10 ^ (-2 LOD / n))$, where *n* is the sample size.
[i] Significant pair-wise epistatic interactions were determined using interaction LOD scores from *scantwo* function in Rqtl: marker position in cM with chromosome in brackets for both markers followed by the interaction LOD score; in italic: significant interaction between markers outside of QTLs. The time at which the epistatic interaction occurred was indicated in the DPI[d] column (*epi*).
