# Supplementary material for: An Atypical Kinase under Balancing Selection Confers Broad-Spectrum Disease Resistance in Arabidopsis
Source: PLoS Genet. 2013 Sep 12;9(9):e1003766. doi: 10.1371/journal.pgen.1003766 (PMC3772041; doi:10.1371/journal.pgen.1003766)
Supplement: Table S2 — List of PCR-based marker and oligonucleotide sequences. (PDF) [file pgen.1003766.s018.pdf]

Table S2. List of PCR-based marker and oligonucleotide sequences.

Fine mapping

Marker	SNP(s) position on chr. 3 (bp)	Forward primer	Reverse primer
CAPS1	15965927-15966730	CGCTATTATGCCAAGTAAAGTGA	CCCATTTGACGAAGTCCGTTGA
F1P2TGF	17541237-17541435	TTTGTCTGAAGATGTGGAGAGAGAG	CAAAACCCCACTCTTCATTATTGTT
MS004	18432036-18432238	GCTTATGGCAAACAGAACTGA	CTCATACAGTCGTATGCCAT
R30025	18938781-18937861	GGTCAAAGCAACATTACATTGTA	TTGGATGAGATTGCAAGCCTTCA
MS007	19714028-19714287	CCACTCACGAACACTCCTCA	TTCCGGGTCAGGATGTGGTA
MSAT3.28	20456770-20456979	TACAAGTCATAATAGAGGC	GGGTTTAGCATTTAGC
MSAT002	20619908-20620142	GGAGCAGGATTTTCCACTGA	CCTGACAGTCTGGATGATGT
MSAT005	20762734-20763171	GGAGGCTTTAGCAGAAGAAGA	CCATCGCCACCATAGCTTCT
P10bisFterR	21323125- 21324699	GGTGAGGTTGTCTCAGTT	CATCCAGCTCTTTCCTCAGA
CAPS5	21621166-21621946	GGGAGAGTAGCACTGATCTT	CCATACGTTACGTAATGCTA
MSAT015	21718159-21718629	CTTCCCTTCTCATTCTGCT	CAGGCAGCTAATGCTAAGGAGTT
CAPS8	21916695-21917086	GGACTAAGTTCCTTGGAGACA	CGACCTAAGCCAGTACCAGAT
nga6	23031050- 23031192	ATGGAGAAGCTTACACTGATC	TGGATTTCTTCCTCTCTTCAC
nga112	23168570-23168372	CTCTCCACCTCCTCCAGTACC	TAATCACGTGTATGCAGCTGC
MS005	23377782-23378078	GCTCTACGTTACTTAAGCGA	CCAGACGATTCGTCCGAGA
3-57670	21371957-21372687	CTGTGAAGTCTGAGACTGA	CATGTCCCCACATATGCA
TK280-281	21375355-21376613	CATACAGTAATTGGAGGGAT	CTCCTTCAGATCAGCCTTGGA
1_57700	21384814-21386191	GTGACCTCGTTGACTTGA	GAGTAGGCATCACAAGA
c13-c14	21386759-21388774	GTCACCGCATTAGCAATCTCT	CATCTCTCAGCTCGCAGCA
QF-1R57730	21390902-21391774	TGCCTTTGAGTTTGAGGTTGA	CTCTCCCACGGAAAGAAGGT
IK01-02	21393979-21395425	CAGTAGTTGACTATCACAAAT	CTATCTATTTGGCTTATCCAC
c9-c10	21396684-21398587	GGTCATTGATCCCGACTACA	CCTGATCTGAATCCAACACA
c19-c20	21408735-21410203	CATTGGGTGTGTGTGTGTGT	GTGTGCCAAAGTAGCCAGT
3-57810	21416364-21416859	CATGTTCTAGGAATGCTATCAG	GGACACTTACCAATGATAGAGT
Indel1bis	21417631-21418039	GGATGCCAATCACAGTGT	CCTGATATCACACTGACCA
c21-c22	21428169-21430091	GTGTCATCACTTGTAGACGA	GTGGTCGTTTAGCTGAAGAGA
c25-c26	21440178-21442273	GTCATGGAATCTCTGTGCA	CTGCGCTCATGGATATCTCT
c1-c2	21447091-21449665	GGAGGGAAGAGCAATATCCGA	CCTGGTCACGAGACTTGTCA
c29-c30	21460205-21461470	CACAGTCACACACAAGACA	GTCTACTGTCTACAGACTCT
c5-c6	21477423-21478533	GCAGAGGTCCCAGAAACCGAA	CCAAGCATGTCACCGTTAGA
MSAT012bis	21509784-21510124	GACACGTGTTGGTGAAGA	GTCCGAGGCTTCGAAGCA
QF-QR_710	21387173-21387109	ACGGTAAATCCATTCCTATTCG	GAAATTGTTGGTGGCTTTCAA
QF-QR_720	21388622-21388696	AACATATTGCTAGCTACGTGAAAGG	CCATCACCTTAGGATCTATTACTCCA

Sequencing primers Col-0 and Kas-1

Marker	Forward primer	Reverse primer
RKS1-1	CATAAGTTGTTGACTTAGAC	TCAAGCAGATTGAAGCATCA
RKS1-2	CTAATGCACTTGAAACAGG	GAGGTGGTTCTTGGATAACGGA
RKS1-3	ACCTCTTTAACTCTATGTCG	ATACATAAAACCCCTCTGCGG
RKS1-4	CTGAGATCTGGATCGTTAGA	CCAAGTTCTGCATCTTGAAACAC
RKS1-5	ATCCACTAGCTCTGATGGGG	CACATCTACTTCCAGCTGAGT

Gene expression, constructions and transformant selection

	Gene	Name	Primer (5' - 3')
**Gene expression by Q-RT-PCR**	At3g57710	QF5_710	GCCCAAAAGAAGAGGTATCAG
		QR5_710	CGACTTGAAACAGGTCTATCTA
		QR_710	GAAATTGTTGGTGGCTTTCAA
		QRbis_710	TCCGTTATCCAAGAACCACCTC
	At3g57720	QF3_720	CGTTGTACTTGCGTTGAG
		QR3_720	AGCTATATGTACAAATCTGAGTTC
	At2g28390	TK322	AACTCTATGCAGCATTTGATCCACT
		TK323	TGATTGCATATCTTTATCGCCATC
**RKS1 constructions**	At3g57700	QFbis_57700	GAGCGAGGTTTGTTGACTGA
		attB1-QFbis_57700	CCTTCAGTCGACCCCGGGGAGCGAGGTTTGTTGACTGA
		1R_at3g57700	GAGTAGGCATCACAAGA
	At3g57710	attB1F-710	GGGGACAAGTTTGTACAAAAAAGCAGGCTTAATGAAGAAGCAG TATCTGAA
		attB1F-710	GGGGACCACTTTGTACAAGAAAGCTGGGTCGCTAGAATTTTTC AATGATGC
		C15	GTCCCAGATCCAGATTTCAGA
		Sqce_57710F	CTGCTTGAGTTCTTTAGCTACT
		Sqce_57710R	GTGATAGATCCGAGGTTGATG
	At3g57720	1R-at3g57720	CACATAGCCAGTAATACCTCT
		QRter_57720	CCTCTGCTACTCTGTATTGAG
		attB2-QRter_57720	CCGAGTGCGGCCGCCAGCAGCCTCTGCTACTCTGTATTGAG
**5' RACE**	At3g57710	710RACE-298R	TATGTCGTTTTCCTGTAATCTCATCC
		710RACE-356R	TTACTGTGATTACTCATCCGAGCAG
		710RACE-370R	AAAGTTGAAGAAAGTTACTGTGATTACT
		710RACE-686R	AGAACCCATTCAGCTTCTATTCTCG
		710RACE-908R	TCTTTCATCAACCTCGGATCTATCAC
		710RACE-959R	AAAGCCAGCACCACACATGCTTC
		710RACE-1026R	CTGCTTGAGTTCTTTAGCTACTTG
**3'RACE**	At3g57710	710race-1F	ATGAAGAAGCAGTATCTGAAATCTGG
		710race-47F	AGGACAAGGCGAAGAGGTGGTTCTT
		710race-213F	CTATAAATGGTATAGAGGTGAAATTGAA
		710race-273F	GGATGAGATTACAGGAAAACGACATAG
**amiRNA construction**	*RKS1* amiRNA	57710-I-miR01-s	GATTATAGTATACATCTTGCGGGTCTCTCTTTTGTATTCC
		57710-II-miR01-a	GACCCGCAAGATGTATACTATAATCAAAGAGAATCAATGA
		57710-III-miR01*s	GACCAGCAAGATGTAAACTATATTCACAGGTCGTGATATG
		57710-IV-miR01*a	GAATATAGTTTACATCTTGCTGGTCTACATATATATTCCT
	At3g57720 amiRNA	57720-I-miR01-s	GATAATTGGTCAGCATTGCGCTCTCTCTCTTTTGTATTCC
		57720-II-miR01-a	GAGAGCGCAATGCTGACCAATTATCAAAGAGAATCAATGA
		57720-III-miR01*s	GAGAACGCAATGCTGTCCAATTTTCACAGGTCGTGATATG
		57720-IV-miR01*a	GAAAATTGGACAGCATTGCGTTCTCTACATATATATTCCT
	RKS1+At3g57720 amiRNA	710/720-I-miR01-s	GATTTACCGTTACAATCAGGTACTCTCTCTTTTGTATTCC
		710/720-II-miR01-a	GAGTACCTGATTGTAACGGTAAATCAAAGAGAATCAATGA
		710/720-III-miR01*s	GAGTCCCTGATTGTATCGGTAATTCACAGGTCGTGATATG
		710/720-IV-miR01*a	GAATTACCGATACAATCAGGGACTCTACATATATATTCCT
	common	miR-A	CTGCAAGGCGATTAAGTTGGGTAAC
		miR-B	GCGGATAACAATTTCACACAGGAAACAG
		attB1-miR	GGGGACAAGTTTGTACAAAAAAGCAGGCTCCCCTCGAGGTCG ACGGTAT
		attB2-miR	GGGGACCACTTTGTACAAGAAAGCTGGGTCGCCAAGCTCGGA ATTAACC
