# Supplementary material for: An Atypical Kinase under Balancing Selection Confers Broad-Spectrum Disease Resistance in Arabidopsis
Source: PLoS Genet. 2013 Sep 12;9(9):e1003766. doi: 10.1371/journal.pgen.1003766 (PMC3772041; doi:10.1371/journal.pgen.1003766)
Supplement: Table S3 — Genotypes and phenotypes of 10 lines with the closest recombination events flanking QRX3. Line phenotypes in response to inoculation with Xcc568 were evaluated 6 and 9 days post-inoculation (R = resistant; S = susceptible; I = intermediate phenotype). Line genotypes: B: alleles of the susceptible parent Kas-1; A: alleles of the resistant parent Col-5; H: heterozygous. Primers are listed in Table S2. (PDF) [file pgen.1003766.s019.pdf]

**Table S3.** Genotypes and phenotypes of 10 lines with the closest recombination events flanking *QRX3*. Line phenotypes in response to inoculation with *Xcc568* were evaluated 6 and 9 days post-inoculation (R = resistant; S = susceptible; I = intermediate phenotype). Line genotypes: B: alleles of the susceptible parent Kas-1; A: alleles of the resistant parent Col-5; H: heterozygous. Primers are listed in Table S2.

| **Line Genotypes** | | | | | | | | | | | | | | | **Line phenotypes in response to *Xcc*** | | |
|---|---|---|---|---|---|---|---|---|---|---|---|---|---|---|---|---|---|
| *QRX3* locus | | | | | | | | | | | | | | | Disease Index | | Phenotype |
| **Lines** | Genes | 57670 | | | 57700 | 57710 | 57720 | 57730 | 57750 | 57770 | 57800 | 57810 | | | 6 dpi | 9 dpi | |
| | Markers | P10bisFterR | 3F_3R | TK280_281 | 1F_1R | QF-QR 710 | QF-QR 720 | QF_1R | ik01_ik02 | c9_c10 | c19_c20 | 3F_3R | indel1bis | | | | |
| **122** | | B | B | B | B | B | B | B | B | B | B | B | B | | 0,25 | 1,00 | **R** |
| **909** | | B | B | B | B | B | B | B | B | B | B | B | B | | 0,00 | 0,00 | **R** |
| **1011** | | H | B | B | B | B | B | B | B | B | B | B | B | | 0,25 | 1,50 | **R** |
| **411** | | H | H | H | H | H | H | H | H | B | B | B | B | | 1,50 | 2,50 | **I** |
| **803** | | H | H | H | H | H | H | H | H | H | H | H | B | | 1,50 | 2,25 | **I** |
| **903** | | B | H | H | H | H | H | H | H | H | H | H | H | | 1,75 | 2,25 | **I** |
| **388** | | A | H | H | H | H | H | H | H | H | H | H | H | | 1,00 | 2,25 | **I** |
| **442** | | A | A | A | A | A | A | A | A | A | A | A | A | | 3,00 | 3,50 | **S** |
| **507** | | H | A | A | A | A | A | A | A | A | A | A | A | | 2,50 | 3,25 | **S** |
| **685** | | H | H | H | A | A | A | A | A | A | A | A | A | | 2,75 | 3,25 | **S** |
