# Supplementary material for: An Atypical Kinase under Balancing Selection Confers Broad-Spectrum Disease Resistance in Arabidopsis
Source: PLoS Genet. 2013 Sep 12;9(9):e1003766. doi: 10.1371/journal.pgen.1003766 (PMC3772041; doi:10.1371/journal.pgen.1003766)
Supplement: Table S4 — Mutants identified in the QRX3 locus and their phenotype in response to inoculation to Xcc568. (PDF) [file pgen.1003766.s020.pdf]

Table S4. Mutants identified in the *QRX3* locus and their phenotype in response to inoculation to *Xcc568*.

Mutant #	Gene Accession	Mutant library[1]	Mutant Accession No	Disease Index[2]	Phenotype[3]
**1**	At3g57670	SALK	N507406	1.12	R
**2**	At3g57670	SALK	N655509	0.53	R
**3**	At3g57670	SAIL	N874719	0.19	R
**4**	At3g57680	SALK	N501461	1.20	R
**5**	At3g57680	SALK	N633692	1.27	R
**6**	At3g57680	SAIL	N808146	0.55	R
**7**	At3g57680	SAIL	N876763	0.61	R
**8**	At3g57690	Wisc	N849054	0.50	R
**9**	At3g57690	Wisc	N856964	0.67	R
**10**	At3g57700	SALK	N525615	0.49	R
**11**	**At3g57710**	SALK	**N630926**	**3.24**	**S**
**12**	At3g57710	SALK	N519218	0.45	R
**13**	At3g57720	SAIL	N841306	0.93	R
**14**	At3g57720	Wisc	N856501	0.17	R
**15**	At3g57730	Wisc	N858031	0.51	R
**16**	At3g57730	SAIL	N806177	0.37	R
**17**	At3g57730	GABI	191E08	0.42	R
**18**	At3g57740	CSHL	GT8984	0.11	R
**19**	At3g57750	SALK	N518065	0.47	R
**20**	At3g57760	SAIL	N839785	0.43	R
**21**	At3g57760	GABI	685C10	0.97	R
**22**	At3g57765	SALK	N565826	0.92	R
**23**	At3g57770	GABI	452B04	1.00	R
**24**	At3g57770	SAIL	N832413	0.79	R
**25**	At3g57780	GABI	849G11	0.32	R
**26**	At3g57785	SALK	N523082	0.94	R
**27**	At3g57785	SALK	N594682	0.03	R
**28**	At3g57790	SALK	N544657	0.75	R
**29**	At3g57790	SAIL	N825485	0.50	R
**30**	At3g57800	SALK	N502003	0.22	R
**31**	At3g57800	SAIL	N831493	0.82	R
**32**	At3g57800	SALK	N511022	0.11	R
**33**	At3g57800	SALK	N511030	ND	R
**34**	At3g57800	SALK	N526889	0.00	R
**35**	At3g57800	SALK	N526890	0.25	R
**36**	At3g57810	SALK	N647876	0.00	R
**Col-0**				0.94	R
**Kas-1**				3.77	S

[1] Mutant libraries from which the tested mutants have been identified are: SALK (http://signal.salk.edu), SAIL (lines from Syngenta, http://www.arabidopsis.org/abrc/sail.jsp), GABI-kat (http://www.gabi-kat.de), Wisconsin Ds-Lox (http://www.hort.wisc.edu/krysan/Ds-Lox) and CSHL (Cold Spring Harbor Laboratory, http://genetrap.cshl.org).
[2] Numbers are average of inoculation scores of 3-4 plants, and 4 leaves per plant, in at least 2 independent experiments, 10 days post-inoculation.
[3] The phenotype R (resistant) or S (susceptible) has been deduced from the evaluated disease index as previously defined 0-1.5 (resistant) and 3-4 (susceptible)
