# Supplementary material for: An Atypical Kinase under Balancing Selection Confers Broad-Spectrum Disease Resistance in Arabidopsis
Source: PLoS Genet. 2013 Sep 12;9(9):e1003766. doi: 10.1371/journal.pgen.1003766 (PMC3772041; doi:10.1371/journal.pgen.1003766)
Supplement: Table S5 — Fitting of one linear model and two non-linear models on the relationship between disease index and expression levels of RKS1 using various transgenic lines. (PDF) [file pgen.1003766.s021.pdf]

Table S5. Fitting of one linear model and two non-linear models on the relationship between disease index and expression levels of *RKS1* in RIL parental lines, mutant line, HIFs, complemented or silenced lines (see legend of Figure 4D).

			lm		exp		log	
disease index	*RKS1* gene expression	leaf	intercept	a	A	k	b	c
7dpi	*RKS1-L*	healthy	1.715 ***	-0.983 **	2.016 ***	1.731 **	-0.284 ***	0.522 ***
		infected	1.799 ***	-0.706 ***	2.061 ***	1.000 ***	-0.253 ***	0.635 ***
	RKS1-L+S	healthy	1.241 *	-0.161 ns	1.298 *	0.190 ns	-0.230 ns	1.021 **
		infected	1.489 **	-0.352 ns	1.579 **	0.361 ns	-0.377 ns	1.024 ***
10dpi	*RKS1-L*	healthy	2.700 ***	-1.280 ***	2.907 ***	0.910 **	-0.337 ***	1.208 ***
		infected	2.841 ***	-0.950 ***	3.018 ***	0.607 ***	-0.298 ***	1.343 ***
	RKS1-L+S	healthy	2.250 ***	-0.349 ns	2.443 **	0.264 ns	-0.511 ns	1.771 ***
		infected	2.605 ***	-0.617 ns	2.831 ***	0.389 ns	-0.686 *	1.788 ***

lm: linear model (disease ~ intercept + a*expression); exp: exponential function (disease ~ $Ae^{-k*expression}$); log: logarithmic function (disease ~ b*log(expression) + c). *0.05 > *P* > 0.01, **0.01 > *P* > 0.001, ****P* < 0.001.

expression: '*RKS1-L*': Relative gene expression (A.U.) of the *RKS1* long transcript; '*RKS1-L+S*': Relative gene expression (A.U.) of *RKS1* total mRNA.
