# Supplementary material for: An Atypical Kinase under Balancing Selection Confers Broad-Spectrum Disease Resistance in Arabidopsis
Source: PLoS Genet. 2013 Sep 12;9(9):e1003766. doi: 10.1371/journal.pgen.1003766 (PMC3772041; doi:10.1371/journal.pgen.1003766)
Supplement: Table S6 — Model selection among one linear model and two non-linear models on the relationship between disease index and expression levels of RKS1 using various transgenic lines. (PDF) [file pgen.1003766.s022.pdf]

Table S6. Model selection among one linear model and two non-linear models on the relationship between disease index and expression levels of *RKS1* in RIL parental lines, mutant line, HIFs, complemented or silenced lines (see legend of Figure 4D). For each 'disease index * *RKS1* gene expression * leaf' combination, different letters indicate different fits among the three models, with the lowest values indicating a better model fit.

					lm			exp			log		
disease index	*RKS1* gene expression	leaf	n	k	AIC	BIC	AICc	AIC	BIC	AICc	AIC	BIC	AICc
7dpi	*RKS1-L*	healthy	13	2	24.54 [a]	26.24 [a]	25.74 [a]	18.09 [b]	19.78 [b]	19.29 [b]	13.22 [c]	14.91 [c]	14.42 [c]
		infected	13	2	19.45 [a]	21.15 [a]	20.65 [a]	11.49 [b]	13.18 [b]	12.69 [b]	8.27 [c]	9.97 [c]	9.47 [c]
	RKS1-L+S	healthy	13	2	36.33 [a]	38.03 [a]	37.53 [a]	36.26 [a]	37.96 [a]	37.46 [a]	35.93 [a]	37.63 [a]	37.13 [a]
		infected	13	2	34.84 [a]	36.53 [a]	36.04 [a]	34.80 [a]	36.50 [a]	36.00 [a]	34.48 [a]	36.18 [a]	35.68 [a]
10dpi	*RKS1-L*	healthy	13	2	29.23 [a]	30.92 [a]	30.43 [a]	26.16 [b]	27.85 [b]	27.36 [b]	25.29 [b]	26.99 [b]	26.49 [b]
		infected	13	2	19.39 [a]	21.09 [a]	20.59 [a]	17.13 [a]	18.82 [a]	18.33 [a]	23.57 [b]	25.26 [b]	24.77 [b]
	RKS1-L+S	healthy	13	2	41.60 [a]	43.29 [a]	42.80 [a]	41.25 [a]	42.95 [a]	42.45 [a]	40.20 [a]	41.89 [a]	41.40 [a]
		infected	13	2	38.86 [a]	40.56 [a]	40.06 [a]	38.47 [a]	40.16 [a]	39.67 [a]	37.62 [a]	39.31 [a]	38.82 [a]

lm: linear model (disease ~ intercept + a*expression); exp: exponential function (disease ~ $Ae^{-k*expression}$); log: logarithmic function (disease ~ b*log(expression) + c). 'n' and 'k' denote the sample size and the number of parameters, respectively. AIC: Akaike's information criterion; BIC Bayesian information criterion; AICc: corrected AICc.

expression: '*RKS1-L*': Relative gene expression (A.U.) of the *RKS1* long transcript; '*RKS1-L+S*': Relative gene expression (A.U.) of *RKS1* total mRNA.
