# Supplementary material for: An Atypical Kinase under Balancing Selection Confers Broad-Spectrum Disease Resistance in Arabidopsis
Source: PLoS Genet. 2013 Sep 12;9(9):e1003766. doi: 10.1371/journal.pgen.1003766 (PMC3772041; doi:10.1371/journal.pgen.1003766)
Supplement: Table S7 — Disease index for 384 accessions used in this study. (PDF) [file pgen.1003766.s023.pdf]

Table S7. Disease index for 384 accessions used in this study.

[a] Type: WA = worldwide accession, FA = French accession as part of the French RegMap (Horton *et al.* 2012).

[b] Ecotype ID retrieved from 1,307 natural accessions genotyped using a 250K SNP chip (Horton et al. 2012).

dpi: days pots-inoculation, na: not available.

			Disease index		
**Native name**	**Type[a]**	**Ecotype ID[b]**	**5dpi**	**7dpi**	**10dpi**
**Ag-0**	**WA + FA**	**6897**	0.000	0.000	0.000
**Alc-0**	**WA**	**6988**	0.017	0.056	0.095
**Algutsrum**	**WA**	**8230**	0.003	0.246	1.379
**ALL1-2**	**FA**	**1**	0.000	0.018	0.000
**ALL1-5**	**FA**	**4**	0.025	0.054	1.116
**ALL1-6**	**FA**	**5**	0.013	0.063	0.346
**ALL1-8**	**FA**	**7**	0.000	0.000	0.071
**ALL1-9**	**FA**	**8**	0.000	0.809	1.293
**ALL2-1**	**FA**	**9**	0.503	0.773	0.926
**ALL2-4**	**FA**	**12**	.	.	.
**An-1**	**WA**	**6898**	0.254	0.549	0.848
**Ang-0**	**WA**	**8254**	0.000	0.000	0.121
**Ann-1**	**WA + FA**	**6994**	0.000	0.035	0.308
**Ba1-2**	**WA**	**8256**	0.001	0.237	0.357
**Ba4-1**	**WA**	**8258**	0.163	0.651	1.314
**Ba5-1**	**WA**	**8259**	0.000	0.791	1.740
**Bay-0**	**WA**	**6899**	0.000	0.000	0.412
**Bg-2**	**WA**	**6709**	0.127	0.117	0.243
**Bil-5**	**WA**	**6900**	0.000	0.326	1.319
**Bil-7**	**WA**	**6901**	0.004	0.131	1.025
**Bla-1**	**WA**	**8264**	0.000	0.269	0.532
**Blh-1**	**WA**	**7034**	0.000	0.000	0.000
**Boo2-1**	**WA**	**8266**	0.003	0.001	0.140
**Bor-1**	**WA**	**5837**	0.000	0.114	0.217
**Bor-4**	**WA**	**6903**	0.006	0.021	0.058
**Br-0**	**WA**	**6904**	0.007	0.151	0.069
**Bro1-6**	**WA**	**8231**	0.250	0.248	0.746
**Bs-1**	**WA**	**8270**	0.000	0.000	0.364
**Bu-0**	**WA**	**8271**	0.000	0.000	0.000
**Buckhorn Pass**	**WA**	**7033**	0.002	0.000	0.243
**Bur-0**	**WA**	**6905**	0.001	0.003	0.382
**C24**	**WA**	**6906**	0.153	0.163	0.632
**CAM-16**	**FA**	**23**	0.004	0.006	0.275
**CAM-25**	**FA**	**32**	0.000	0.000	0.088
**CAM-42**	**FA**	**45**	0.003	0.398	0.926
**CAM-56**	**FA**	**60**	0.003	0.001	0.265
**CAM-58**	**FA**	**62**	0.000	0.459	0.897
**CAM-61**	**FA**	**66**	0.000	0.000	0.000
**CAM-64**	**FA**	**69**	0.128	0.393	1.040
**CAM-7**	**FA**	**74**	0.129	0.881	1.400
**Can-0**	**WA**	**8274**	0.250	0.873	1.371
**Cen-0**	**WA + FA**	**8275**	0.011	0.000	0.930
**Chat-1**	**WA + FA**	**7071**	0.001	0.000	0.107

**CIBC-17**	**WA**	**6907**	0.007	0.031	0.080
**CIBC-5**	**WA**	**6908**	0.124	0.113	0.850
**CLA-7**	**FA**	**77**	0.373	0.337	0.428
**Co**	**WA**	**7081**	0.000	0.218	0.681
**Col-0**	**WA**	**6909**	0.000	0.009	0.011
**Ct-1**	**WA**	**6910**	0.003	0.000	0.379
**CUR-10**	**FA**	**79**	0.001	0.003	0.132
**CUR-2**	**FA**	**80**	0.015	0.043	0.732
**CUR-3**	**FA**	**81**	0.252	0.513	0.904
**CUR-4**	**FA**	**82**	0.504	1.506	2.025
**CUR-5**	**FA**	**83**	0.002	0.000	0.243
**CUR-6**	**FA**	**84**	0.000	0.587	0.928
**CUR-7**	**FA**	**85**	0.000	0.223	0.317
**CUR-8**	**FA**	**86**	0.250	0.727	1.210
**CUR-9**	**FA**	**87**	0.000	0.000	0.830
**Cvi-0**	**WA**	**6911**	1.674	1.880	2.213
**CYR**	**FA**	**88**	0.005	0.016	0.047
**DAM1**	**FA**	**89**	0.000	0.249	0.989
**Dem-4**	**WA**	**8233**	0.000	0.001	0.107
**Di-1**	**WA + FA**	**7098**	0.324	0.809	1.283
**Dra-3-1**	**WA**	**8283**	0.003	0.001	0.140
**DraII-1**	**WA**	**8284**	0.001	0.000	0.000
**DraIII-1**	**WA**	**8285**	0.000	0.244	0.353
**Duk**	**WA**	**6008**	0.001	0.024	0.043
**Eden-1**	**WA**	**6009**	0.127	0.492	0.868
**Eden-2**	**WA**	**6913**	0.000	1.145	1.318
**Edi-0**	**WA**	**6914**	0.000	0.000	0.000
**Eds-1**	**WA**	**6016**	0.000	0.000	0.151
**Ei-2**	**WA**	**6915**	0.009	0.296	0.862
**En-1**	**WA**	**8290**	0.000	0.259	0.386
**Est-1**	**WA**	**6916**	0.018	0.375	1.075
**Fab-2**	**WA**	**6917**	0.124	0.467	0.939
**Fab-4**	**WA**	**6918**	0.000	0.000	0.317
**Fei-0**	**WA**	**8215**	0.004	0.777	1.311
**Fja1-1**	**WA**	**8422**	0.001	0.008	0.018
**Ga-0**	**WA**	**6919**	0.000	0.098	0.442
**Gd-1**	**WA**	**8296**	0.005	0.266	0.422
**Ge-0**	**WA**	**8297**	0.376	0.503	0.882
**Got-22**	**WA**	**6920**	0.000	0.100	0.185
**Got-7**	**WA**	**6921**	0.665	0.692	1.058
**Gr-1**	**WA**	**8300**	0.118	0.512	1.121
**Gu-0**	**WA**	**7149**	0.120	0.360	0.581
**Gy-0**	**WA + FA**	**8214**	0.023	0.581	1.404
**H55**	**WA**	**7461**	0.002	0.013	0.654
**Hi-0**	**WA**	**8304**	0.001	0.000	0.000
**Hod**	**WA**	**8235**	0.002	0.013	0.029
**Hov2-1**	**WA**	**8423**	0.000	0.011	0.128
**Hov4-1**	**WA**	**8306**	0.000	0.342	1.314
**Hovdala-2**	**WA**	**6039**	0.000	0.000	0.475
**HR-10**	**WA**	**6923**	0.128	0.121	0.754

**HR-5**	**WA**	**6924**	0.163	0.154	0.989
**Hs-0**	**WA**	**8310**	0.003	0.001	0.390
**HSm**	**WA**	**8236**	0.000	0.004	0.125
**In-0**	**WA**	**8311**	0.000	0.000	0.114
**Is-0**	**WA**	**8312**	0.000	0.000	0.074
**JEA**	**FA**	**91**	0.329	0.692	0.542
**Jm-0**	**WA**	**8313**	0.000	0.000	0.000
**Ka-0**	**WA**	**8314**	0.000	0.000	0.103
**Kelsterbach-4**	**WA**	**8420**	0.000	0.486	0.824
**Kent**	**WA**	**8238**	0.006	0.021	0.808
**Kin-0**	**WA**	**6926**	0.126	0.628	1.132
**Kni-1**	**WA**	**6040**	0.000	0.369	0.478
**Kno-10**	**WA**	**6927**	0.257	0.281	0.955
**Kno-18**	**WA**	**6928**	0.000	0.122	0.213
**Koln**	**WA**	**8239**	0.000	0.000	0.000
**Kondara**	**WA**	**6929**	0.255	0.537	0.833
**Kulturen-1**	**WA**	**8240**	0.000	0.014	0.022
**Kz-1**	**WA**	**6930**	0.120	0.860	1.331
**Kz-9**	**WA**	**6931**	0.001	0.253	0.382
**LAC-3**	**FA**	**94**	0.124	0.014	0.022
**LAC-5**	**FA**	**96**	0.000	0.165	0.319
**Lc-0**	**WA**	**8323**	0.000	0.000	0.000
**LDV-14**	**FA**	**104**	0.000	0.097	0.074
**LDV-16**	**FA**	**106**	0.001	0.008	0.143
**LDV-25**	**FA**	**116**	0.000	0.000	0.676
**LDV-3**	**FA**	**121**	0.251	0.758	1.643
**LDV-30**	**FA**	**122**	0.255	0.636	0.911
**LDV-31**	**FA**	**123**	0.000	0.000	0.110
**LDV-34**	**FA**	**126**	0.003	0.023	0.051
**LDV-44**	**FA**	**137**	0.004	0.006	0.275
**LDV-53**	**FA**	**146**	0.002	0.000	0.368
**LDV-57**	**FA**	**148**	.	.	.
**LDV-58**	**FA**	**149**	0.005	0.011	0.036
**LDV-6**	**FA**	**151**	0.123	0.275	0.536
**LDV-68**	**FA**	**153**	0.158	0.826	1.136
**LDV-70**	**FA**	**156**	0.003	0.007	0.975
**LDV-8**	**FA**	**157**	0.006	0.021	0.058
**Ler-1**	**WA**	**6932**	0.000	0.000	0.000
**Liarum**	**WA**	**8241**	0.000	0.000	0.228
**Lillo-1**	**WA**	**8242**	0.000	0.040	0.069
**Lip-0**	**WA**	**8325**	0.246	0.697	1.020
**Lis-1**	**WA**	**8326**	0.000	0.000	0.000
**Lis-2**	**WA**	**8222**	0.000	0.000	0.000
**Lisse**	**WA**	**8430**	0.000	0.116	0.085
**LL-0**	**WA**	**6933**	0.000	0.306	1.109
**Lm-2**	**WA + FA**	**8329**	0.120	0.360	0.456
**Lom1-1**	**WA**	**6042**	0.000	0.049	0.057
**Lov-1**	**WA**	**6043**	0.000	0.108	0.214
**Lov-5**	**WA**	**6046**	0.001	0.008	0.018
**Lp2-2**	**WA**	**7520**	0.000	0.479	1.123

**Lp2-6**	**WA**	**7521**	0.252	0.284	1.190
**Lu-1**	**WA**	**8334**	0.000	0.000	0.250
**Lund**	**WA**	**8335**	0.000	0.221	1.233
**Lz-0**	**WA + FA**	**6936**	0.491	1.139	1.275
**MAR2-3**	**FA**	**159**	0.000	0.118	0.735
**MIB-1**	**FA**	**160**	0.000	0.000	0.185
**MIB-100**	**FA**	**162**	0.001	0.000	0.000
**MIB-11**	**FA**	**163**	0.124	0.000	0.689
**MIB-14**	**FA**	**165**	0.003	0.621	1.379
**MIB-15**	**FA**	**166**	0.001	0.000	0.000
**MIB-16**	**FA**	**167**	0.005	0.011	0.161
**MIB-17**	**FA**	**168**	0.000	0.114	0.842
**MIB-2**	**FA**	**170**	0.001	0.000	0.000
**MIB-22**	**FA**	**173**	0.151	0.974	1.416
**MIB-23**	**FA**	**174**	0.000	0.123	0.621
**MIB-24**	**FA**	**175**	0.118	0.319	1.252
**MIB-28**	**FA**	**178**	0.182	1.022	2.032
**MIB-29**	**FA**	**179**	0.000	0.119	0.760
**MIB-32**	**FA**	**183**	0.000	0.000	0.114
**MIB-33**	**FA**	**184**	0.003	0.023	0.426
**MIB-34**	**FA**	**185**	0.000	0.000	0.453
**MIB-35**	**FA**	**186**	0.000	0.000	0.110
**MIB-36**	**FA**	**187**	0.002	0.034	0.190
**MIB-37**	**FA**	**188**	0.251	0.904	1.679
**MIB-39**	**FA**	**190**	0.001	0.029	0.054
**MIB-40**	**FA**	**191**	0.004	0.006	0.150
**MIB-43**	**FA**	**194**	0.001	0.029	0.054
**MIB-47**	**FA**	**196**	0.007	0.026	0.069
**MIB-50**	**FA**	**198**	0.000	0.105	0.446
**MIB-54**	**FA**	**200**	0.005	0.141	0.672
**MIB-55**	**FA**	**201**	0.249	0.863	0.975
**MIB-57**	**FA**	**202**	0.504	0.631	1.400
**MIB-58**	**FA**	**203**	0.000	0.134	0.386
**MIB-60**	**FA**	**204**	0.001	0.008	0.268
**MIB-61**	**FA**	**205**	0.002	0.013	0.154
**MIB-62**	**FA**	**206**	0.000	0.106	0.313
**MIB-63**	**FA**	**207**	0.000	0.000	0.000
**MIB-64**	**FA**	**208**	0.000	0.000	0.071
**MIB-67**	**FA**	**210**	0.344	0.539	1.541
**MIB-69**	**FA**	**212**	0.003	0.000	0.004
**MIB-70**	**FA**	**213**	0.000	0.000	0.000
**MIB-73**	**FA**	**214**	0.741	0.768	0.750
**MIB-75**	**FA**	**215**	0.000	0.000	0.000
**MIB-76**	**FA**	**216**	0.001	0.000	0.096
**MIB-77**	**FA**	**217**	0.129	0.256	0.525
**MIB-80**	**FA**	**219**	0.000	0.020	0.025
**MIB-83**	**FA**	**222**	0.251	0.149	0.418
**MIB-84**	**FA**	**223**	0.124	0.113	0.725
**MIB-86**	**FA**	**224**	0.002	0.000	0.118
**MIB-87**	**FA**	**225**	0.000	0.000	0.818

**MIB-89**	**FA**	**227**	0.003	0.023	0.301
**MIB-9**	**FA**	**228**	0.131	0.521	0.683
**MIB-90**	**FA**	**229**	0.000	0.000	0.000
**MIB-92**	**FA**	**230**	0.000	0.493	0.485
**MIB-93**	**FA**	**231**	0.750	1.248	1.496
**Mir-0**	**WA**	**8337**	0.000	0.000	0.333
**MOG-11**	**FA**	**236**	0.000	0.124	1.489
**MOG-12**	**FA**	**237**	0.001	0.000	0.232
**MOG-37**	**FA**	**242**	0.000	0.373	0.621
**MOG-40**	**FA**	**244**	0.180	0.342	0.836
**MOG-55**	**FA**	**252**	0.251	0.237	0.232
**Mr-0**	**WA**	**7522**	0.000	0.000	0.000
**Mrk-0**	**WA**	**6937**	0.000	0.000	0.000
**Ms-0**	**WA**	**6938**	0.000	0.000	0.000
**Mt-0**	**WA**	**6939**	0.000	0.000	0.000
**Mz-0**	**WA**	**6940**	0.016	0.022	1.199
**N13**	**WA**	**7438**	0.001	0.008	0.018
**Na-1**	**WA + FA**	**8343**	0.000	0.009	0.761
**Nc-1**	**WA + FA**	**7430**	0.000	0.019	0.157
**Nd-1**	**WA**	**6942**	0.005	0.266	0.547
**NFA-10**	**WA**	**6943**	0.000	0.000	0.431
**NFA-8**	**WA**	**6944**	0.017	0.035	0.394
**Nok-1**	**WA**	**7270**	0.001	0.503	0.757
**Nok-3**	**WA**	**6945**	0.000	0.000	0.192
**Nw-0**	**WA**	**7258**	0.000	0.000	0.031
**Nyl-2**	**WA**	**6064**	0.000	0.369	0.978
**Omo-2-1**	**WA**	**7518**	0.004	0.006	0.025
**Omo-2-3**	**WA**	**7519**	0.000	0.000	0.000
**Or-1**	**WA**	**6074**	0.251	0.378	0.882
**Ost-0**	**WA**	**8351**	0.000	0.342	0.564
**Oy-0**	**WA**	**6946**	0.000	0.000	0.121
**Pa-1**	**WA**	**8353**	0.496	0.809	1.460
**PAR-10**	**FA**	**257**	0.263	0.270	0.774
**PAR-3**	**FA**	**258**	0.000	0.000	0.000
**PAR-4**	**FA**	**259**	0.005	0.516	0.922
**PAR-6**	**FA**	**261**	0.000	0.000	0.121
**PAR-8**	**FA**	**262**	0.001	0.357	1.096
**PAR-9**	**FA**	**263**	0.004	0.006	0.025
**Per-1**	**WA**	**8354**	0.008	0.036	0.091
**Petergof**	**WA**	**7296**	0.000	0.000	0.000
**PHW-2**	**WA**	**8243**	0.001	0.107	0.471
**PHW-36**	**WA + FA**	**7507**	0.003	0.121	0.504
**PHW-37**	**WA + FA**	**7508**	0.013	0.030	0.037
**Pla-0**	**WA**	**7300**	0.124	0.384	0.886
**Pn-0**	**WA + FA**	**7307**	0.001	0.003	0.382
**Pna-10**	**WA**	**7526**	0.003	0.126	0.265
**Pna-17**	**WA**	**7523**	0.124	0.384	0.636
**PON**	**FA**	**264**	0.000	0.217	0.939
**Pro-0**	**WA**	**8213**	0.000	0.119	0.353
**Pu2-23**	**WA**	**6951**	0.000	0.000	0.000

**Pu2-7**	**WA**	**6956**	0.000	0.009	0.000
**Pu2-8**	**WA**	**6957**	0.000	0.000	0.000
**Ra-0**	**WA + FA**	**6958**	0.378	0.898	1.051
**Rak-2**	**WA**	**8365**	0.003	0.164	0.201
**RAN**	**FA**	**266**	0.016	0.069	0.106
**Rd-0 CS1482**	**WA**	**8366**	0.000	0.113	0.600
**Rd-0 CS1483**	**WA**	**8411**	0.002	0.263	0.529
**Ren-1**	**WA + FA**	**6959**	0.001	0.232	0.346
**Ren-11**	**WA + FA**	**6960**	0.014	0.065	0.933
**Rev-1**	**WA**	**8369**	.	.	.
**Ri-0**	**WA**	**7317**	0.001	0.000	0.000
**Rmx-A02**	**WA**	**7524**	0.253	0.398	0.426
**Rmx-A180**	**WA**	**7525**	0.376	0.628	0.757
**ROM-9**	**FA**	**269**	0.001	0.003	0.382
**Rou-0**	**WA + FA**	**7320**	0.008	0.036	0.716
**RRS-10**	**WA**	**7515**	0.129	0.277	0.436
**RRS-7**	**WA**	**7514**	0.130	0.386	0.786
**Rsch-4**	**WA**	**8374**	0.007	0.031	0.830
**Rubezhnoe-1**	**WA**	**7323**	0.255	0.766	1.422
**San-2**	**WA**	**8247**	0.379	0.527	1.686
**Sanna-2**	**WA**	**8376**	0.000	0.113	0.475
**Sap-0**	**WA**	**8378**	0.000	0.373	0.871
**Sav-0**	**WA**	**7340**	0.003	0.126	0.640
**Se-0**	**WA**	**6961**	0.249	0.759	1.386
**Seattle-0**	**WA**	**8245**	0.128	0.746	1.379
**Shahdara**	**WA**	**6962**	0.000	0.360	1.081
**Sorbo**	**WA**	**6963**	0.000	0.817	1.481
**Spr-1-2**	**WA**	**6964**	0.000	0.000	0.000
**Spr-1-6**	**WA**	**6965**	0.005	0.261	0.661
**Sq-1**	**WA**	**6966**	0.004	0.006	0.525
**Sq-8**	**WA**	**6967**	0.000	0.205	0.571
**Sr:5**	**WA**	**8386**	0.003	0.001	0.015
**St-0**	**WA**	**8387**	0.002	0.000	0.743
**Stw-0**	**WA**	**8388**	0.000	0.011	0.128
**Ta-0**	**WA**	**8389**	0.007	0.026	0.069
**Tamm-2**	**WA**	**6968**	0.000	0.000	0.056
**Tamm-27**	**WA**	**6969**	0.000	0.000	0.495
**Tottarp-2**	**WA**	**6243**	0.253	0.643	1.165
**TOU-A1-105**	**FA**	**273**	0.002	0.013	0.029
**TOU-A1-109**	**FA**	**275**	0.000	0.000	0.000
**TOU-A1-111**	**FA**	**277**	0.002	0.000	0.368
**TOU-A1-112**	**FA**	**278**	0.000	0.000	0.000
**TOU-A1-114**	**FA**	**280**	0.000	0.000	0.199
**TOU-A1-115**	**FA**	**281**	0.005	0.261	0.661
**TOU-A1-116**	**FA**	**282**	0.016	0.078	0.962
**TOU-A1-117**	**FA**	**283**	0.000	0.169	0.508
**TOU-A1-120**	**FA**	**287**	0.002	0.159	0.190
**TOU-A1-122**	**FA**	**288**	0.002	0.013	0.279
**TOU-A1-124**	**FA**	**290**	0.000	0.000	0.000
**TOU-A1-125**	**FA**	**291**	0.003	0.000	0.004

**TOU-A1-128**	**FA**	**292**	0.125	0.144	0.407
**TOU-A1-129**	**FA**	**293**	0.003	0.001	0.015
**TOU-A1-131**	**FA**	**295**	0.005	0.011	0.036
**TOU-A1-133**	**FA**	**296**	0.003	0.001	0.015
**TOU-A1-134**	**FA**	**297**	0.000	0.000	0.000
**TOU-A1-137**	**FA**	**298**	0.004	0.027	0.061
**TOU-A1-143**	**FA**	**302**	0.004	0.131	0.525
**TOU-A1-18**	**FA**	**306**	0.003	0.126	0.515
**TOU-A1-23**	**FA**	**309**	0.000	0.000	0.196
**TOU-A1-26**	**FA**	**310**	0.000	0.659	1.303
**TOU-A1-27**	**FA**	**311**	0.327	0.297	0.271
**TOU-A1-33**	**FA**	**314**	0.003	0.000	0.004
**TOU-A1-37**	**FA**	**316**	0.000	0.000	0.000
**TOU-A1-39**	**FA**	**318**	0.255	0.641	1.047
**TOU-A1-41**	**FA**	**320**	0.000	0.123	0.746
**TOU-A1-43**	**FA**	**321**	0.005	0.016	0.047
**TOU-A1-45**	**FA**	**322**	0.000	0.616	0.835
**TOU-A1-47**	**FA**	**323**	0.000	0.000	0.000
**TOU-A1-60**	**FA**	**326**	0.000	0.000	0.000
**TOU-A1-61**	**FA**	**327**	0.000	0.000	0.000
**TOU-A1-62**	**FA**	**328**	0.003	0.000	0.004
**TOU-A1-63**	**FA**	**329**	0.000	0.000	0.585
**TOU-A1-65**	**FA**	**331**	0.000	0.000	0.235
**TOU-A1-66**	**FA**	**332**	0.501	0.487	0.607
**TOU-A1-68**	**FA**	**334**	0.003	0.126	0.640
**TOU-A1-69**	**FA**	**335**	0.002	0.138	0.529
**TOU-A1-70**	**FA**	**337**	0.251	0.503	0.507
**TOU-A1-73**	**FA**	**338**	0.004	0.006	0.025
**TOU-A1-74**	**FA**	**339**	0.000	0.016	0.639
**TOU-A1-75**	**FA**	**340**	0.001	0.000	0.107
**TOU-A1-77**	**FA**	**341**	0.000	0.000	0.000
**TOU-A1-79**	**FA**	**343**	0.382	0.901	1.069
**TOU-A1-81**	**FA**	**346**	0.000	0.160	0.683
**TOU-A1-82**	**FA**	**347**	0.000	0.102	0.585
**TOU-A1-84**	**FA**	**348**	0.000	0.000	0.078
**TOU-A1-85**	**FA**	**349**	0.003	0.005	0.209
**TOU-A1-93**	**FA**	**355**	0.000	0.014	0.397
**TOU-A1-96**	**FA**	**357**	0.001	0.000	0.000
**TOU-A1-98**	**FA**	**359**	0.001	0.000	0.000
**TOU-C-1**	**FA**	**360**	0.000	0.000	0.627
**TOU-C-2**	**FA**	**361**	0.000	0.000	0.000
**TOU-D-1**	**FA**	**363**	0.000	0.000	0.063
**TOU-D-5**	**FA**	**364**	0.003	0.000	0.004
**TOU-E-11**	**FA**	**366**	0.000	0.527	0.904
**TOU-E-2**	**FA**	**367**	0.007	0.026	0.069
**TOU-E-7**	**FA**	**368**	0.004	0.006	0.650
**TOU-F-1**	**FA**	**369**	0.009	0.199	0.860
**TOU-G-1**	**FA**	**371**	0.000	0.000	0.382
**TOU-H-10**	**FA**	**372**	0.000	0.001	0.000
**TOU-H-13**	**FA**	**374**	0.000	0.155	0.489

**TOU-H-3**	**FA**	**375**	0.000	0.172	0.832
**TOU-I-1**	**FA**	**377**	0.000	0.000	0.699
**TOU-I-17**	**FA**	**378**	0.004	0.027	0.061
**TOU-I-2**	**FA**	**379**	0.000	0.000	0.235
**TOU-I-6**	**FA**	**380**	0.001	0.000	0.000
**TOU-K-2**	**FA**	**385**	0.000	0.000	0.000
**TOU-L-10**	**FA**	**387**	0.000	0.000	0.455
**TOU-L-17**	**FA**	**388**	0.000	0.016	0.139
**TOU-L-5**	**FA**	**389**	0.005	0.016	0.172
**Ts-1**	**WA**	**6970**	0.128	0.626	0.890
**Ts-5**	**WA**	**6971**	0.131	0.396	0.933
**Tsu-0**	**WA**	**7373**	0.002	0.000	0.000
**Tsu-1**	**WA**	**6972**	0.255	0.266	0.297
**Tu-0**	**WA**	**8395**	0.000	0.000	0.000
**Ull1-1**	**WA**	**8426**	0.002	0.117	0.493
**Ull-2-3**	**WA**	**6973**	0.001	0.133	0.143
**Ull-2-5**	**WA**	**6974**	0.003	0.000	0.004
**Uod-1**	**WA**	**6975**	0.005	0.011	0.161
**Uod-7**	**WA**	**6976**	0.000	0.000	0.100
**Van-0**	**WA**	**6977**	0.131	0.271	0.308
**Var-2-1**	**WA**	**7516**	0.003	0.148	0.301
**Var-2-6**	**WA**	**7517**	0.004	0.012	0.057
**Vastervik**	**WA**	**9058**	0.000	0.000	0.000
**Vimmerby**	**WA**	**8249**	0.000	0.337	0.691
**Vinslov**	**WA**	**9057**	0.000	0.259	1.386
**VOU-1**	**FA**	**390**	0.000	0.368	0.860
**VOU-10**	**FA**	**391**	0.495	1.176	1.523
**VOU-2**	**FA**	**392**	0.002	0.017	0.021
**VOU-4**	**FA**	**393**	0.000	0.000	0.435
**VOU-5**	**FA**	**394**	0.000	0.000	0.953
**VOU-6**	**FA**	**395**	0.000	0.249	0.489
**VOU-7**	**FA**	**396**	0.003	0.246	0.754
**Wa-1**	**WA**	**6978**	0.000	0.006	0.181
**Wei-0**	**WA**	**6979**	0.020	0.721	1.604
**Wil-1**	**WA**	**100000**	0.018	0.041	0.075
**Ws-0**	**WA**	**6980**	0.000	0.498	0.871
**Ws-2**	**WA**	**6981**	0.003	0.000	0.004
**Wt-5**	**WA**	**6982**	0.327	0.326	0.319
**Yo-0**	**WA**	**6983**	0.132	0.276	0.694
**Zdr-1**	**WA**	**6984**	0.005	0.032	0.072
**Zdr-6**	**WA**	**6985**	0.001	0.029	0.179
