# Supplementary material for: An Atypical Kinase under Balancing Selection Confers Broad-Spectrum Disease Resistance in Arabidopsis
Source: PLoS Genet. 2013 Sep 12;9(9):e1003766. doi: 10.1371/journal.pgen.1003766 (PMC3772041; doi:10.1371/journal.pgen.1003766)
Supplement: Table S8 — Sequence polymorphisms of the 5,111 bp region centered on RKS1. (PDF) [file pgen.1003766.s024.pdf]

Supplementary Table 8. Sequence polymorphisms of the 5,111 bp region centered on *RKS1* .

Accessions: native name and ecotype ID																									
Amino-acid change				Phe->Leu	Val->Ile	His->Asn	Ile->Leu	Ala->Ser	Leu->Phe	Asp->Val	Thr->Thr	Pro->Pro	Ile->Leu	Ser->Arg					Gly->Asp	Ala->Ser	Ser->Phe	Arg->Lys	Val->Ile	Ser->Ile	Tyr->Phe
Gene position				15	331	397	415	490	543	566	657	675	745	871					920	799	632	569	451	344	224
Nucleotide position		**60**	88	112	428	**494**	512	587	640	663	754	**772**	842	968	1126	1174-1179	1307	**1373**	**1550**	1671	1838	1901	**2019**	2126	2246
		5' *At3g57700*		*At3g57700*											intergenic *At3g57700* - *At3g57710*				*At3g57710* *RKS1*						
Reference (Col-0)		C	G	C	G	C	A	G	A	A	A	T	A	A	C	TAGTAG	G	C	C	C	G	C	C	C	T
Bor-1	5837	.	.	.	.	.	.	.	.	.	.	.	.	.	.	.	.	.	.	.	.	.	.	.	.
Bor-4	6903	.	.	.	.	.	.	.	.	.	.	.	.	.	.	.	.	.	.	.	.	.	.	.	.
CIBC-17	6907	.	.	.	.	.	.	.	.	.	.	.	.	.	.	.	.	.	.	.	.	.	.	.	.
Col-0	6909	.	.	.	.	.	.	.	.	.	.	.	.	.	.	.	.	.	.	.	.	.	.	.	.
N13	7438	.	.	.	.	.	.	.	.	.	.	.	.	.	.	.	.	.	.	.	.	.	.	.	.
Ler-1	6932	.	.	.	.	.	.	.	.	.	.	.	.	.	.	.	.	.	.	.	.	.	.	.	.
Lov-1	6043	.	.	.	.	.	.	.	.	.	.	.	.	.	.	.	.	.	.	.	.	.	.	.	.
Lov-5	6046	.	.	.	.	.	.	.	.	.	.	.	.	.	.	.	.	.	.	.	.	.	.	.	.
Lp2-2	7520	.	.	.	.	.	.	.	.	.	.	.	.	.	.	.	.	.	.	.	.	.	.	A	.
Mt-0	6939	.	.	.	.	.	.	.	.	.	.	.	.	.	.	.	.	.	.	.	.	.	.	.	.
Mz-0	6940	.	.	.	.	.	.	.	.	.	.	.	.	.	.	.	.	.	.	.	.	.	.	.	.
Omo-2-1	7518	.	.	.	.	.	.	.	.	.	.	.	.	.	.	.	.	.	.	.	.	.	.	.	.
Omo-2-3	7519	.	.	.	.	.	.	.	.	.	.	.	.	.	.	.	.	.	.	.	.	.	.	.	.
Pu2-7	6956	.	.	.	.	.	.	.	.	.	.	.	.	.	.	.	.	.	.	.	.	.	.	.	.
Pu2-23	6951	.	.	.	.	.	.	.	.	.	.	.	.	.	.	.	.	.	.	.	.	.	.	.	.
Tamm-2	6968	.	.	.	.	.	.	.	.	.	.	.	.	.	.	.	.	.	.	.	.	.	.	.	.
Tamm-27	6969	.	.	.	.	.	.	.	.	.	.	.	.	.	.	.	.	.	.	.	.	.	.	.	.
Tsu-1	6972	.	.	.	.	.	.	.	.	.	.	.	.	.	.	.	.	.	.	.	.	.	.	.	.
Ull-2-5	6974	.	.	.	.	.	.	.	.	.	.	.	.	.	.	.	.	.	.	.	.	.	.	.	.
Uod-1	6975	.	.	.	.	.	.	.	.	.	.	.	.	.	.	.	.	.	.	.	.	.	.	.	.
Wa-1	6978	.	.	.	.	.	.	.	.	.	.	.	.	.	.	.	.	.	.	.	.	.	.	.	.
Ws-2	6981	.	.	.	.	.	.	.	.	.	.	.	.	.	.	.	.	.	.	.	.	.	.	.	.
Zdr-1	6984	.	.	.	.	.	.	.	.	.	.	.	.	.	.	.	.	.	.	A	.	.	.	.	.
Zdr-6	6985	.	.	.	.	.	.	.	T	.	.	.	.	.	.	.	.	.	.	.	.	.	.	A	.

Accessions: native name and ecotype ID

Br-0	6904	.	.	.	.	.	.	.	.	.	.	C	C	.	.	------	.	.	.	.	.	.	T	.	.
C24	6906	.	.	.	.	.	.	.	.	.	.	C	C	.	.	.	.	.	.	.	.	.	T	.	.
NFA-10	6943	.	.	.	.	.	.	.	.	.	.	C	C	.	.	.	.	.	.	.	.	.	T	.	.
Ag-0	6897	.	.	.	.	.	.	.	.	T	.	C	.	.	.	.	.	.	.	.	.	.	.	.	.
Kno-18	6928	.	.	.	.	.	.	.	.	T	.	C	.	.	.	.	.	.	.	.	.	.	.	.	.
Pro-0	8213	.	.	.	.	.	.	.	.	T	.	C	.	.	.	.	.	.	.	.	.	.	.	.	.
Ra-0	6958	.	.	.	.	.	.	.	.	T	.	C	.	.	.	.	.	.	.	.	.	.	.	.	.
Var-2-1	7516	.	.	.	.	.	.	.	.	T	.	C	.	.	.	.	.	.	.	.	.	.	.	.	.
Var-2-6	7517	.	.	.	.	.	.	.	.	T	.	C	.	.	.	.	.	.	.	.	.	.	.	.	.
Wt-5	6982	.	.	.	.	.	.	.	.	T	.	C	.	.	.	.	.	.	.	.	.	.	.	.	.
Ct-1	6910	.	.	.	.	.	.	.	.	.	.	C	.	.	.	.	.	.	.	.	.	.	.	.	.
Ga-0	6919	.	.	.	.	.	.	.	.	.	.	C	.	.	.	.	.	.	.	.	.	.	.	.	.
Gy-0	8214	.	.	.	.	.	.	.	.	.	.	C	.	.	.	.	.	.	.	.	.	.	.	.	.
LL-0	6933	.	.	.	.	.	.	.	.	.	.	C	.	.	.	.	.	.	.	.	.	.	.	.	.
Ts-5	6971	.	.	.	.	.	.	.	.	.	.	C	.	.	.	.	.	.	.	.	.	.	.	.	.
Est-1	6916	.	.	.	.	.	T	.	.	.	.	C	.	.	.	.	.	.	T	.	.	.	.	.	.
Gu-0	7149	.	.	.	A	.	.	.	.	.	.	C	.	.	.	.	.	.	.	.	.	.	.	.	.
Kno-10	6927	.	.	.	.	.	.	.	.	.	.	C	.	.	.	.	.	.	.	.	.	.	.	.	.
Kz-1	6930	.	.	.	.	.	.	.	.	.	.	C	.	.	.	.	.	.	.	.	.	.	.	.	.
Kz-9	6931	.	.	.	.	.	.	.	.	.	.	C	.	C	.	.	.	.	.	.	.	.	.	.	.
Lp2-6	7521	.	.	A	.	.	.	.	.	.	.	C	.	.	.	.	.	.	T	.	.	.	.	.	.
Mr-0	7522	.	.	.	.	A	.	.	.	.	.	C	.	C	.	.	.	.	.	.	.	.	.	.	.
Mrk-0	6937	.	.	.	.	.	.	.	.	.	.	C	.	.	.	.	.	.	T	.	.	.	.	.	.
Ms-0	6938	.	.	.	.	.	.	.	.	.	.	C	.	.	.	.	.	.	.	.	.	.	.	.	.
Nd-1	6942	.	.	.	.	.	.	T	.	.	.	C	.	C	.	.	.	.	.	.	.	T	.	.	.
Pna-10	7526	.	.	.	.	.	.	.	.	.	.	C	.	.	.	.	.	.	.	.	.	.	.	.	.
Pna-17	7523	.	.	.	.	.	.	.	.	.	.	C	.	.	.	.	.	.	.	.	.	.	.	.	.
RRS-10	7515	.	.	.	.	.	.	.	.	.	.	C	.	.	.	.	.	.	.	.	.	.	.	.	.
Shahdara	6962	.	.	.	.	.	.	.	.	.	.	C	.	C	G	.	.	.	.	.	.	.	.	.	.
Uod-7	6976	.	.	.	.	.	.	.	.	.	.	.	.	.	.	.	.	.	T	.	.	.	.	.	.
Ws-0	6980	.	.	.	.	.	.	.	.	.	.	C	.	.	.	.	.	.	.	.	.	.	.	.	.
Yo-0	6983	.	.	.	.	.	.	.	.	.	.	C	.	.	.	.	.	.	.	.	.	.	.	.	.
Bur-0	6905	.	.	.	.	.	.	.	.	.	.	C	.	.	.	.	.	.	T	.	.	.	.	.	.
CIBC-5	6908	.	.	.	.	.	.	.	.	.	G	C	.	.	.	.	.	.	T	.	.	.	.	.	.
Eden-1	6009	.	A	.	.	.	.	.	.	.	.	C	.	.	.	.	.	.	T	.	.	.	.	.	.
Eden-2	6913	.	A	.	.	.	.	.	.	.	.	C	.	.	.	.	.	.	T	.	.	.	.	.	.
Edi-0	6914	.	.	.	.	.	.	.	.	.	.	C	.	.	.	.	.	.	T	.	.	.	.	.	.
Fab-2	6917	.	.	.	.	.	.	.	.	.	.	C	.	.	.	.	.	.	T	.	.	.	.	.	.
Fab-4	6918	.	.	.	.	.	.	.	.	.	.	C	.	.	.	.	.	.	T	.	.	.	.	.	.

Got-7	6921	.	.	.	.	.	.	.	.	.	.	C	.	.	.	.	.	.	T	.	.	.	.	.	.
Got-22	6920	.	.	.	.	.	.	.	.	.	.	C	.	.	.	.	.	.	T	.	.	.	.	.	.
Nok-3	6945	.	.	.	.	.	.	.	.	.	.	C	.	.	.	.	.	.	T	.	.	.	.	.	.
Oy-0	6946	.	.	.	.	.	.	.	.	.	.	C	.	.	.	.	.	.	T	.	.	.	.	.	.
Spr-1-2	6964	.	.	.	.	.	.	.	.	.	.	C	.	.	.	.	.	.	T	.	.	.	.	.	.
Spr-1-6	6965	.	.	.	.	.	.	.	.	.	.	C	.	.	.	.	.	.	T	.	.	.	.	.	.
Sq-1	6966	.	.	.	.	.	.	.	.	.	G	C	.	.	.	.	.	.	T	.	.	.	.	.	.
Ull-2-3	6973	.	.	.	.	.	.	.	.	.	.	C	.	.	.	.	.	.	T	.	.	.	.	.	.
Cvi-0	6911	.	.	.	.	A	.	.	.	.	.	C	C	.	.	.	T	T	.	.	A	.	.	.	A
Kas-1	na	.	.	.	.	A	.	.	.	.	.	C	C	.	.	.	T	T	.	.	A	.	.	.	.
Kondara	6929	.	.	.	.	A	.	.	.	.	.	C	C	.	.	.	T	T	.	.	A	.	.	.	.
Sorbo	6963	.	.	.	.	A	.	.	.	.	.	C	C	.	.	.	T	T	.	.	A	.	.	.	.
Bay-0	6899	.	.	.	.	A	.	.	.	.	.	C	C	.	.	.	.	T	.	.	A	.	.	.	.
Fei-0	8215	.	.	.	.	A	.	.	.	.	.	C	C	.	.	.	.	T	.	.	A	.	.	.	.
HR-5	6924	.	.	.	.	A	.	.	.	.	.	C	C	.	.	.	.	T	.	.	A	.	.	.	.
HR-10	6923	.	.	.	.	A	.	.	.	.	.	C	C	.	.	.	.	T	.	.	A	.	.	.	.
Kin-0	6926	.	.	.	.	A	.	.	.	.	.	C	C	.	.	.	.	T	.	.	A	.	.	.	.
Lz-0	6936	.	.	.	.	A	.	.	.	.	.	C	C	.	.	.	.	T	.	.	A	.	.	.	.
NFA-8	6944	.	.	.	.	A	.	.	.	.	.	C	C	.	.	.	.	T	.	.	A	.	.	.	.
Ren-1	6959	.	.	.	.	A	.	.	.	.	.	C	C	.	.	.	.	T	.	.	A	.	.	.	.
Ren-11	6960	.	.	.	.	A	.	.	.	.	.	C	C	.	.	.	.	T	.	.	A	.	.	.	.
Rmx-A02	7524	.	.	.	.	A	.	.	.	.	.	C	C	.	.	.	.	T	.	.	A	.	.	.	.
Rmx-A180	7525	.	.	.	.	A	.	.	.	.	.	C	C	.	.	.	.	T	.	.	A	.	.	.	.
RRS-7	7514	.	.	.	.	A	.	.	.	.	.	C	C	.	.	.	.	T	.	.	A	.	.	.	.
Sq-8	6967	.	.	.	.	A	.	.	.	.	.	C	C	.	.	.	.	T	.	.	A	.	.	.	.
An-1	6898	T	.	.	.	A	.	.	.	.	.	C	C	.	.	.	.	T	.	.	A	.	.	.	.
Bil-5	6900	T	.	.	.	A	.	.	.	.	.	C	C	.	.	.	.	T	.	.	A	.	.	.	.
Bil-7	6901	T	.	.	.	A	.	.	.	.	.	C	C	.	.	.	.	T	.	.	A	.	.	.	.
Ei-2	6915	T	.	.	.	A	.	.	.	.	.	C	C	.	.	.	.	T	.	.	A	.	.	.	.
Se-0	6961	T	.	.	.	A	.	.	.	.	.	C	C	.	.	.	.	T	.	.	A	.	.	.	.
Ts-1	6970	T	.	.	.	A	.	.	.	.	.	C	C	.	.	.	.	T	.	.	A	.	.	.	.
Wei-0	6979	T	.	.	.	A	.	.	.	.	.	C	C	.	.	.	.	T	.	.	A	.	.	.	.

Accessions: native name and ecotype ID

Grey cells: *At3g57700* ; blue cells: *At3g57710* (*RKS1*); yellow cells: *At3g57720* ; green cells: intergenic regions; orange cells: indels.

Nucleotide positions in bold indicate SNPs which are part of the set of 214,051 SNPs.

Ecotype ID retrieved from 1,307 natural accessions genotyped using a 250K SNP chip (ref. 16). na: not available.

Amino-acid change		Ser->Ser	Ala->Ala	Val->Val	Arg->Ser	Ser->Asn	Leu->Leu	Ala->Ala	Arg->Arg	Gly->Gly	Ser->Tyr	Gln->STOP														
Gene position		186	168	105	96	83	72	57	39	27	23	10														
Nucleotide position		2284	2302	2365	2374	**2387**	2398	**2413**	2431	2443	2447	2460	2475	2481	2487	2490	2495	2504	2516	2541-2542	2547-2563	2565-2566	2584	2587-2588	2598	2601-2602
		At3g57710 RKS1											intergenic *At3g57710 - At3g57720*													
Reference (Col-0)		A	G	T	T	C	C	C	C	T	G	G	C	C	T	C	T	C	A	CT	AAGTGTCAAAAGCCATT	CT	C	TC	C	TG
**Accessions: native name and ecotype ID**																										
Bor-1	5837	.	.	.	.	.	.	.	.	.	.	.	.	.	.	.	.	.	.	.	.	.	.	.	.	.
Bor-4	6903	.	.	.	.	.	.	.	.	.	.	.	.	.	.	.	.	.	.	.	.	.	.	.	.	.
CIBC-17	6907	.	.	.	.	.	.	.	.	.	.	.	.	.	.	.	.	.	.	.	.	.	.	.	.	.
Col-0	6909	.	.	.	.	.	.	.	.	.	.	.	.	.	.	.	.	.	.	.	.	.	.	.	.	.
N13	7438	.	.	.	.	.	.	.	.	.	.	.	.	.	.	.	.	.	.	.	.	.	.	.	.	.
Ler-1	6932	.	.	.	.	.	.	.	.	.	.	.	.	.	.	.	.	.	.	.	.	.	.	.	.	.
Lov-1	6043	.	.	.	.	.	.	.	.	.	.	.	.	.	.	.	.	.	.	.	.	.	.	.	.	.
Lov-5	6046	.	.	.	.	.	.	.	.	.	.	.	.	.	.	.	.	.	.	.	.	.	.	.	.	.
Lp2-2	7520	.	.	.	.	.	.	.	.	.	.	.	.	.	.	.	.	.	.	.	.	.	.	.	.	.
Mt-0	6939	.	.	.	.	.	.	.	.	.	.	.	.	.	.	.	.	.	.	.	.	.	.	.	.	.
Mz-0	6940	.	.	.	.	.	.	.	.	.	.	.	.	.	.	.	.	.	.	.	.	.	.	.	.	.
Omo-2-1	7518	.	.	.	.	.	.	.	.	.	.	.	.	.	.	.	.	.	.	.	.	.	.	.	.	.
Omo-2-3	7519	.	.	.	.	.	.	.	.	.	.	.	.	.	.	.	.	.	.	.	.	.	.	.	.	.
Pu2-7	6956	.	.	.	.	.	.	.	.	.	.	.	.	.	.	.	.	.	.	.	.	.	.	.	.	.
Pu2-23	6951	.	.	.	.	.	.	.	.	.	.	.	.	.	.	.	.	.	.	.	.	.	.	.	.	.
Tamm-2	6968	.	.	.	.	.	.	.	.	.	.	.	.	.	.	.	.	.	.	.	.	.	.	.	.	.
Tamm-27	6969	.	.	.	.	.	.	.	.	.	.	.	.	.	.	.	.	.	.	.	.	.	.	.	.	.
Tsu-1	6972	.	.	.	.	.	.	.	.	.	.	.	.	.	.	.	.	.	.	.	.	.	.	.	.	.
Ull-2-5	6974	.	.	.	.	.	.	.	.	.	.	.	.	.	.	.	.	.	.	.	.	.	.	.	.	.
Uod-1	6975	.	.	.	.	.	.	.	.	.	.	.	.	.	.	.	.	.	.	.	.	.	.	.	.	.
Wa-1	6978	.	.	.	.	.	.	.	.	.	.	.	.	.	.	.	.	.	.	.	.	.	.	.	.	.
Ws-2	6981	.	.	.	.	.	.	.	.	.	.	.	.	.	.	.	.	.	.	.	.	.	.	.	.	.
Zdr-1	6984	.	.	.	.	.	.	.	.	.	.	.	.	.	.	.	.	.	.	.	.	.	.	.	.	.
Zdr-6	6985	.	.	.	.	.	.	.	.	.	.	.	.	.	.	.	.	.	.	.	.	.	.	.	.	.

Accessions: native name and ecotype ID

Br-0	6904	.	.	.	.	.	.	.	.	.	.	.	.	.	.	.	.	.	.	.	.	.	.	.	.	.
C24	6906	.	.	.	.	.	.	.	.	.	.	.	.	.	.	.	.	.	.	.	.	.	.	.	.	.
NFA-10	6943	.	.	.	.	.	.	.	.	.	.	.	.	.	.	.	.	.	.	.	.	.	.	.	.	.
Ag-0	6897	.	.	.	.	.	.	.	.	.	.	.	.	.	.	.	.	.	.	.	.	.	.	.	.	.
Kno-18	6928	.	.	.	.	.	.	.	.	.	.	.	.	.	.	.	.	.	.	.	.	.	.	.	.	.
Pro-0	8213	.	.	.	.	.	.	.	.	.	.	.	.	.	.	.	.	.	.	.	.	.	.	.	.	.
Ra-0	6958	.	.	.	.	.	.	.	.	.	.	.	.	.	.	.	.	.	.	.	.	.	.	.	.	.
Var-2-1	7516	.	.	.	.	.	.	.	.	.	.	.	.	.	.	.	.	.	.	.	.	.	.	.	.	.
Var-2-6	7517	.	.	.	.	.	.	.	.	.	.	.	.	.	.	.	.	.	.	.	.	.	.	.	.	.
Wt-5	6982	.	.	.	.	.	.	.	.	.	.	.	.	.	.	.	.	.	.	.	.	.	.	.	.	.
Ct-1	6910	T	A	A	A	T	T	T	T	C	.	.	.	T	A	T	.	T	.	--	-----------------	TC	G	--	T	AA
Ga-0	6919	T	A	A	A	T	T	T	T	C	.	.	.	T	A	T	.	T	.	--	-----------------	TC	G	--	T	AA
Gy-0	8214	T	A	A	A	T	T	T	T	C	.	.	.	T	A	T	.	T	.	--	-----------------	TC	G	--	T	AA
LL-0	6933	T	A	A	A	T	T	T	T	C	.	.	.	T	A	T	.	T	.	--	-----------------	TC	G	--	T	AA
Ts-5	6971	T	A	A	A	T	T	T	T	C	.	.	.	T	A	T	.	T	.	--	-----------------	TC	G	--	T	AA
Est-1	6916	T	A	A	A	T	T	T	T	C	.	.	.	T	A	T	.	T	.	--	-----------------	TC	G	--	T	AA
Gu-0	7149	T	A	A	A	T	T	T	T	C	.	.	.	T	A	T	.	T	.	--	-----------------	TC	G	--	T	AA
Kno-10	6927	T	A	A	A	T	T	T	T	C	.	.	.	T	A	T	.	T	.	--	-----------------	TC	G	--	T	AA
Kz-1	6930	T	A	A	A	T	T	T	T	C	.	.	.	T	A	T	.	T	.	--	-----------------	TC	G	--	T	AA
Kz-9	6931	T	A	A	A	T	T	T	T	C	.	.	.	T	A	T	.	T	.	--	-----------------	TC	G	--	T	AA
Lp2-6	7521	T	A	A	A	T	T	T	T	C	.	.	.	T	A	T	.	T	.	--	-----------------	TC	G	--	T	AA
Mr-0	7522	T	A	A	A	T	T	T	T	C	.	.	.	T	A	T	.	T	.	--	-----------------	TC	G	--	T	AA
Mrk-0	6937	T	A	A	A	T	T	T	T	C	.	.	.	T	A	T	.	T	.	--	-----------------	TC	G	--	T	AA
Ms-0	6938	T	A	A	A	T	T	T	T	C	.	.	.	T	A	T	C	T	.	--	-----------------	TC	G	--	T	AA
Nd-1	6942	T	A	A	A	T	T	T	T	C	.	.	.	T	A	T	.	T	G	--	-----------------	TC	G	--	T	AA
Pna-10	7526	T	A	A	A	T	T	T	T	C	.	.	.	T	A	T	.	T	.	--	-----------------	TC	G	--	T	AA
Pna-17	7523	T	A	A	A	T	T	T	T	C	.	.	.	T	A	T	.	T	.	--	-----------------	TC	G	--	T	AA
RRS-10	7515	T	A	A	A	T	T	T	T	C	.	.	.	T	A	T	.	T	.	--	-----------------	TC	G	--	T	AA
Shahdara	6962	T	A	A	A	T	T	T	T	C	.	.	.	T	A	T	.	T	.	--	-----------------	TC	G	--	T	AA
Uod-7	6976	T	A	A	A	T	T	T	T	C	.	.	.	T	A	T	.	T	.	--	-----------------	TC	G	--	T	AA
Ws-0	6980	T	A	A	A	T	T	T	T	C	.	.	.	T	A	T	C	T	.	--	-----------------	TC	G	--	T	AA
Yo-0	6983	T	A	A	A	T	T	T	T	C	.	.	.	T	A	T	.	T	.	--	-----------------	TC	G	--	T	AA
Bur-0	6905	T	A	A	A	T	T	T	T	C	.	.	.	T	A	T	.	T	.	--	-----------------	TC	G	--	T	AA
CIBC-5	6908	T	A	A	A	T	T	T	T	C	.	.	.	T	A	T	.	T	.	--	-----------------	TC	G	--	T	AA
Eden-1	6009	T	A	A	A	T	T	T	T	C	.	.	.	T	A	T	.	T	.	--	-----------------	TC	G	--	T	AA
Eden-2	6913	T	A	A	A	T	T	T	T	C	.	.	.	T	A	T	.	T	.	--	-----------------	TC	G	--	T	AA
Edi-0	6914	T	A	A	A	T	T	T	T	C	.	.	.	T	A	T	.	T	.	--	-----------------	TC	G	--	T	AA
Fab-2	6917	T	A	A	A	T	T	T	T	C	.	.	.	T	A	T	.	T	.	--	-----------------	TC	G	--	T	AA
Fab-4	6918	T	A	A	A	T	T	T	T	C	.	.	.	T	A	T	.	T	.	--	-----------------	TC	G	--	T	AA

Accessions: native name and ecotype ID

Got-7	6921	T	A	A	A	T	T	T	T	C	.	.	.	T	A	T	.	T	.	--	-----------------	TC	G	--	T	AA
Got-22	6920	T	A	A	A	T	T	T	T	C	.	.	.	T	A	T	.	T	.	--	-----------------	TC	G	--	T	AA
Nok-3	6945	T	A	A	A	T	T	T	T	C	.	.	.	T	A	T	.	T	.	--	-----------------	TC	G	--	T	AA
Oy-0	6946	T	A	A	A	T	T	T	T	C	.	.	.	T	A	T	.	T	.	--	-----------------	TC	G	--	T	AA
Spr-1-2	6964	T	A	A	A	T	T	T	T	C	.	.	.	T	A	T	.	T	.	--	-----------------	TC	G	--	T	AA
Spr-1-6	6965	T	A	A	A	T	T	T	T	C	.	.	.	T	A	T	.	T	.	--	-----------------	TC	G	--	T	AA
Sq-1	6966	T	A	A	A	T	T	T	T	C	.	.	.	T	A	T	.	T	.	--	-----------------	TC	G	--	T	AA
Ull-2-3	6973	T	A	A	A	T	T	T	T	C	.	.	.	T	A	T	.	T	.	--	-----------------	TC	G	--	T	AA
Cvi-0	6911	.	.	.	.	.	.	.	.	.	.	.	.	.	.	.	.	.	.	.	.	.	.	.	.	.
Kas-1	na	.	.	.	.	.	.	.	.	.	.	.	.	.	.	.	.	.	.	.	.	.	.	.	.	.
Kondara	6929	.	.	.	.	.	.	.	.	.	.	.	.	.	.	.	.	.	.	.	.	.	.	.	.	.
Sorbo	6963	.	.	.	.	.	.	.	.	.	.	.	.	.	.	.	.	.	.	.	.	.	.	.	.	.
Bay-0	6899	.	.	.	.	.	.	.	.	.	.	A	.	.	.	.	.	.	.	.	.	.	.	.	.	.
Fei-0	8215	.	.	.	.	.	.	.	.	.	.	A	.	.	.	.	.	.	.	.	.	.	.	.	.	.
HR-5	6924	.	.	.	.	.	.	.	.	.	.	A	.	.	.	.	.	.	.	.	.	.	.	.	.	.
HR-10	6923	.	.	.	.	.	.	.	.	.	.	A	.	.	.	.	.	.	.	.	.	.	.	.	.	.
Kin-0	6926	.	.	.	.	.	.	.	.	.	.	A	.	.	.	.	.	.	.	.	.	.	.	.	.	.
Lz-0	6936	.	.	.	.	.	.	.	.	.	.	A	.	.	.	.	.	.	.	.	.	.	.	.	.	.
NFA-8	6944	.	.	.	.	.	.	.	.	.	.	A	.	.	.	.	.	.	.	.	.	.	.	.	.	.
Ren-1	6959	.	.	.	.	.	.	.	.	.	.	A	.	.	.	.	.	.	.	.	.	.	.	.	.	.
Ren-11	6960	.	.	.	.	.	.	.	.	.	.	A	.	.	.	.	.	.	.	.	.	.	.	.	.	.
Rmx-A02	7524	.	.	.	.	.	.	.	.	.	.	A	.	.	.	.	.	.	.	.	.	.	.	.	.	.
Rmx-A180	7525	.	.	.	.	.	.	.	.	.	.	A	.	.	.	.	.	.	.	.	.	.	.	.	.	.
RRS-7	7514	.	.	.	.	.	.	.	.	.	.	A	.	.	.	.	.	.	.	.	.	.	.	.	.	.
Sq-8	6967	.	.	.	.	.	.	.	.	.	.	A	.	.	.	.	.	.	.	.	.	.	.	.	.	.
An-1	6898	T	A	A	A	T	T	T	T	C	.	.	.	T	A	T	.	T	.	--	-----------------	TC	G	--	T	AA
Bil-5	6900	T	A	A	A	T	T	T	T	C	T	.	A	T	A	T	.	T	.	--	-----------------	TC	G	--	T	AA
Bil-7	6901	T	A	A	A	T	T	T	T	C	T	.	A	T	A	T	.	T	.	--	-----------------	TC	G	--	T	AA
Ei-2	6915	T	A	A	A	T	T	T	T	C	.	.	.	T	A	T	.	T	.	--	-----------------	TC	G	--	T	AA
Se-0	6961	T	A	A	A	T	T	T	T	C	.	.	.	T	A	T	.	T	.	--	-----------------	TC	G	--	T	AA
Ts-1	6970	T	A	A	A	T	T	T	T	C	.	.	.	T	A	T	.	T	.	--	-----------------	TC	G	--	T	AA
Wei-0	6979	T	A	A	A	T	T	T	T	C	.	.	.	T	A	T	.	T	.	--	-----------------	TC	G	--	T	AA

Grey cells: *At3g57700* ; blue cells: *At3g57710* (*RKS1*); yellow cells: *At3g57720* ; green cells: intergenic regions; orange cells: indels.

Nucleotide positions in bold indicate SNPs which are part of the set of 214,051 SNPs.

Ecotype ID retrieved from 1,307 natural accessions genotyped using a 250K SNP chip (ref. 16). na: not available.

Accessions: native name and ecotype ID																															
Amino-acid change																						Lys->Lys	Ser->Tyr	Gly-> STOP	Met->Val	Pro->Ala	Ala->Ala	Cys->Tyr	Ala->Ala	Leu->Met
Gene position																						48	269	520-521	745	811	816	962	975	991
Nucleotide position		2602	2604	2609	2631	2634	2640	2643	2648	2656	2658-2659	2673-2676	2680	2682	2692	2699	2725	2828	2848	2852	2853	2867	3003	3224	3475-3476	3700	3766	3771	3917	3930	3946
		intergenic *At3g57710* - *At3g57720*																					*At3g57720*								
Reference (Col-0)		--	G	A	A	C	T	A	C	A	AG	AACT	A	C	G	-	A	G	T	A	A	G	A	C	GG	A	C	A	G	G	T
Bor-1	5837	.	.	.	.	.	.	.	.	.	.	.	.	.	.	.	.	.	.	.	.	.	.	.	.	.	.	.	.	.	.
Bor-4	6903	.	.	.	.	.	.	.	.	.	.	.	.	.	.	.	.	.	.	.	.	.	.	.	.	.	.	.	.	.	.
CIBC-17	6907	.	.	.	.	.	.	.	.	.	.	.	.	.	.	.	.	.	.	.	.	.	.	.	.	.	.	.	.	.	.
Col-0	6909	.	.	.	.	.	.	.	.	.	.	.	.	.	.	.	.	.	.	.	.	.	.	.	.	.	.	.	.	.	.
N13	7438	.	.	.	.	.	.	.	.	.	.	.	.	.	.	.	.	.	.	.	.	.	.	.	.	.	.	.	.	.	.
Ler-1	6932	.	.	.	.	.	.	.	.	.	.	.	.	.	.	.	.	.	.	.	.	.	.	.	.	.	.	.	.	.	.
Lov-1	6043	.	.	.	.	.	.	.	.	.	.	.	.	.	.	.	.	.	.	.	.	.	.	.	.	.	.	.	.	.	.
Lov-5	6046	.	.	.	.	.	.	.	.	.	.	.	.	.	.	.	.	.	.	.	.	.	.	.	.	.	.	.	.	.	.
Lp2-2	7520	.	.	.	.	.	.	.	.	.	.	.	.	.	.	.	.	.	.	.	.	.	.	.	.	.	.	.	.	.	.
Mt-0	6939	.	.	.	.	.	.	.	.	.	.	.	.	.	.	.	.	.	.	.	.	.	.	.	.	.	.	.	.	.	.
Mz-0	6940	.	.	.	.	.	.	.	.	.	.	.	.	.	.	.	.	.	.	.	.	.	.	.	.	.	.	.	.	.	.
Omo-2-1	7518	.	.	.	.	.	.	.	.	.	.	.	.	.	.	.	.	.	.	.	.	.	.	.	.	.	.	.	.	.	.
Omo-2-3	7519	.	.	.	.	.	.	.	.	.	.	.	.	.	.	.	.	.	.	.	.	.	.	.	.	.	.	.	.	.	.
Pu2-7	6956	.	.	.	.	.	.	.	.	.	.	.	.	.	.	.	.	.	.	.	.	.	.	.	.	.	.	.	.	.	.
Pu2-23	6951	.	.	.	.	.	.	.	.	.	.	.	.	.	.	.	.	.	.	.	.	.	.	.	.	.	.	.	.	.	.
Tamm-2	6968	.	.	.	.	.	.	.	.	.	.	.	.	.	.	.	.	.	.	.	.	.	.	.	.	.	.	.	.	.	.
Tamm-27	6969	.	.	.	.	.	.	.	.	.	.	.	.	.	.	.	.	.	.	.	.	.	.	.	.	.	.	.	.	.	.
Tsu-1	6972	.	.	.	.	.	.	.	.	.	.	.	.	.	.	.	.	.	.	.	.	.	.	.	.	.	.	.	.	.	.
Ull-2-5	6974	.	.	.	.	.	.	.	.	.	.	.	.	.	.	.	.	.	.	.	.	.	.	.	.	.	.	.	.	.	.
Uod-1	6975	.	.	.	.	.	.	.	.	.	.	.	.	.	.	.	.	.	.	.	.	.	.	.	.	.	.	.	.	.	.
Wa-1	6978	.	.	.	.	.	.	.	.	.	.	.	.	.	.	.	.	.	.	.	.	.	.	.	.	.	.	.	.	.	.
Ws-2	6981	.	.	.	.	.	.	.	.	.	.	.	.	.	.	.	.	.	.	.	.	.	.	.	.	.	.	.	.	.	.
Zdr-1	6984	.	.	.	.	.	.	.	.	.	.	.	.	.	.	.	.	.	.	.	.	.	.	.	.	.	.	.	.	.	.
Zdr-6	6985	.	.	.	.	.	.	.	.	.	.	.	.	.	.	.	.	.	.	.	.	.	.	.	.	.	.	.	.	.	.

Accessions: native name and ecotype ID

Br-0	6904	.	.	.	.	.	.	.	.	.	.	.	.	.	C	.	.	.	.	.	.	.	.	.	.	.	.	.	.	.	.
C24	6906	.	.	.	.	.	.	.	.	.	.	.	.	.	C	.	.	.	.	.	.	.	.	.	.	.	.	.	.	.	.
NFA-10	6943	.	.	.	.	.	.	.	.	.	.	.	.	.	C	.	.	T	.	.	.	.	.	.	--	.	.	.	.	.	.
Ag-0	6897	.	.	.	.	.	.	.	.	.	.	.	.	.	.	.	.	.	.	.	.	.	.	.	.	.	.	.	.	.	.
Kno-18	6928	.	.	.	.	.	.	.	.	.	.	.	.	.	.	.	.	.	.	.	.	.	.	.	.	.	.	.	.	.	.
Pro-0	8213	.	.	.	.	.	.	.	.	.	.	.	.	.	.	.	.	.	.	.	.	.	.	.	.	.	.	.	.	.	.
Ra-0	6958	.	.	.	.	.	.	.	.	.	.	.	.	.	.	.	.	.	.	.	.	.	.	.	.	.	.	.	.	.	.
Var-2-1	7516	.	.	.	.	.	.	.	.	.	.	.	.	.	.	.	.	.	.	.	.	.	.	.	.	.	.	.	.	.	.
Var-2-6	7517	.	.	.	.	.	.	.	.	.	.	.	.	.	.	.	.	.	.	.	.	.	.	.	.	.	.	.	.	.	.
Wt-5	6982	.	.	.	.	.	.	.	.	.	.	.	.	.	.	.	.	.	.	.	.	.	.	.	.	.	.	.	.	.	.
Ct-1	6910	GC	A	G	T	G	C	G	A	C	GA	----	C	A	.	.	.	.	C	.	.	T	.	.	.	.	.	.	.	.	.
Ga-0	6919	GC	A	G	T	G	C	G	A	C	GA	----	C	A	.	.	.	.	C	.	.	T	.	.	.	.	.	.	.	.	.
Gy-0	8214	GC	A	G	T	G	C	G	A	C	GA	----	C	A	.	.	.	.	C	.	.	T	.	.	.	.	.	.	.	.	.
LL-0	6933	GC	A	G	T	G	C	G	A	C	GA	----	C	A	.	.	.	.	C	.	.	T	.	.	.	.	.	.	.	.	.
Ts-5	6971	GC	A	G	T	G	C	G	A	C	GA	----	C	A	.	.	.	.	C	.	.	T	.	.	.	.	.	.	.	.	.
Est-1	6916	GC	A	G	T	G	C	G	A	C	GA	----	C	A	.	.	.	.	C	.	.	T	G	A	.	.	.	T	A	.	.
Gu-0	7149	GC	A	G	T	G	C	G	A	C	GA	----	C	A	.	.	.	.	C	.	.	T	G	A	.	.	.	T	A	.	.
Kno-10	6927	GC	A	G	T	G	C	G	A	C	GA	----	C	A	.	.	.	.	C	.	.	T	G	A	.	.	.	T	A	.	.
Kz-1	6930	GC	A	G	T	G	C	G	A	C	GA	----	C	A	.	.	.	.	C	.	.	T	G	A	.	.	.	T	A	.	.
Kz-9	6931	GC	A	G	T	G	C	G	A	C	GA	----	C	A	.	.	.	.	C	.	.	T	G	A	.	.	.	T	A	.	.
Lp2-6	7521	GC	A	G	T	G	C	G	A	C	GA	----	C	A	.	.	.	.	C	.	.	T	G	A	.	.	.	T	A	.	.
Mr-0	7522	GC	A	G	T	G	C	G	A	C	GA	----	C	A	.	.	.	.	C	.	.	T	G	A	.	G	.	T	A	.	.
Mrk-0	6937	GC	A	G	T	G	C	G	A	C	GA	----	C	A	.	.	.	.	C	.	.	T	G	A	.	.	.	T	A	.	.
Ms-0	6938	GC	A	G	T	G	C	G	A	C	GA	----	C	A	.	.	.	.	C	.	.	T	G	A	.	.	.	T	A	.	.
Nd-1	6942	GC	A	G	T	G	C	G	A	C	GA	----	C	A	.	.	.	.	C	.	.	T	G	A	.	.	.	T	A	.	.
Pna-10	7526	GC	A	G	T	G	C	G	A	C	GA	----	C	A	.	.	.	.	C	.	.	T	G	A	.	.	.	T	A	.	.
Pna-17	7523	GC	A	G	T	G	C	G	A	C	GA	----	C	A	.	.	.	.	C	.	.	T	G	A	.	.	.	T	A	.	.
RRS-10	7515	GC	A	G	T	G	C	G	A	C	GA	----	C	A	.	.	.	.	C	.	.	T	G	A	.	.	.	T	A	.	.
Shahdara	6962	GC	A	G	T	G	C	G	A	C	GA	----	C	A	.	.	.	.	C	.	.	T	G	A	.	.	.	T	A	.	.
Uod-7	6976	GC	A	G	T	G	C	G	A	C	GA	----	C	A	.	.	.	.	C	.	.	T	G	A	.	.	.	T	A	.	.
Ws-0	6980	GC	A	G	T	G	C	G	A	C	GA	----	C	A	.	.	.	.	C	.	.	T	G	A	.	.	.	T	A	.	.
Yo-0	6983	GC	A	G	T	G	C	G	A	C	GA	----	C	A	.	.	.	.	C	.	.	T	G	A	.	.	.	T	A	.	.
Bur-0	6905	GC	A	G	T	G	C	G	A	C	GA	----	C	A	.	.	.	.	C	.	.	T	G	A	.	.	.	T	A	.	.
CIBC-5	6908	GC	A	G	T	G	C	G	A	C	GA	----	C	A	.	.	.	.	C	.	.	T	G	A	.	.	.	T	A	.	.
Eden-1	6009	GC	A	G	T	G	C	G	A	C	GA	----	C	A	.	.	.	.	C	.	.	T	G	A	.	.	.	T	A	.	.
Eden-2	6913	GC	A	G	T	G	C	G	A	C	GA	----	C	A	.	.	.	.	C	.	.	T	G	A	.	.	.	T	A	.	.
Edi-0	6914	GC	A	G	T	G	C	G	A	C	GA	----	C	A	.	.	.	.	C	.	.	T	G	A	.	.	.	T	A	.	.
Fab-2	6917	GC	A	G	T	G	C	G	A	C	GA	----	C	A	.	.	.	.	C	.	.	T	G	A	.	.	.	T	A	.	A
Fab-4	6918	GC	A	G	T	G	C	G	A	C	GA	----	C	A	.	.	.	.	C	.	.	T	G	A	.	.	.	T	A	.	A

Accessions: native name and ecotype ID

Got-7	6921	GC	A	G	T	G	C	G	A	C	GA	----	C	A	.	.	.	.	C	.	.	T	G	A	.	.	.	T	A	.	.
Got-22	6920	GC	A	G	T	G	C	G	A	C	GA	----	C	A	.	.	.	.	C	.	.	T	G	A	.	.	.	T	A	.	.
Nok-3	6945	GC	A	G	T	G	C	G	A	C	GA	----	C	A	.	.	.	.	C	.	.	T	G	A	.	.	.	T	A	.	.
Oy-0	6946	GC	A	G	T	G	C	G	A	C	GA	----	C	A	.	.	.	.	C	.	.	T	G	A	.	.	.	T	A	.	.
Spr-1-2	6964	GC	A	G	T	G	C	G	A	C	GA	----	C	A	.	.	.	.	C	.	.	T	G	A	.	.	.	T	A	.	.
Spr-1-6	6965	GC	A	G	T	G	C	G	A	C	GA	----	C	A	.	.	.	.	C	.	.	T	G	A	.	.	.	T	A	.	.
Sq-1	6966	GC	A	G	T	G	C	G	A	C	GA	----	C	A	.	.	.	.	C	.	.	T	G	A	.	.	.	T	A	.	.
Ull-2-3	6973	GC	A	G	T	G	C	G	A	C	GA	----	C	A	.	.	.	.	C	.	.	T	G	A	.	.	.	T	A	.	.
Cvi-0	6911	.	.	.	.	.	.	.	.	.	.	.	.	.	.	.	.	.	.	.	.	.	.	.	.	.	.	.	.	C	.
Kas-1	na	.	.	.	.	.	.	.	.	.	.	.	.	.	.	.	.	.	.	-	T	.	.	.	.	.	.	.	.	.	.
Kondara	6929	.	.	.	.	.	.	.	.	.	.	.	.	.	.	.	.	.	.	-	T	.	.	.	.	.	.	.	.	.	.
Sorbo	6963	.	.	.	.	.	.	.	.	.	.	.	.	.	.	.	.	.	.	-	T	.	.	.	.	.	.	.	.	.	.
Bay-0	6899	.	.	.	.	.	.	.	.	.	.	.	.	.	.	.	G	.	.	.	.	.	.	.	.	.	G	.	.	.	.
Fei-0	8215	.	.	.	.	.	.	.	.	.	.	.	.	.	.	.	G	.	.	.	.	.	.	.	.	.	G	.	.	.	.
HR-5	6924	.	.	.	.	.	.	.	.	.	.	.	.	.	.	.	G	.	.	.	.	.	.	.	.	.	G	.	.	.	.
HR-10	6923	.	.	.	.	.	.	.	.	.	.	.	.	.	.	.	G	.	.	.	.	.	.	.	.	.	G	.	.	.	.
Kin-0	6926	.	.	.	.	.	.	.	.	.	.	.	.	.	.	.	G	.	.	.	.	.	.	.	.	.	G	.	.	.	.
Lz-0	6936	.	.	.	.	.	.	.	.	.	.	.	.	.	.	.	G	.	.	.	.	.	.	.	.	.	G	.	.	.	.
NFA-8	6944	.	.	.	.	.	.	.	.	.	.	.	.	.	.	A	G	.	.	.	.	.	.	.	.	.	G	.	.	.	.
Ren-1	6959	.	.	.	.	.	.	.	.	.	.	.	.	.	.	.	G	.	.	.	.	.	.	.	.	.	G	.	.	.	.
Ren-11	6960	.	.	.	.	.	.	.	.	.	.	.	.	.	.	.	G	.	.	.	.	.	.	.	.	.	G	.	.	.	.
Rmx-A02	7524	.	.	.	.	.	.	.	.	.	.	.	.	.	.	.	G	.	.	.	.	.	.	.	.	.	G	.	.	.	.
Rmx-A180	7525	.	.	.	.	.	.	.	.	.	.	.	.	.	.	.	G	.	.	.	.	.	.	.	.	.	G	.	.	.	.
RRS-7	7514	.	.	.	.	.	.	.	.	.	.	.	.	.	.	.	G	.	.	.	.	.	.	.	.	.	G	.	.	.	.
Sq-8	6967	.	.	.	.	.	.	.	.	.	.	.	.	.	.	.	G	.	.	.	.	.	.	.	.	.	G	.	.	.	.
An-1	6898	GC	A	G	T	G	C	G	A	C	GA	----	C	A	.	.	.	.	C	.	.	T	G	A	.	.	.	T	A	.	.
Bil-5	6900	GC	A	G	T	G	C	G	A	C	GA	----	C	A	.	.	.	.	C	.	.	T	G	A	.	.	.	T	A	.	.
Bil-7	6901	GC	A	G	T	G	C	G	A	C	GA	----	C	A	.	.	.	.	C	.	.	T	G	A	.	.	.	T	A	.	.
Ei-2	6915	GC	A	G	T	G	C	G	A	C	GA	----	C	A	.	.	.	.	C	.	.	T	G	A	.	.	.	T	A	.	.
Se-0	6961	GC	A	G	T	G	C	G	A	C	GA	----	C	A	.	.	.	.	C	.	.	T	G	A	.	.	.	T	A	.	.
Ts-1	6970	GC	A	G	T	G	C	G	A	C	GA	----	C	A	.	.	.	.	C	.	.	T	G	A	.	.	.	T	A	.	.
Wei-0	6979	GC	A	G	T	G	C	G	A	C	GA	----	C	A	.	.	.	.	C	.	.	T	G	A	.	.	.	T	A	.	.

Grey cells: *At3g57700* ; blue cells: *At3g57710* (*RKS1*); yellow cells: *At3g57720* ; green cells: intergenic regions; orange cells: indels.

Nucleotide positions in bold indicate SNPs which are part of the set of 214,051 SNPs.

Ecotype ID retrieved from 1,307 natural accessions genotyped using a 250K SNP chip (ref. 16). na: not available.

Accessions: native name and ecotype ID

		4028	**4030**	4107	4119	4127	**4129**	4136	4161	**4266**	4413	4444	4614	4621	4630	4740	4871	4873-4874	4995	**4999**	5053
Amino-acid change																					
Gene position																					
Nucleotide position		4028	**4030**	4107	4119	4127	**4129**	4136	4161	**4266**	4413	4444	4614	4621	4630	4740	4871	4873-4874	4995	**4999**	5053
		intergenic *At3g57720* - *At3g57730*																			
Reference (Col-0)		T	C	G	G	G	A	T	C	T	A	A	T	C	G	G	A	CT	A	T	G
Bor-1	5837	.	.	.	.	.	.	.	.	.	.	.	.	.	.	.	.	.	.	.	.
Bor-4	6903	.	.	.	.	.	.	.	.	.	.	.	.	.	.	.	.	.	.	.	.
CIBC-17	6907	.	.	.	.	.	.	.	.	.	.	.	.	.	.	.	.	.	.	.	.
Col-0	6909	.	.	.	.	.	.	.	.	.	.	.	.	.	.	.	.	.	.	.	.
N13	7438	.	.	.	.	.	.	.	.	.	.	.	.	.	.	.	.	.	.	.	.
Ler-1	6932	.	.	.	.	.	.	.	.	.	.	.	.	.	.	.	.	.	.	.	.
Lov-1	6043	.	.	.	.	.	.	.	.	.	.	.	.	.	.	.	.	.	.	.	.
Lov-5	6046	.	.	.	.	.	.	.	.	.	.	.	.	.	.	.	.	.	.	.	.
Lp2-2	7520	.	.	.	.	.	.	.	.	.	.	.	.	.	.	.	.	.	.	.	.
Mt-0	6939	.	.	.	.	.	.	.	.	.	.	.	.	.	.	.	.	.	.	.	.
Mz-0	6940	.	.	.	.	.	.	.	.	.	.	.	.	.	.	.	.	.	.	.	.
Omo-2-1	7518	.	.	.	.	.	.	.	.	.	.	.	.	.	.	.	.	.	.	.	.
Omo-2-3	7519	.	.	.	.	.	.	.	.	.	.	.	.	.	.	.	.	.	.	.	.
Pu2-7	6956	.	.	.	.	.	.	.	.	.	.	.	.	.	.	.	.	.	.	.	.
Pu2-23	6951	.	.	.	.	.	.	.	.	.	.	.	.	.	.	.	.	.	.	.	.
Tamm-2	6968	.	.	.	.	.	.	.	.	.	.	.	.	.	.	.	.	.	.	.	.
Tamm-27	6969	.	.	.	.	.	.	.	.	.	.	.	.	.	.	.	.	.	.	.	.
Tsu-1	6972	.	.	.	.	.	.	.	.	.	.	.	.	.	.	.	.	.	.	.	.
Ull-2-5	6974	.	.	.	.	.	.	.	.	.	.	.	.	.	.	.	.	.	.	.	.
Uod-1	6975	.	.	.	.	.	.	.	.	.	.	.	.	.	.	.	.	.	.	.	.
Wa-1	6978	.	.	.	.	.	.	.	.	.	.	.	.	.	.	.	.	.	.	.	.
Ws-2	6981	.	.	.	.	.	.	.	.	.	.	.	.	.	.	.	.	.	.	.	.
Zdr-1	6984	.	.	.	.	.	.	.	.	.	.	.	.	.	.	.	.	.	.	.	.
Zdr-6	6985	.	.	.	.	.	.	.	.	.	.	.	.	.	.	.	.	.	.	.	.

Accessions: native name and ecotype ID

Br-0	6904	.	G	A	A	A	.	G	T	C	.	.	.	T	.	.	.	.	.	.	.
C24	6906	.	G	A	.	A	.	G	T	C	.	.	.	T	.	.	.	.	.	.	.
NFA-10	6943	.	G	A	.	A	.	G	T	C	.	.	.	T	.	.	T	.	.	.	.
Ag-0	6897	.	.	.	.	.	.	.	.	.	.	.	.	.	.	.	.	.	.	.	.
Kno-18	6928	.	.	.	.	.	.	.	.	.	.	.	.	.	.	.	.	.	.	.	.
Pro-0	8213	.	.	.	.	.	.	.	.	.	-	.	.	.	.	.	.	.	.	.	.
Ra-0	6958	.	.	.	.	.	.	.	.	.	.	.	.	.	.	.	.	.	.	.	.
Var-2-1	7516	.	.	.	.	.	.	.	.	.	.	.	.	.	.	.	.	.	.	.	.
Var-2-6	7517	.	.	.	.	.	.	.	.	.	.	.	.	.	.	.	.	.	.	.	.
Wt-5	6982	.	.	.	.	.	.	.	.	.	.	.	.	.	.	.	.	.	.	.	.
Ct-1	6910	.	.	.	.	.	.	.	.	.	.	.	.	.	.	.	.	.	.	.	.
Ga-0	6919	.	.	.	.	.	.	.	.	.	.	.	.	.	.	.	.	.	.	.	.
Gy-0	8214	.	.	.	.	.	.	.	.	.	.	.	.	.	.	.	.	.	.	.	.
LL-0	6933	.	.	.	.	.	.	.	.	.	.	.	.	.	.	.	.	.	.	.	.
Ts-5	6971	.	.	.	.	.	.	.	.	.	.	.	.	.	.	.	.	.	.	.	.
Est-1	6916	.	.	.	.	.	.	.	.	.	.	.	.	.	.	.	.	.	.	A	.
Gu-0	7149	.	.	.	.	.	.	.	.	.	.	.	.	.	.	.	.	.	.	A	.
Kno-10	6927	.	.	.	.	.	.	.	.	.	.	.	.	.	.	.	.	.	.	A	.
Kz-1	6930	.	.	.	.	.	.	.	.	.	.	.	.	.	.	.	.	.	.	A	.
Kz-9	6931	.	.	.	.	.	.	.	.	.	.	.	.	.	.	A	.	.	.	A	.
Lp2-6	7521	.	.	.	.	.	.	.	.	.	.	.	.	.	.	.	.	.	.	A	.
Mr-0	7522	.	.	.	.	.	.	.	.	.	.	.	.	.	.	.	.	.	.	A	.
Mrk-0	6937	.	.	.	.	.	.	.	.	.	.	.	.	.	.	.	.	.	.	A	.
Ms-0	6938	.	.	.	.	.	.	.	.	.	.	.	.	.	.	.	.	.	.	A	.
Nd-1	6942	.	.	.	.	.	.	.	.	.	.	.	.	.	A	.	.	.	G	A	.
Pna-10	7526	.	.	.	.	.	.	.	.	.	.	.	.	.	.	.	.	.	.	A	.
Pna-17	7523	.	.	.	.	.	.	.	.	.	.	.	.	.	.	.	.	.	.	A	.
RRS-10	7515	.	.	.	.	.	.	.	.	.	.	.	.	.	.	.	.	.	.	A	.
Shahdara	6962	.	.	.	.	.	.	.	.	.	.	.	.	.	.	.	.	.	.	A	.
Uod-7	6976	.	.	.	.	.	.	.	.	.	.	.	.	.	.	.	.	.	.	A	.
Ws-0	6980	.	.	.	.	.	.	.	.	.	.	.	.	.	.	.	.	.	.	A	.
Yo-0	6983	.	.	.	.	.	.	.	.	.	.	.	.	.	.	.	.	.	.	A	.
Bur-0	6905	.	.	.	.	.	.	.	.	.	.	.	C	.	.	.	.	.	.	A	A
CIBC-5	6908	.	.	.	.	.	.	.	.	.	.	.	C	.	.	.	.	.	.	A	.
Eden-1	6009	.	.	.	.	.	.	.	.	.	.	.	C	.	.	.	.	.	.	A	.
Eden-2	6913	.	.	.	.	.	.	.	.	.	.	.	C	.	.	.	.	.	.	A	.
Edi-0	6914	.	.	.	.	.	.	.	.	.	.	.	C	.	.	.	.	.	.	A	.
Fab-2	6917	.	.	.	.	.	.	.	.	.	.	.	C	.	.	.	.	.	.	A	.
Fab-4	6918	.	.	.	.	.	.	.	.	.	.	.	C	.	.	.	.	.	.	A	.

Accessions: native name and ecotype ID

Got-7	6921	.	.	.	.	.	.	.	.	.	.	.	C	.	.	.	.	.	.	A	.
Got-22	6920	.	.	.	.	.	.	.	.	.	.	.	C	.	.	.	.	.	.	A	.
Nok-3	6945	.	.	.	.	.	.	.	.	.	.	.	C	.	.	.	.	.	.	A	.
Oy-0	6946	.	.	.	.	.	.	.	.	.	.	.	C	.	.	.	.	.	.	A	.
Spr-1-2	6964	.	.	.	.	.	.	.	.	.	.	.	C	.	.	.	.	.	.	A	.
Spr-1-6	6965	.	.	.	.	.	.	.	.	.	.	.	C	.	.	.	.	.	.	A	.
Sq-1	6966	.	.	.	.	.	.	.	.	.	.	.	C	.	.	.	.	.	.	A	.
Ull-2-3	6973	.	.	.	.	.	.	.	.	.	.	.	C	.	.	.	.	.	.	A	.
Cvi-0	6911	C	G	A	.	A	.	G	T	.	.	G	.	T	.	.	.	.	.	.	.
Kas-1	na	.	G	A	.	A	.	G	T	.	.	.	.	T	.	.	.	.	.	.	.
Kondara	6929	.	G	A	.	A	.	G	T	.	.	.	.	T	.	.	.	.	.	.	.
Sorbo	6963	.	G	A	.	A	.	G	T	.	.	.	.	T	.	.	.	.	.	.	.
Bay-0	6899	.	.	.	.	.	C	.	.	.	.	.	.	.	.	.	.	--	.	.	.
Fei-0	8215	.	.	.	.	.	C	.	.	.	.	.	.	.	.	.	.	--	.	.	.
HR-5	6924	.	.	.	.	.	C	.	.	.	.	.	.	.	.	.	.	--	.	.	.
HR-10	6923	.	.	.	.	.	C	.	.	.	.	.	.	.	.	.	.	--	.	.	.
Kin-0	6926	.	.	.	.	.	C	.	.	.	.	.	.	.	.	.	.	--	.	.	.
Lz-0	6936	.	.	.	.	.	C	.	.	.	.	.	.	.	.	.	.	--	.	.	.
NFA-8	6944	.	.	.	.	.	C	.	.	.	.	.	.	.	.	.	.	--	.	.	.
Ren-1	6959	.	.	.	.	.	C	.	.	.	.	.	.	.	.	.	.	--	.	.	.
Ren-11	6960	.	.	.	.	.	C	.	.	.	.	.	.	.	.	.	.	--	.	.	.
Rmx-A02	7524	.	.	.	.	.	C	.	.	.	.	.	.	.	.	.	.	--	.	.	.
Rmx-A180	7525	.	.	.	.	.	C	.	.	.	.	.	.	.	.	.	.	--	.	.	.
RRS-7	7514	.	.	.	.	.	C	.	.	.	.	.	.	.	.	.	.	--	.	.	.
Sq-8	6967	.	.	.	.	.	C	.	.	.	.	.	.	.	.	.	.	--	.	.	.
An-1	6898	.	.	.	.	.	.	.	.	.	.	.	.	.	.	.	.	.	.	A	.
Bil-5	6900	.	.	.	.	.	.	.	.	.	.	.	.	.	.	.	.	.	.	A	.
Bil-7	6901	.	.	.	.	.	.	.	.	.	.	.	.	.	.	.	.	.	.	A	.
Ei-2	6915	.	.	.	.	.	.	.	.	.	.	.	.	.	.	.	.	.	.	A	.
Se-0	6961	.	.	.	.	.	.	.	.	.	.	.	.	.	.	.	.	.	.	A	.
Ts-1	6970	.	.	.	.	.	.	.	.	.	.	.	.	.	.	.	.	.	.	A	.
Wei-0	6979	.	.	.	.	.	.	.	.	.	.	.	.	.	.	.	.	.	.	A	.

Grey cells: *At3g57700* ; blue cells: *At3g57710* (*RKS1*); yellow cells: *At3g57720* ; green cells: intergenic regions; orange cells: indels.
Nucleotide positions in bold indicate SNPs which are part of the set of 214,051 SNPs.
Ecotype ID retrieved from 1,307 natural accessions genotyped using a 250K SNP chip (ref. 16). na: not available.
