# Supplementary material for: An Atypical Kinase under Balancing Selection Confers Broad-Spectrum Disease Resistance in Arabidopsis
Source: PLoS Genet. 2013 Sep 12;9(9):e1003766. doi: 10.1371/journal.pgen.1003766 (PMC3772041; doi:10.1371/journal.pgen.1003766)
Supplement: Table S9 — Fitting of one linear model and two non-linear models on the relationship between disease index and expression levels of RKS1 in natural accessions. (PDF) [file pgen.1003766.s025.pdf]

Table S9. Fitting of one linear model and two non-linear models on the relationship between disease index and expression levels of *RKS1* in natural accessions. Similar results were obtained after excluding the Omo2-1 outlier accession (see Figure S13) from the statistical analyses.

		lm		exp		log	
accessions	expression	intercept	a	A	k	b	c
all	*RKS1-L*	0.829 ***	-5.064 ***	0.966 ***	11.775 **	-0.165 ***	0.019 ns
all	*RKS1-L+S*	0.854 ***	-0.903 **	0.897 ***	1.544 **	-0.172 **	0.331 **
w/o stop codon	*RKS1-L*	0.802 ***	-5.299 ***	1.033 ***	16.189 **	-0.179 ***	-0.075 ns
w/o stop codon	*RKS1-L+S*	0.855 ***	-1.074 **	0.939 ***	2.115 **	-0.205 **	0.230 *
w/o additional S alleles	*RKS1-L*	0.712 ***	-4.136 ***	0.810 ***	10.550 *	-0.215 **	-0.187 ns
w/o additional S alleles	*RKS1-L+S*	0.881 ***	-1.331 ***	1.023 ***	2.919 ***	-0.247 ***	-0.096 ns

lm: linear model (disease ~ intercept + a*expression); exp: exponential function (disease ~ $Ae^{-k*expression}$); log: logarithmic function (disease ~ b*log(expression) + c). *0.05 > *P* > 0.01, **0.01 > *P* > 0.001, ****P* < 0.001.

accessions: ‘all’: all accessions for which expression level of *RKS1* is available; ‘w/o stop codon’: all accessions but accessions with the stop codon at the fourth amino-acid in *RKS1*; ‘w/o additional S alleles’: all accessions but accessions with the stop codon or belonging to the second S susceptible allele embedded in the R intergenic haplogroup.

expression: ‘*RKS1-L*’: Relative gene expression (A.U.) of the *RKS1* long transcript; ‘*RKS1-L+S*’: Relative gene expression (A.U.) of *RKS1* total mRNA.
