# Supplementary material for: An Atypical Kinase under Balancing Selection Confers Broad-Spectrum Disease Resistance in Arabidopsis
Source: PLoS Genet. 2013 Sep 12;9(9):e1003766. doi: 10.1371/journal.pgen.1003766 (PMC3772041; doi:10.1371/journal.pgen.1003766)
Supplement: Table S10 — Model selection among one linear model and two non-linear models on the relationship between disease index and expression levels of RKS1 in natural accessions. (PDF) [file pgen.1003766.s026.pdf]

**Table S10.** Model selection among one linear model and two non-linear models on the relationship between disease index and expression levels of *RKS1* in natural accessions. Green values indicate a better fit of the logarithmic function in comparison to the two other models. Yellow values indicate a better fit of the exponential function in comparison to the linear model Similar results were obtained after excluding the Omo2-1 outlier accession (see Figure S13) from the statistical analyses.

| | | | | lm | | | exp | | | log | | |
|---|---|---|---|---|---|---|---|---|---|---|---|---|
| accessions | expression | n | k | AIC | BIC | AICc | AIC | BIC | AICc | AIC | BIC | AICc |
| all | *RKS1-L* | 88 | 2 | 121.06 | 128.50 | 121.20 | 119.64 | 127.07 | 119.78 | 115.45 | 122.88 | 115.59 |
| all | *RKS1-L+S* | 88 | 2 | 123.79 | 131.22 | 123.93 | 123.98 | 131.41 | 124.12 | 124.44 | 131.88 | 124.58 |
| w/o stop codon | *RKS1-L* | 75 | 2 | 106.97 | 113.93 | 107.14 | 103.63 | 110.58 | 103.80 | 99.78 | 106.74 | 99.95 |
| w/o stop codon | *RKS1-L+S* | 75 | 2 | 107.62 | 114.57 | 107.79 | 107.42 | 114.37 | 107.59 | 108.19 | 115.14 | 108.36 |
| w/o additional S alleles | *RKS1-L* | 72 | 2 | 93.60 | 100.43 | 93.77 | 93.16 | 99.99 | 93.33 | 94.07 | 100.90 | 94.24 |
| w/o additional S alleles | *RKS1-L+S* | 72 | 2 | 80.32 | 87.15 | 80.49 | 79.81 | 86.64 | 79.98 | 82.50 | 89.33 | 82.67 |

lm: linear model (disease ~ intercept + a*expression); exp: exponential function (disease ~ $Ae^{-k*expression}$); log: logarithmic function (disease ~ b*log(expression) + c). 'n' and 'k' denote the sample size and the number of parameters, respectively. AIC: Akaike's information criterion; BIC Bayesian information criterion; AICc: corrected AICc.

accessions: 'all': all accessions for which expression level of *RKS1* is available; 'w/o stop codon': all accessions but accessions with the stop codon at the fourth amino-acid in *RKS1*; 'w/o additional S alleles': all accessions but accessions with the stop codon or belonging to the second S susceptible allele embedded in  the R intergenic haplogroup.

expression: '*RKS1-L*': Relative gene expression (A.U.) of the *RKS1* long transcript; '*RKS1-L+S*': Relative gene expression (A.U.) of *RKS1* total mRNA.
